# Supplementary material for: Mesitylated trityl radicals, a platform for doublet emission: symmetry breaking, charge-transfer states and conjugated polymers
Source: Nat Commun. 2023 Jul 12;14:4147. doi: 10.1038/s41467-023-39834-2 (PMC10338495; doi:10.1038/s41467-023-39834-2)
Supplement: Supplementary file 1 — Supplementary Information [file 41467_2023_39834_MOESM1_ESM.pdf]

SUPPLEMENTARY INFORMATION

Mesitylated trityl radicals, a platform for doublet emission: symmetry breaking, charge-transfer states and conjugated polymers

Petri Murto,[1] Rituparno Chowdhury,[2,†] Sebastian Gorgon,[2,†] Erjuan Guo,[2,†] Weixuan Zeng,[1,†] Biwen Li,[2] Yuqi Sun,[2] Haydn Francis,[1] Richard H. Friend*[2] and Hugo Bronstein*[1,2]

[1] Yusuf Hamied Department of Chemistry, University of Cambridge, Cambridge CB2 1EW, UK.

[2] Cavendish Laboratory, University of Cambridge, Cambridge CB3 0HE, UK.

[†] These authors contributed equally: R. Chowdhury, S. Gorgon, E. Guo, W. Zeng.

*e-mail: hab60@cam.ac.uk; rhf10@cam.ac.uk

Table of Contents

Supplementary Figures S3

Supplementary Note 1: Materials and Synthesis S9

Supplementary Note 2: NMR Spectra S25

Supplementary Note 3: X-Ray Crystallography S43

Supplementary Note 4: Radical Conversion and α-Hydrogenation S47

Supplementary Note 5: DFT Calculations S60

Supplementary Note 6: Cyclic Voltammetry S79

Supplementary Note 7: Optical Spectroscopy S85

Supplementary Note 8: Thermal Stability S92

Supplementary References S93

Supplementary Figures

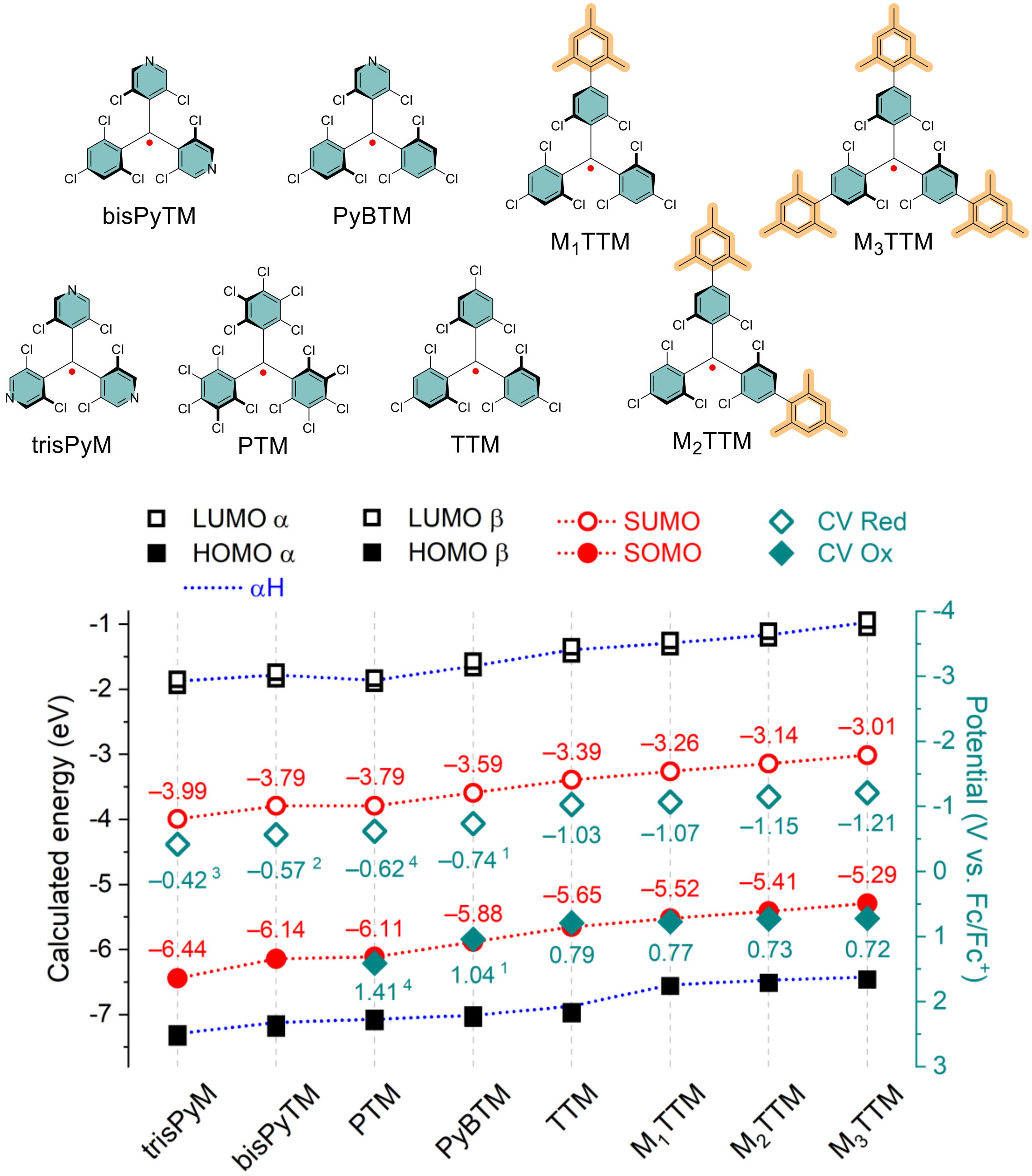

Supplementary Fig. 1 Chemical structures of trityl radical derivatives and evolution of their calculated frontier molecular orbital energy levels and electrochemical redox potentials. The singly occupied molecular orbitals (SOMOs) are assigned as occupied and unoccupied (SOMO and SUMO, respectively) as indicated in the legend. The blue dotted lines represent the energy levels calculated for the corresponding αH precursors. Redox potentials for PyBTM, bisPyTM and trisPyM are obtained from refs.[1-3] while the values for PTM are obtained from ref.[4] using literature conversion values for Ag/Ag^{+} *vs.* SCE and Fc/Fc^{+} *vs.* SCE (SCE, saturated calomel electrode).[5,6]

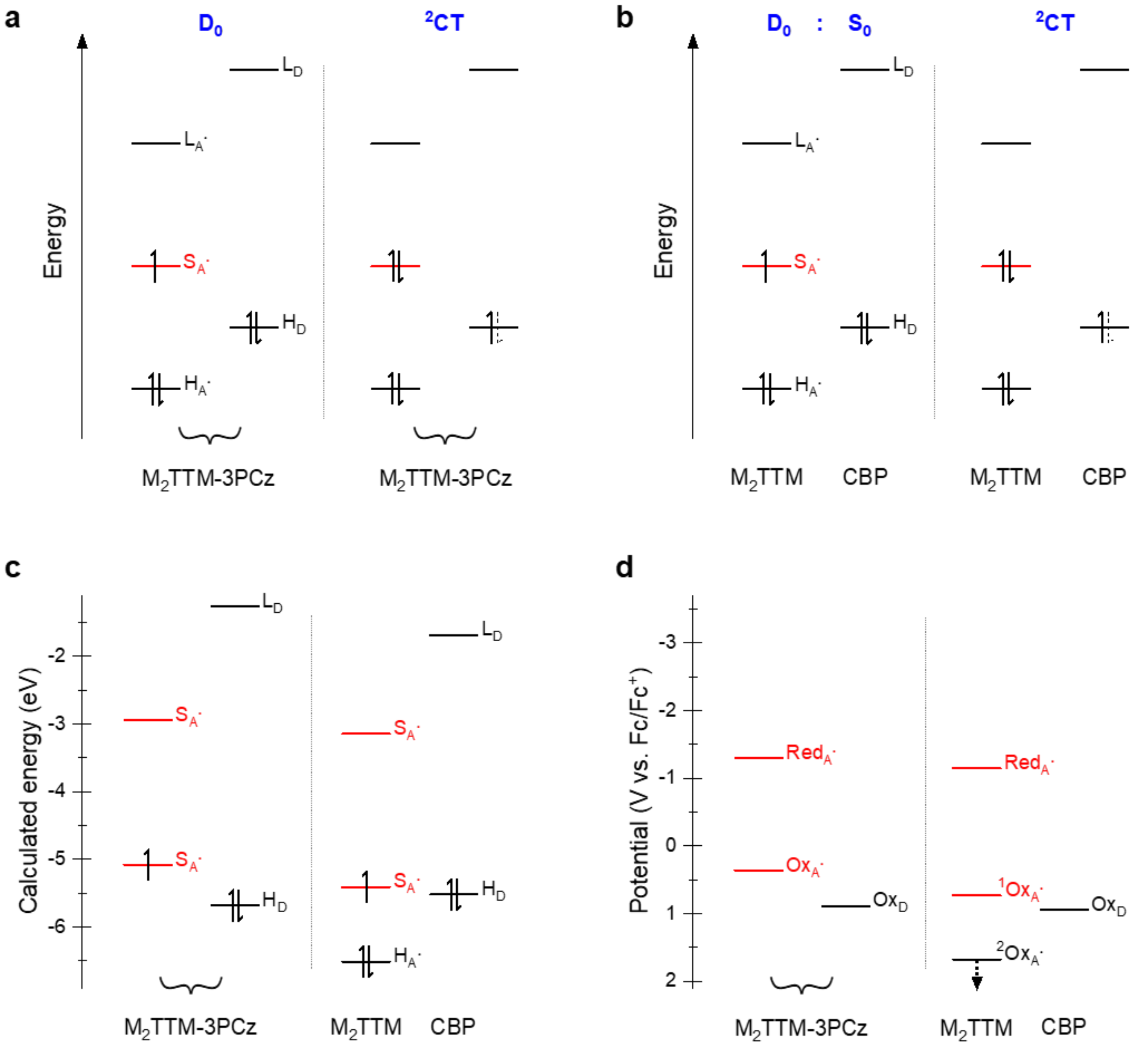

Supplementary Fig. 2 Electronic structures of M_2TTM-3PCz and M_2TTM:CBP blend. Schematic doublet ground state (D_0) and the doublet charge transfer state (^{2}CT) in **a**, M_2TTM-3PCz and **b**, its intermolecular analogue M_2TTM:CBP blend, with electron occupancy shown by half-headed arrows and hole indicated by the dashed arrow. **c**, Calculated molecular orbital energy levels and **d**, electrochemically accessible redox states of M_2TTM-3PCz, M_2TTM and CBP (H, HOMO; S, SOMO/SUMO; L, LUMO; Ox, oxidation; Red, reduction; D, donor; $A^•$, acceptor).

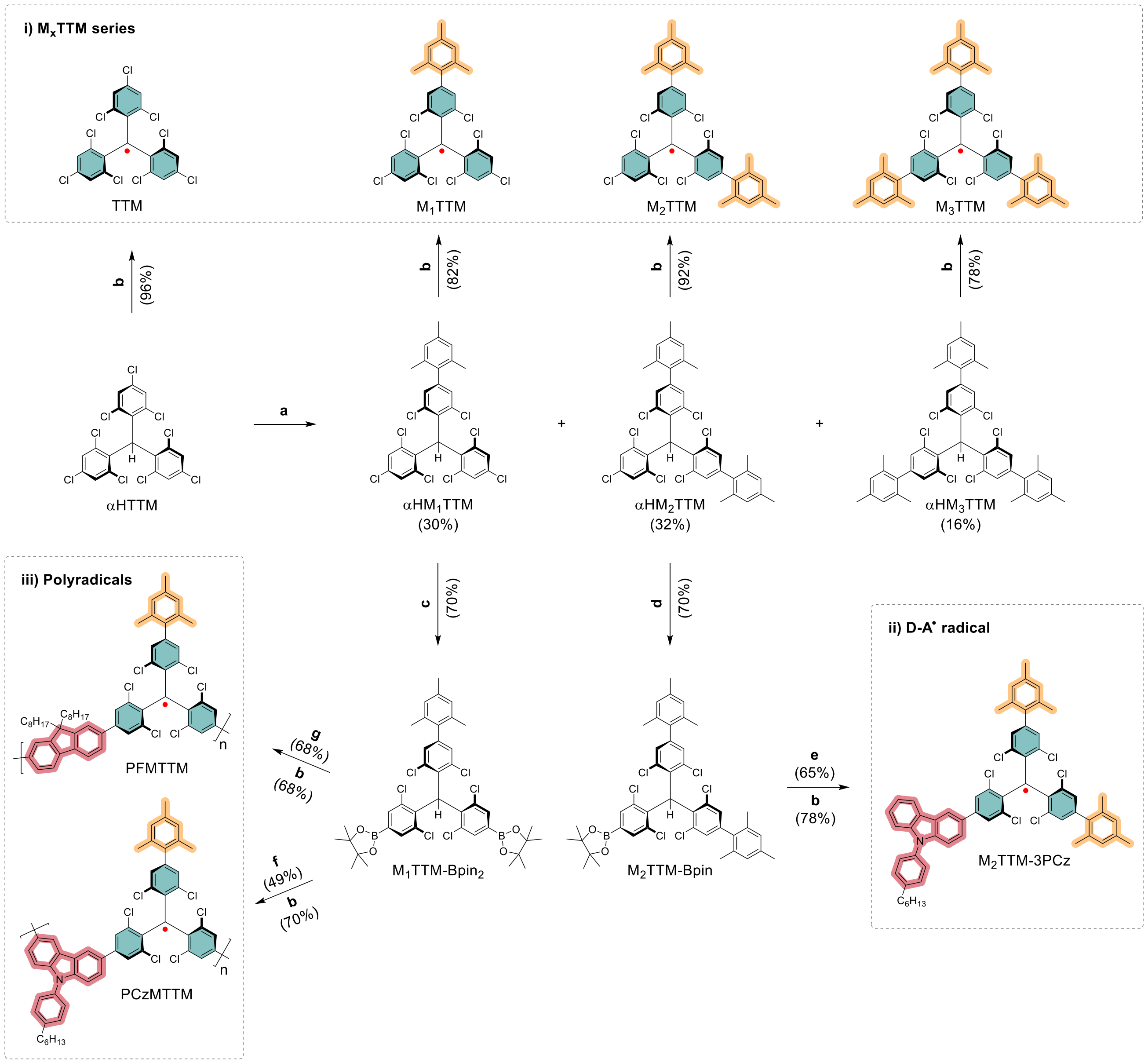

Supplementary Fig. 3 Synthesis of the three radical families (i–iii). Entries *a*–*h*: [a] Mesitylboronic acid (3 equiv.), K_3PO_4 (4.8 equiv.), $Pd(OAc)_2$ (0.02 equiv.), SPhos (0.04 equiv.), 1,4-dioxane (anhyd.), 80 °C, 26 h. [b] 1. 40% Bu_4NOH (aq, 2 equiv.), DMSO/THF 3:1 (v/v), dark, RT, 4–6 h (small molecules), 72 h (polymers); 2. *p*-chloranil (2.5 equiv.), dark, RT, 1–6 h (small molecules), 12 h (polymers). [c] Bis(pinacolato)diboron (3 equiv.), KOAc (3.6 equiv.), $Pd(dba)_2$ (0.02 equiv.), SPhos (0.05 equiv.), 1,4-dioxane (anhyd.), 80 °C, 24 h. [d] Bis(pinacolato)diboron (1.5 equiv.), KOAc (1.8 equiv.), $Pd(dba)_2$ (0.02 equiv.), SPhos (0.05 equiv.), 1,4-dioxane (anhyd.), 80 °C, 24 h. [e] M_2TTM-Bpin (1.2 equiv.), 3-bromo-9-(4-hexylphenyl)-9*H*-carbazole (**S2**, 1 equiv.), K_3PO_4 (1.4 equiv.), $Pd(OAc)_2$ (0.03 equiv.), SPhos (0.075 equiv.), 1,4-dioxane (anhyd.), 80 °C, 24 h. [f,g] M_1TTM-$Bpin_2$ (1 equiv.), 3,6-dibromo-9-(4-hexylphenyl)-9*H*-carbazole (**S3**, 1 equiv., entry *f*) or 2,7-dibromo-9,9-dioctyl-9*H*-fluorene (1 equiv., entry *g*), K_3PO_4 (4 equiv.), $Pd(OAc)_2$ (0.01 equiv.), SPhos (0.02 equiv.), 1,4-dioxane (anhyd.), 80 °C, 72 h.

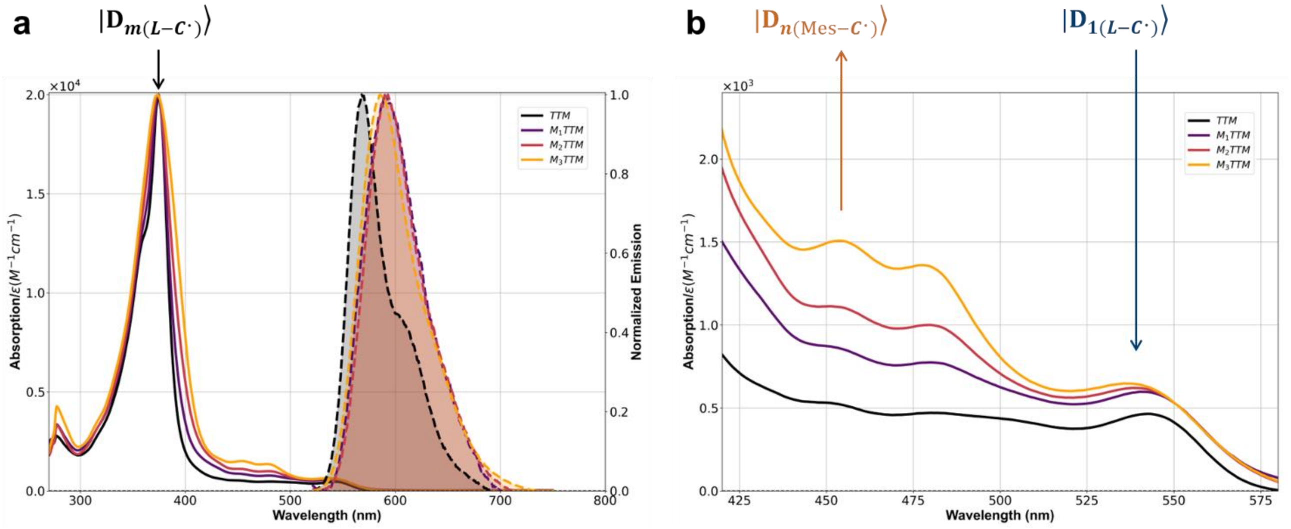

Supplementary Fig. 4 Optical spectroscopy of M$_x$TTM series. a, Absorption (solid lines) and PL (broken lines) spectra in 0.1 mM toluene solution. **b**, Close-up of the low-energy absorption region.

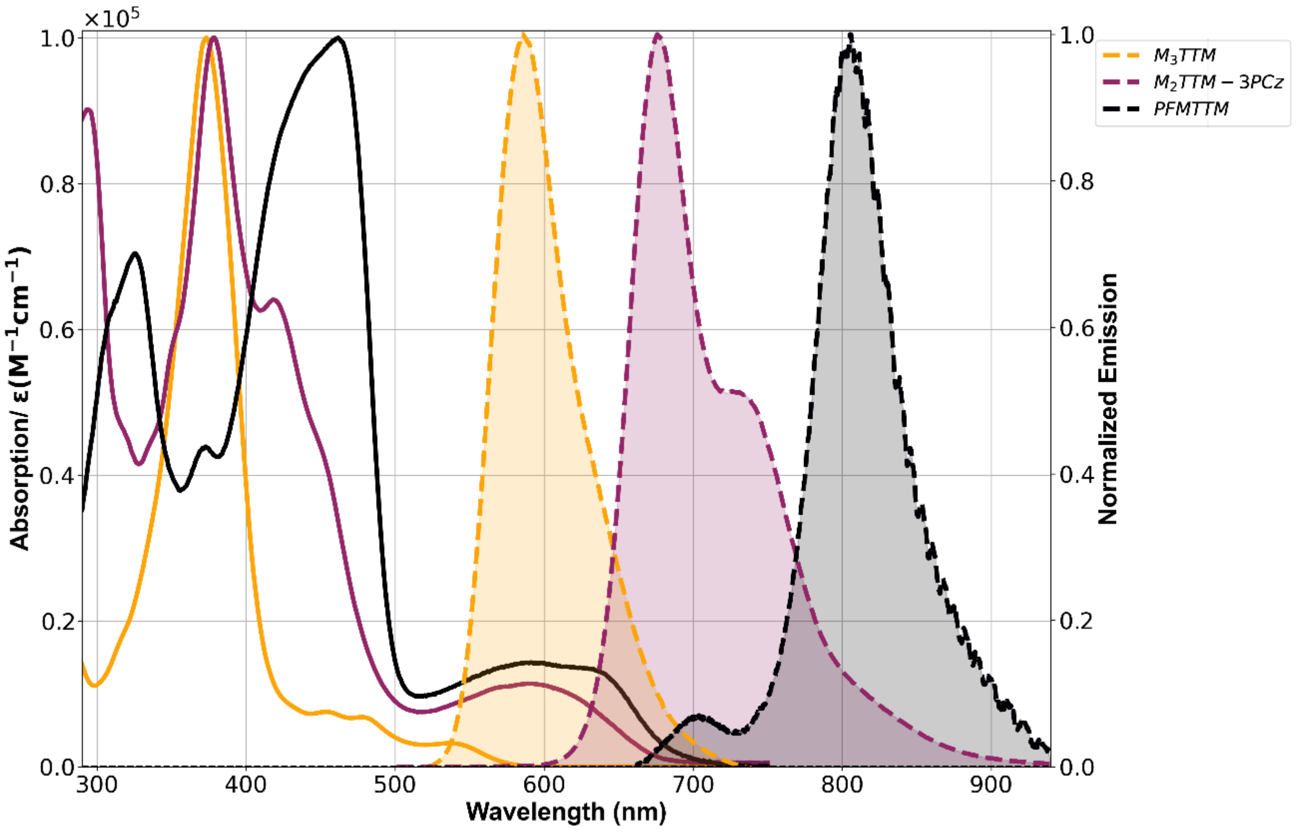

Supplementary Fig. 5 Optical spectroscopy of the radical families. Absorption (solid lines) and PL (broken lines) spectra of M_3TTM, M_2TTM-3PCz and PFMTTM in 0.1 mM toluene solution.

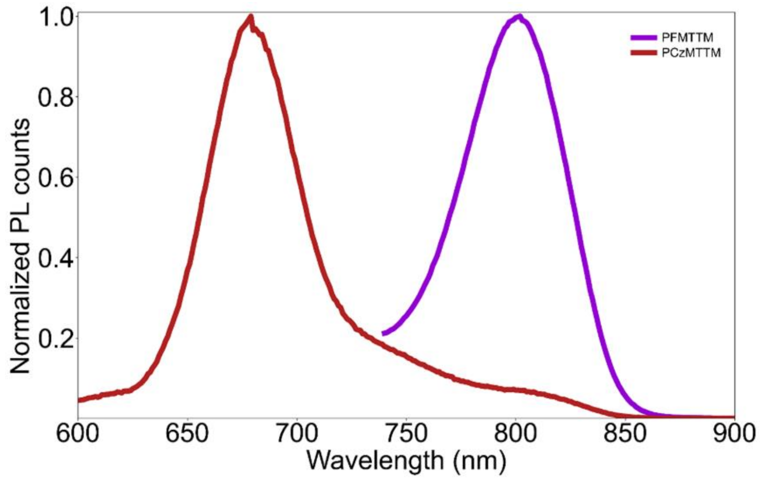

Supplementary Fig. 6 Emission spectra of polyradicals. Photoluminescence upon 550 nm excitation of spin-coated films of PFMTTM (violet) and PCzMTTM (red) cast on spectrosil substrates in a glovebox from 12mg/mL toluene solutions at 2000 rpm for 45 s followed by annealing at 100 °C for 10 min. These films were subsequently encapsulated in the glovebox to prevent exposure to air during experiments.

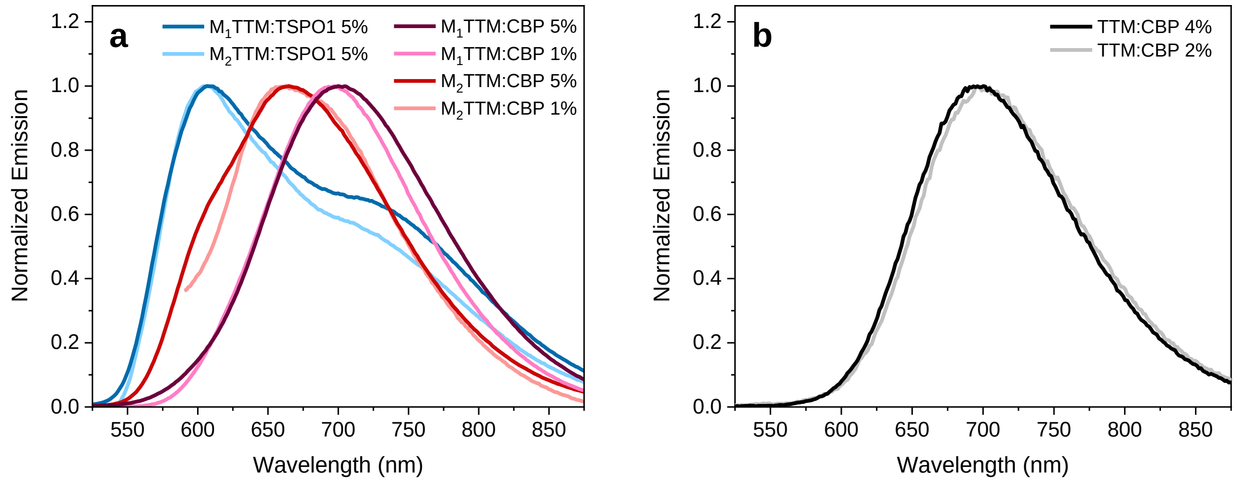

Supplementary Fig. 7 Emission spectra of M_xTTM in CBP and TSPO1 hosts. a, Steady-state photoluminescence of evaporated M_1TTM and M_2TTM films in CBP (1 and 5 wt%) and in TSPO1 (5 wt%) following 520 nm excitation. **b**, Steady-state photoluminescence of evaporated TTM films in CBP (2 and 4 wt%) following 520 nm excitation. TSPO1 was used as a wide band gap host material that does not facilitate host to radical charge transfer.[7]

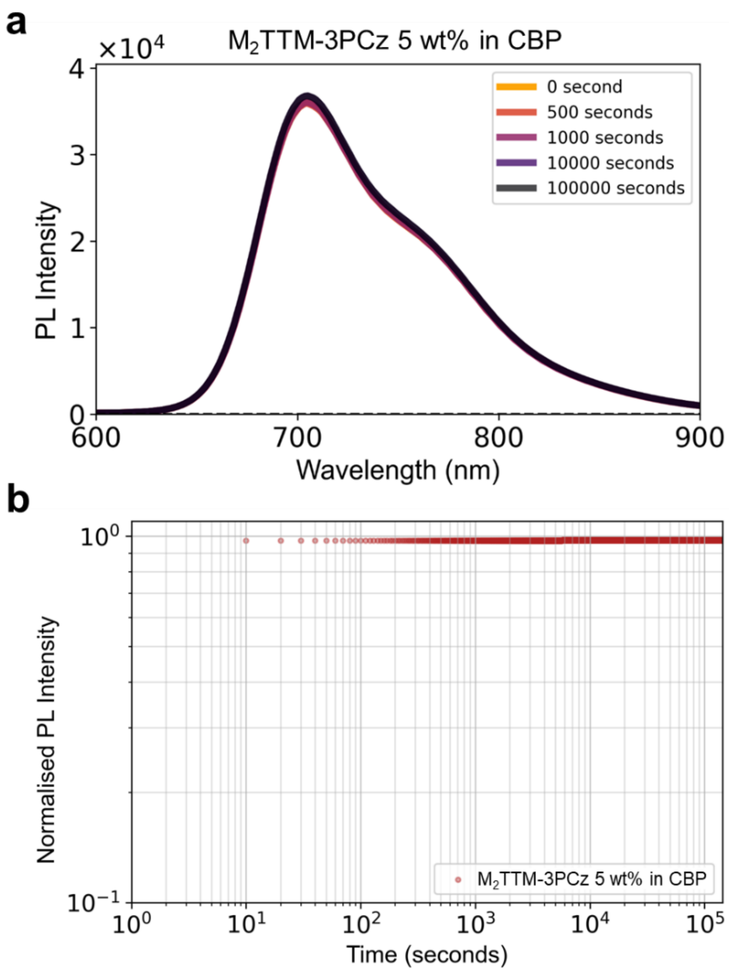

Supplementary Fig. 8 Photostability of evaporated film of 5 wt% M_2TTM-3PCz in CBP. a, Photoluminescence and **b**, photoluminescence peak intensity during 100 000 s (ca. 28 h) continuous wave excitation at 532 nm under ambient conditions.

Supplementary Note 1: Materials and Synthesis

The monomers 9*H*-carbazole, 2-bromo-9*H*-carbazole, 2,7-dibromo-9*H*-carbazole and 2,7-dibromo-9,9-dioctyl-9*H*-fluorene were obtained from commercial sources (Merck, Ossila and Fluorochem) and used as received. Other reagents, catalysts and (anhydrous) solvents were purchased from Merck, Alfa Aesar and Acros Organics and used as received.

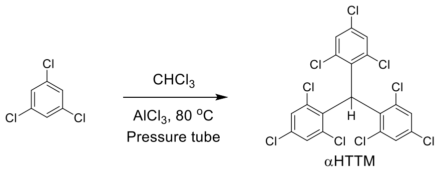

Tris(2,4,6-trichlorophenyl)methane (αHTTM). Following modified literature procedures,[8,9] 1,3,5-trichlorobenzene (41.033 g, 226.15 mmol, 9 equiv.) was added into a glass pressure tube and the tube was purged with Ar gas. Anhydrous $AlCl_3$ (3.685 g, 27.64 mmol, 1.1 equiv.) was added, followed by anhdydrous $CHCl_3$ (2.0 mL, 25.13 mmol, 1 equiv.). The tube was sealed and heated at 80 °C oil bath for 3 h. After cooling to RT, the tube was carefully opened to release the formed HCl gas (hazard: highly pressurized HCl gas). The solidified reaction mixture was dissolved in $CHCl_3$ and poured over ice water. The organic phase was extracted with water (3 × 200 mL) and dried over anhydrous $MgSO_4$. Solvent was removed under vacuum and the crude product was purified with column chromatography over silica gel, by gradually increasing the eluent polarity from hexane (fraction 1) to 20% (v/v) DCM in hexane (fraction 2). The target compound was collected as fraction 2. Solvent was removed and the product was recrystallised from hexane/DCM mixture by slow evaporation of DCM. After removing the solvent in vacuum, white solid was collected (12.514 g, 90%). ^{1}H NMR (400 MHz, $CDCl_3$) δ 7.36 (d, J = 2.2 Hz, 3H), 7.23 (d, J = 2.2 Hz, 3H), 6.68 (s, 1H). ^{13}C NMR (100 MHz, $CDCl_3$) δ 138.07, 137.24, 133.99, 133.94, 130.16, 128.59, 49.97. TOF-MS-ASAP$^+$ Calcd. for $[C_{19}H_8Cl_9]^+$: 550.7823. Found: m/z = 550.7811. EA Calcd. for $C_{19}H_7Cl_9$: C, 41.17; H, 1.27; Cl, 57.56. Found: C, 41.10; H, 1.18; N, 0.00.

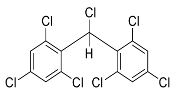

S1

2,2'-(Chloromethylene)bis(1,3,5-trichlorobenzene) (S1). The title compound was a minority by-product (fraction 1) in the above reaction and was collected as white solid (0.748 g, 7%). ^{1}H NMR (600 MHz, $CDCl_3$) δ 7.36 (s, 4H), 7.02 (s, 1H). ^{13}C NMR (150 MHz, $CDCl_3$) δ 136.85, 134.94, 132.13, 129.93, 55.45. TOF-MS-ASAP^{-} Calcd. for $[C_{13}H_4Cl_7]^{-}$: 404.8133. Found: *m/z* = 404.8116. EA Calcd. for $C_{13}H_5Cl_7$: C, 38.15; H, 1.23; Cl, 60.62. Found: C, 38.09; H, 1.16; N, 0.00.

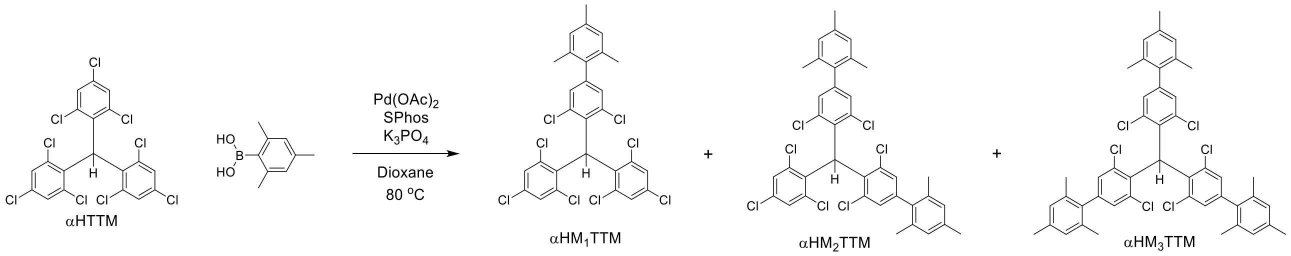

General procedure for the synthesis of αHM$_x$TTM precursors. αHTTM (3.400 g, 6.13 mmol, 1 equiv.), mesitylboronic acid (3.018 g, 18.40 mmol, 3 equiv.), $Pd(OAc)_2$ (0.0275 g, 0.123 mmol, 0.02 equiv.), SPhos (0.1007 g, 0.245 mmol, 0.04 equiv.) and K_3PO_4 (6.250 g, 29.44 mmol, 4.8 equiv.) were added into a microwave vial and the vial was subjected to three vacuum/Ar gas refill cycles. Anhydrous 1,4-dioxane (37 mL) was added and the mixture was heated at 80 °C oil bath for 26 h. After cooling to RT, the mixture was diluted with hexane, extracted with water (3 × 200 mL) and the organic phase was dried over anhydrous $MgSO_4$. Solvent was removed under vacuum and the crude product was purified with column chromatography over silica gel, by gradually increasing the eluent polarity from hexane (fractions 1 and 2) to 2% (v/v) DCM in hexane (fraction 3) and finally to 5% (v/v) DCM in hexane (fraction 4). Fraction 1 was the starting material αHTTM (0.605 g, 18%). The target compounds were obtained as fractions 2–4. For each fraction, solvent was removed and the solids were sonicated in MeOH for 5 min. The solids were collected by centrifuging and MeOH was removed by pipetting. Finally, the solids were dried in vacuum.

4'-(Bis(2,4,6-trichlorophenyl)methyl)-3',5'-dichloro-2,4,6-trimethyl-1,1'-biphenyl (αHM$_1$TTM) was collected as fraction 2 as white solid (1.172 g, 30%). An upscaled reaction yielded 4.416 g (32%). ^{1}H NMR (400 MHz, $CDCl_3$) δ 7.38 (dd, J = 3.8, 2.1 Hz, 2H), 7.25 (t, 2H), 7.14 (d, J = 1.7 Hz, 1H), 7.01 (d, J = 1.8 Hz, 1H), 6.93 (s, 2H), 6.80 (s, 1H), 2.32 (s, 3H), 2.02 (d, J = 4.3 Hz, 6H). ^{13}C NMR (100 MHz, $CDCl_3$) δ 142.60, 138.17, 138.09, 137.64, 137.52, 137.33, 136.78, 135.92, 135.71, 135.68, 134.61, 134.56, 133.71, 133.69, 133.15, 131.21, 130.27, 130.07, 129.50, 128.59, 128.51, 128.35, 128.34, 50.20, 21.18, 20.66. TOF-MS-ASAP$^+$ Calcd. for $[C_{28}H_{19}Cl_8]^+$: 634.8995. Found: m/z = 634.9011. EA Calcd. for $C_{28}H_{18}Cl_8$: C, 52.71; H, 2.84; Cl, 44.45. Found: C, 52.60; H, 2.69; N, 0.00.

4',4'''-((2,4,6-Trichlorophenyl)methylene)bis(3',5'-dichloro-2,4,6-trimethyl-1,1'-biphenyl) (αHM$_2$TTM) was collected as fraction 3 as white solid (1.419 g, 32%). An upscaled reaction yielded 4.966 g (32%). ^{1}H NMR (400 MHz, $CDCl_3$) δ 7.40 (d, J = 2.3 Hz, 1H), 7.27 (d, J = 2.3 Hz, 1H), 7.16 (dd, J = 6.2, 1.8 Hz, 2H), 7.03 (t, J = 2.1 Hz, 2H), 6.94 (s, 4H), 6.91 (s, 1H), 2.33 (s, 6H), 2.05 (dd, J = 4.7, 3.2 Hz, 12H). ^{13}C NMR (100 MHz, $CDCl_3$) δ 142.37, 142.35, 138.24, 137.62, 137.59, 137.57, 137.50, 137.44, 136.94, 136.81, 136.07, 136.06, 135.79, 135.74, 135.21, 133.80, 133.67, 133.45, 131.27, 131.15, 130.18, 129.47, 129.46, 128.53, 128.34, 128.31, 50.45, 21.19, 20.72, 20.69, 20.67. TOF-MS-ASAP$^+$ Calcd. for $[C_{37}H_{30}Cl_7]^+$: 719.0167. Found: m/z = 719.0159. EA Calcd. for $C_{37}H_{29}Cl_7$: C, 61.57; H, 4.05; Cl, 34.38. Found: C, 61.49; H, 3.97; N, 0.00.

Tris(3,5-dichloro-2',4',6'-trimethyl-[1,1'-biphenyl]-4-yl)methane (αHM$_3$TTM) was collected as fraction 4 as white solid (0.783 g, 16%). An upscaled reaction yielded 2.641 g (15%). ^{1}H NMR (400 MHz, $CDCl_3$) δ 7.18 (d, J = 1.8 Hz, 3H), 7.04 (d, J = 1.8 Hz, 3H), 7.01 (s, 1H), 6.95 (s, 6H), 2.33 (s, 9H), 2.07 (d, J = 1.8 Hz, 18H). ^{13}C NMR (100 MHz, $CDCl_3$) δ 142.12, 137.64, 137.51, 136.96, 136.21, 135.87, 135.81, 134.35, 131.20, 129.44, 128.31, 128.28, 50.70, 21.20, 20.75, 20.73. TOF-MS-ASAP$^+$ Calcd. for $[C_{46}H_{41}Cl_6]^+$: 803.1339. Found: m/z = 803.1374. EA Calcd. for $C_{46}H_{40}Cl_6$: C, 68.59; H, 5.01; Cl, 26.41. Found: C, 68.50; H, 4.99; N, 0.00.

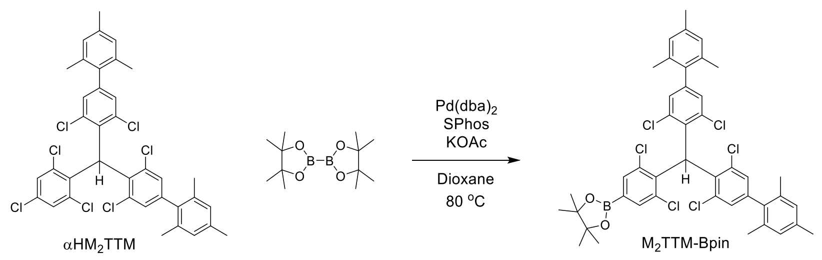

2-(4-(Bis(3,5-dichloro-2',4',6'-trimethyl-[1,1'-biphenyl]-4-yl)methyl)-3,5-dichlorophenyl)-4,4,5,5-tetramethyl-1,3,2-dioxaborolane (M_2TTM-Bpin). αHM_2TTM (1.000 g, 1.39 mmol, 1 equiv.), bis(pinacolato)diboron (0.528 g, 2.08 mmol, 1.5 equiv.), Pd(dba)$_2$ (0.0159 g, 0.028 mmol, 0.02 equiv.), SPhos (0.0284 g, 0.069 mmol, 0.05 equiv.) and KOAc (0.245 g, 2.49 mmol, 1.8 equiv.) were added into a microwave vial and the vial was subjected to three vacuum/Ar gas refill cycles. Anhydrous 1,4-dioxane (14 mL) was added and the mixture was heated at 80 °C oil bath for 24 h. After cooling to RT, the mixture was diluted with DEE, extracted with brine and water (3 × 200 mL) and the organic phase was dried over anhydrous $MgSO_4$. Solvent was removed under vacuum and the crude product was purified with column chromatography over silica gel, by gradually increasing the eluent polarity from hexane to 5% (v/v) DCM in hexane (before loading the crude product, the column was pre-treated by passing through a mixture of 3% (v/v) AcOH in hexane until the silica was saturated with AcOH, then excess AcOH was washed out by passing hexane through the column). Solvent was removed and the solids were sonicated first in MeOH (3 × 5 min) and then in hexane (3 × 5 min). The solids were collected each time by centrifuging and the solvent was removed by pipetting. Finally, the solids were precipitated from hexane/MeOH 1:1 (v/v) mixture, solvent was removed and the solids were dried in vacuum. The target compound was collected as white solid (0.787 g, 70%). An upscaled reaction yielded 1.705 g (61%). ^{1}H NMR (400 MHz, $CDCl_3$) δ 7.77 (d, J = 1.2 Hz, 1H), 7.64 (d, J = 1.3 Hz, 1H), 7.14 (dd, J = 11.6, 1.8 Hz, 2H), 7.01 (dd, J = 7.9, 1.8 Hz, 2H), 6.96 (s, 1H), 6.93 (s, 4H), 2.32 (s, 6H), 2.04 (dd, J = 6.9, 3.0 Hz, 12H), 1.34 (d, J = 5.2 Hz, 12H). ^{13}C NMR (100 MHz, $CDCl_3$) δ 142.14, 142.12, 139.06, 137.67, 137.65, 137.57, 137.51, 137.50, 137.02, 136.88, 136.85, 136.23, 136.20, 136.19, 135.92, 135.83, 135.77, 134.36, 134.22, 134.08, 131.17, 131.06, 129.40, 129.31, 128.31, 128.29, 128.27, 84.57, 51.02, 25.16, 24.88, 21.19, 20.72, 20.68, 20.66. TOF-MS-ASAP$^+$ Calcd. for

$[C_{43}H_{41}BCl_6O_2]^+$: 810.1331. Found: *m/z* = 810.1335. EA Calcd. for $C_{43}H_{41}BCl_6O_2$: C, 63.50; H, 5.08; B, 1.33; Cl, 26.15; O, 3.93. Found: C, 63.36; H, 4.88; N, 0.00.

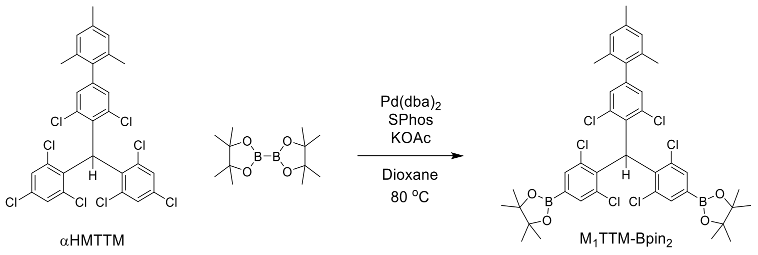

2,2'-(((3,5-Dichloro-2',4',6'-trimethyl-[1,1'-biphenyl]-4-yl)methylene)bis(3,5-dichloro-4,1-phenylene))bis(4,4,5,5-tetramethyl-1,3,2-dioxaborolane) (M_1TTM-Bpin$_2$). αHM_1TTM (0.600 g, 0.94 mmol, 1 equiv.), bis(pinacolato)diboron (0.716 g, 2.82 mmol, 3 equiv.), $Pd(dba)_2$ (0.0108 g, 0.019 mmol, 0.02 equiv.), SPhos (0.0193 g, 0.047 mmol, 0.05 equiv.) and KOAc (0.332 g, 3.39 mmol, 3.6 equiv.) were added into a microwave vial and the vial was subjected to three vacuum/Ar gas refill cycles. Anhydrous 1,4-dioxane (9 mL) was added and the mixture was heated at 80 °C oil bath for 24 h. After cooling to RT, the mixture was diluted with DEE, extracted with brine and water (3 × 200 mL) and the organic phase was dried over anhydrous $MgSO_4$. Solvent was removed under vacuum and the crude product was purified with column chromatography over silica gel, by gradually increasing the eluent polarity from hexane to 50% (v/v) DCM in hexane (before loading the crude product, the column was pre-treated by passing through a mixture of 3% (v/v) AcOH in hexane until the silica was saturated with AcOH, then excess AcOH was washed out by passing hexane through the column). Solvent was removed and the solids were sonicated first in MeOH (3 × 5 min) and then in hexane (3 × 5 min). The solids were collected each time by centrifuging and the solvent was removed by pipetting. Finally, the solids were precipitated from hexane/MeOH 1:1 (v/v) mixture, solvent was removed and the solids were dried in vacuum. The target compound was collected as white solid (0.540 g, 70%). An upscaled reaction yielded 2.038 g (63%). ^{1}H NMR (400 MHz, $CDCl_3$) δ 7.74 (dd, *J* = 10.7, 1.3 Hz, 2H), 7.61 (dd, *J* = 9.7, 1.3 Hz, 2H), 7.10 (d, *J* = 1.8 Hz, 1H), 6.98 (d, *J* = 1.7 Hz, 1H), 6.92 (d, *J* = 2.1 Hz, 3H), 2.31 (s, 3H), 2.02 (d, *J* = 3.4 Hz, 6H), 1.34 (d, *J* = 3.4 Hz, 24H). ^{13}C NMR (100 MHz, $CDCl_3$) δ 142.13, 138.84, 137.75, 137.57, 137.48, 136.93, 136.82, 136.76, 136.22, 136.17,

136.00, 135.86, 135.78, 134.30, 134.22, 134.01, 131.01, 129.28, 128.29, 128.26, 84.56, 51.37, 25.11, 24.95, 24.93, 21.18, 20.65. TOF-MS-ASAP$^+$ Calcd. for $[C_{40}H_{41}B_2Cl_6O_4]^+$: 817.1322. Found: *m/z* = 817.1345. EA Calcd. for $C_{40}H_{42}B_2Cl_6O_4$: C, 58.51; H, 5.16; B, 2.63; Cl, 25.90; O, 7.79. Found: C, 58.41; H, 5.00; N, 0.00.

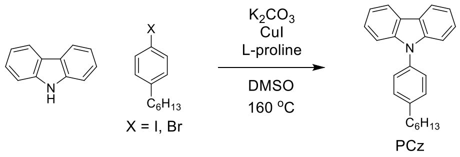

9-(4-Hexylphenyl)-9*H*-carbazole (PCz). Following modified literature procedure,[10] 9*H*-carbazole (3.000 g, 17.94 mmol, 1 equiv.), CuI (0.342 g, 1.79 mmol, 0.1 equiv.), L-proline (0.207 g, 1.79 mmol, 0.1 equiv.) and K_2CO_3 (4.959 g, 35.88 mmol, 2 equiv.) were added into an oven dried 50 mL two-neck round-bottom flask. The solids were dried under vacuum for 1 h and the flask was subjected to three vacuum/Ar gas refill cycles. Anhydrous DMSO (30 mL) and 1-hexyl-4-iodobenzene (7.755 g, 26.91 mmol, 1.5 equiv.) were added and the mixture was bubbled with Ar gas for 30 min, then refluxed at 160 °C for 16 h. After cooling to RT, the mixture was diluted with hexane and poured over water. The aqueous phase was acidified with 10% HCl solution. The organic phase was extracted with water (3 × 200 mL) and then dried over anhydrous $MgSO_4$. Solvent was removed under vacuum and the crude product was purified with column chromatography over silica gel, using hexane as the eluent. After removing the solvent in vacuum, the target compound was collected as colorless oil which slowly solidified to give white solid (5.60 g, 95%). Same reaction but using 1-hexyl-4-bromobenzene gave the product at lower yield of 41%. ^{1}H NMR (400 MHz, $CDCl_3$) δ 8.15 (d, *J* = 7.7 Hz, 2H), 7.46 (d, *J* = 8.0 Hz, 2H), 7.40 (d, *J* = 4.8 Hz, 6H), 7.32–7.24 (m, 2H), 2.74 (t, *J* = 7.8 Hz, 2H), 1.79–1.67 (m, 2H), 1.49–1.32 (m, 6H), 0.93 (t, *J* = 6.8 Hz, 3H). ^{13}C NMR (100 MHz, $CDCl_3$) δ 142.52, 141.20, 135.27, 129.87, 127.07, 125.95, 123.38, 120.37, 119.84, 109.97, 35.86, 31.90, 31.59, 29.22, 22.79, 14.27. TOF-MS-ASAP$^+$ Calcd. for $[C_{24}H_{26}N]^+$: 328.2065. Found: *m/z* = 328.2055. EA Calcd. for $C_{24}H_{25}N$: C, 88.03; H, 7.70; N, 4.28. Found: C, 87.60; H, 7.62; N, 4.32.

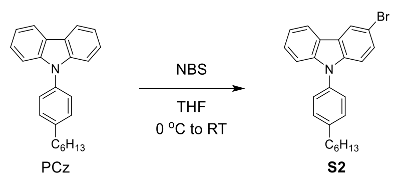

3-Bromo-9-(4-hexylphenyl)-9*H*-carbazole (S2). PCz (0.500 g, 1.53 mmol, 1 equiv.) was added into a 100 mL round-bottom flask and the flask was subjected to three vacuum/Ar gas refill cycles. Anhydrous THF (50 mL) was added to dissolve all starting material. The mixture was covered from light and cooled to 0 °C in an ice bath. *N*-Bromosuccinimide (0.272 g, 1.53 mmol, 1 equiv.) was dissolved in anhydrous THF (10 mL) and added to the reaction mixture drowise during 30 min in dark. Then, the mixture was allowed to warm slowly to RT. After 36 h, the reaction was quenched by adding water. The mixture was diluted with DEE, extracted with brine and water (3 × 200 mL) and and the organic phase was dried over anhydrous $MgSO_4$. Solvent was removed under vacuum and the crude product was purified with column chromatography over silica gel, using hexane as the eluent. After removing the solvent in vacuum, the target compound was collected as colorless oil (0.538 g, 87%). ^{1}H NMR (400 MHz, $CDCl_3$) δ 8.25 (d, J = 2.0 Hz, 1H), 8.09 (dt, J = 7.7, 1.0 Hz, 1H), 7.47 (dd, J = 8.6, 2.0 Hz, 1H), 7.43–7.38 (m, 6H), 7.30–7.24 (m, 2H), 2.73 (t, J = 7.8 Hz, 2H), 1.78–1.67 (m, 2H), 1.46–1.33 (m, 6H), 0.93 (t, J = 7.2 Hz, 3H). ^{13}C NMR (100 MHz, $CDCl_3$) δ 142.92, 141.53, 139.86, 134.80, 130.01, 128.63, 126.98, 126.70, 125.11, 123.11, 122.30, 120.56, 120.27, 112.61, 111.48, 110.22, 35.85, 31.89, 31.57, 29.21, 22.78, 14.27. TOF-MS-ASAP$^+$ Calcd. for $[C_{24}H_{25}BrN]^+$: 406.1170. Found: *m/z* = 406.1163. EA Calcd. for $C_{24}H_{24}BrN$: C, 70.94; H, 5.95; Br, 19.66; N, 3.45. Found: C, 70.07; H, 5.89; N, 3.41.

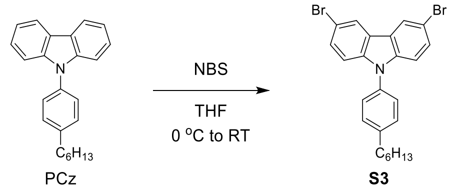

3,6-Dibromo-9-(4-hexylphenyl)-9*H*-carbazole (S3). Following the procedure described above for 3-bromo-9-(4-hexylphenyl)-9*H*-carbazole, PCz (2.000 g, 6.11 mmol, 1 equiv.) was added into a 250 mL round-bottom flask and the flask was subjected to three vacuum/Ar gas refill cycles. Anhydrous THF

(200 mL) was added to dissolve all starting material. The mixture was covered from light and cooled to 0 °C in an ice bath. *N*-Bromosuccinimide (2.174 g, 12.21 mmol, 2 equiv.) was dissolved in anhydrous THF (40 mL) and added to the reaction mixture slowly during 30 min in dark. Then, the mixture was allowed to warm slowly to RT. After 24 h, the reaction was quenched by adding water. The mixture was diluted with DEE, extracted with brine and water (3 × 200 mL) and and the organic phase was dried over anhydrous $MgSO_4$. Solvent was removed under vacuum and the crude product was purified with column chromatography over silica gel, by gradually increasing the eluent polarity from hexane to 5% (v/v) DCM in hexane. Solvent was removed and the product was recrystallised from hexane/DCM mixture by slow evaporation of DCM. After removing the solvent in vacuum, the target compound was collected as white solid (2.059 g, 69%). ^{1}H NMR (400 MHz, $CDCl_3$) δ 8.19 (d, J = 1.9 Hz, 2H), 7.49 (dd, J = 8.8, 1.9 Hz, 2H), 7.43–7.34 (m, 4H), 7.24 (d, J = 8.7 Hz, 2H), 2.73 (t, J = 7.8 Hz, 2H), 1.76–1.66 (m, 2H), 1.47–1.34 (m, 6H), 0.92 (t, J = 7.2 Hz, 3H). ^{13}C NMR (100 MHz, $CDCl_3$) δ 143.31, 140.17, 134.33, 130.13, 129.42, 126.89, 123.95, 123.28, 113.01, 111.73, 35.85, 31.87, 31.55, 29.20, 22.77, 14.26. TOF-MS-ASAP$^+$ Calcd. for $[C_{24}H_{24}Br_2N]^+$: 484.0276. Found: *m/z* = 484.0276. EA Calcd. for $C_{24}H_{23}Br_2N$: C, 59.40; H, 4.78; Br, 32.93; N, 2.89. Found: C, 59.13; H, 4.71; N, 2.95.

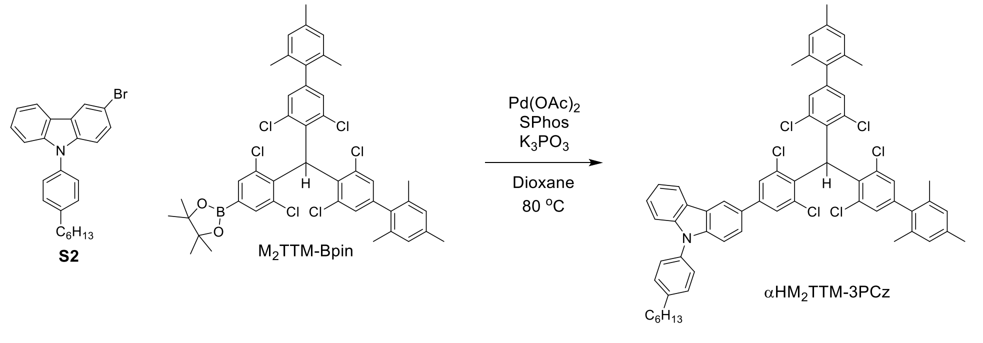

3-(4-(Bis(3,5-dichloro-2',4',6'-trimethyl-[1,1'-biphenyl]-4-yl)methyl)-3,5-dichlorophenyl)-9-(4-hexylphenyl)-9*H*-carbazole (αHM$_2$TTM-3PCz). S2 (0.050 g, 0.12 mmol, 1 equiv.), M_2TTM-Bpin (0.120 g, 0.15 mmol, 1.2 equiv.), $Pd(OAc)_2$ (0.0008 g, 0.0037 mmol, 0.03 equiv.), SPhos (0.0038 g, 0.0092 mmol, 0.075 equiv.) and K_3PO_4 (0.038 g, 0.18 mmol, 1.4 equiv.) were added into a microwave vial and the vial was subjected to three vacuum/Ar gas refill cycles. Anhydrous 1,4-dioxane (1 mL)

was added and the mixture was heated at 80 °C oil bath for 24 h. After cooling to RT, the mixture was diluted with hexane, extracted with water (3 × 200 mL) and the organic phase was dried over anhydrous $MgSO_4$. Solvent was removed under vacuum and the crude product was purified with column chromatography over silica gel, by gradually increasing the eluent polarity from hexane to 10% (v/v) DCM in hexane. Solvent was removed and the solids were sonicated in MeOH (3 × 5 min) and washed with hexane/MeOH 1:1 (v/v) mixture. The solids were collected each time by centrifuging and the solvent was removed by pipetting. Finally, the solids were dried in vacuum and the target compound was collected as white solid (0.081 g, 65%). An upscaled reaction yielded 0.174 g (70%). ^{1}H NMR (400 MHz, $CDCl_3$) δ 8.37 (d, J = 1.8 Hz, 1H), 8.19 (d, J = 7.8 Hz, 1H), 7.75 (d, J = 2.0 Hz, 1H), 7.64 (dd, J = 8.6, 1.8 Hz, 1H), 7.61 (d, J = 2.0 Hz, 1H), 7.51–7.39 (m, 7H), 7.32 (ddd, J = 8.0, 5.9, 2.2 Hz, 1H), 7.19 (dd, J = 2.7, 1.8 Hz, 2H), 7.06 (t, J = 1.6 Hz, 2H), 7.03 (s, 1H), 6.95 (s, 4H), 2.75 (t, J = 7.8 Hz, 2H), 2.33 (s, 6H), 2.08 (s, 12H), 1.80–1.68 (m, 2H), 1.50–1.34 (m, 6H), 0.94 (t, J = 7.1 Hz, 3H). ^{13}C NMR (100 MHz, $CDCl_3$) δ 142.67, 142.47, 141.96, 141.60, 141.02, 137.75, 137.64, 137.52, 137.35, 137.14, 136.84, 136.08, 135.76, 135.72, 135.69, 134.84, 134.27, 134.17, 133.74, 131.05, 129.85, 129.80, 129.31, 129.22, 128.55, 128.15, 128.14, 126.86, 126.80, 126.29, 124.87, 123.85, 123.18, 120.37, 120.14, 118.72, 110.29, 110.12, 50.52, 35.73, 31.75, 31.44, 29.08, 22.64, 21.05, 20.60, 20.58, 20.56, 14.13. TOF-MS-ASAP$^+$ Calcd. for $[C_{61}H_{54}Cl_6N]^+$: 1010.2387. Found: m/z = 1010.2358. EA Calcd. for $C_{61}H_{53}Cl_6N$: C, 72.34; H, 5.27; Cl, 21.00; N, 1.38. Found: C, 72.17; H, 5.18; N, 1.40.

General procedure for the conversion of αH precursors to π-radicals. The αH precursor was added into a round-bottom flask and the flask was subjected to three vacuum/Ar gas refill cycles. Anhydrous THF was added to dissolve all starting material followed by addition of anhydrous DMSO in 1:3 (v/v) THF/DMSO ratio. The mixture was bubbled with Ar gas for 15 min and covered carefully from light. In the darkness, 40% Bu_4NOH (aq, bubbled with Ar gas for 15 min prior to use) was added and the mixture was stirred at RT until deprotonation was complete, as detailed below (for further discussion, see Supplementary Note 4). *p*-Chloranil was added and the mixture was stirred until complete oxidation to neutral radical. Under minimal amount of ambient light, the mixture was diluted with

hexane, extracted with water (3 × 200 mL) and the organic phase was dried over anhydrous $MgSO_4$. Solvent was removed under vacuum and the crude product was purified with column chromatography over silica gel, as detailed below. Solvent was removed and the solids were sonicated in MeOH for 5 min. The solids were collected by centrifuging and MeOH was removed by pipetting. Finally, the solids were dried in vacuum. All radicals were stable under ambient air in both solution and solid state, but when stored for longer times the materials were kept under inert gas.

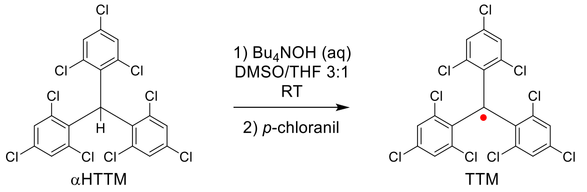

Tris(2,4,6-trichlorophenyl)methyl radical (TTM). Following the above general procedure, αHTTM (2.079 g, 3.75 mmol, 1 equiv.) was dissolved in anhydrous THF (105 mL) followed by addition of anhydrous DMSO (310 mL). 40% Bu_4NOH (aq) (5.0 mL, 7.50 mmol, 2 equiv.) was added and the mixture was stirred for 4 h. Following the addition of base, the initially colorless reaction mixture quickly acquired claret color. *p*-Chloranil (2.305 g, 9.38 mmol, 2.5 equiv.) was added and the mixture was stirred another 1 h, quickly changing color to dark red. Column chromatography was carried out using hexane as the eluent. The target compound was collected as red solid (1.895 g, 91%). TOF-MS-ASAP$^+$ Calcd. for $[C_{19}H_7Cl_9]^+$: 549.7744. Found: m/z = 549.7730. EA Calcd. for $C_{19}H_6Cl_9^{\bullet}$: C, 41.24; H, 1.09; Cl, 57.66. Found: C, 41.08; H, 0.98; N, 0.00.

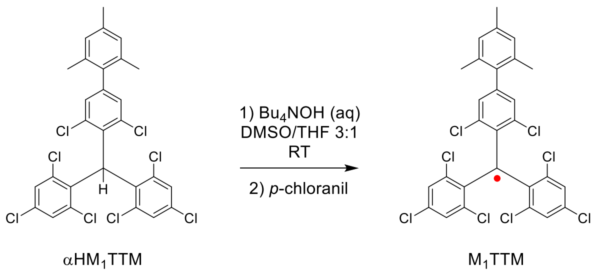

4'-(Bis(2,4,6-trichlorophenyl)methyl)-3',5'-dichloro-2,4,6-trimethyl-1,1'-biphenyl radical (M_1TTM). Following the above general procedure, αHM_1TTM (0.300 g, 0.47 mmol, 1 equiv.) was

dissolved in anhydrous THF (15 mL) followed by addition of anhydrous DMSO (45 mL). 40% Bu_4NOH (aq) (0.63 mL, 0.94 mmol, 2 equiv.) was added and the mixture was stirred for 5 h. Following the addition of base, the initially colorless reaction mixture quickly acquired claret color. *p*-Chloranil (0.289 g, 1.18 mmol, 2.5 equiv.) was added and the mixture was stirred another 1 h, quickly changing color to dark red. Column chromatography was carried out using hexane as the eluent. The target compound was collected as red solid (0.246 g, 82%). FTMS-ESI$^+$ Calcd. for $[C_{28}H_{17}Cl_8]^+$: 632.8833. Found: *m/z* = 632.8807. EA Calcd. for $C_{28}H_{17}Cl_8^{\bullet}$: C, 52.79; H, 2.69; Cl, 44.52. Found: C, 52.07; H, 2.61; N, 0.00.

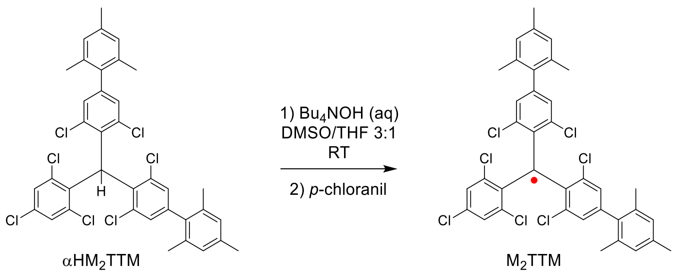

4',4'''-((2,4,6-Trichlorophenyl)methyl)bis(3',5'-dichloro-2,4,6-trimethyl-1,1'-biphenyl) radical (M_2TTM). Following the above general procedure, αHM_2TTM (0.300 g, 0.42 mmol, 1 equiv.) was dissolved in anhydrous THF (15 mL) followed by addition of anhydrous DMSO (45 mL). 40% Bu_4NOH (aq) (0.55 mL, 0.83 mmol, 2 equiv.) was added and the mixture was stirred for 5 h. Following the addition of base, the initially colorless reaction mixture quickly acquired claret color. *p*-Chloranil (0.255 g, 1.04 mmol, 2.5 equiv.) was added and the mixture was stirred another 1 h, quickly changing color to dark red. Column chromatography was carried out using hexane as the eluent. The target compound was collected as red solid (0.277 g, 92%). FTMS-ESI$^+$ Calcd. for $[C_{37}H_{28}Cl_7]^+$: 717.0005. Found: *m/z* = 716.9996. EA Calcd. for $C_{37}H_{28}Cl_7^{\bullet}$: C, 61.66; H, 3.92; Cl, 34.43. Found: C, 61.64; H, 3.84; N, 0.00.

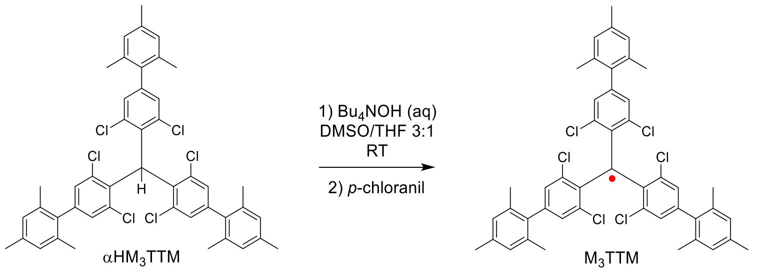

Tris(3,5-dichloro-2',4',6'-trimethyl-[1,1'-biphenyl]-4-yl)methyl radical (M3TTM). Following the above general procedure, αHM3TTM (0.600 g, 0.74 mmol, 1 equiv.) was dissolved in anhydrous THF (30 mL) followed by addition of anhydrous DMSO (90 mL). 40% Bu_4NOH (aq) (1.00 mL, 1.49 mmol, 2 equiv.) was added and the mixture was stirred for 5 h. Following the addition of base, the initially colorless reaction mixture quickly acquired claret color. *p*-Chloranil (0.458 g, 1.86 mmol, 2.5 equiv.) was added and the mixture was stirred another 1 h, quickly changing color to dark red. Column chromatography was carried out by gradually increasing the eluent polarity from hexane to 5% (v/v) DCM in hexane. The target compound was collected as red solid (0.470 g, 78%). FTMS-ESI$^+$ Calcd. for $[C_{46}H_{39}Cl_6]^+$: 801.1177. Found: *m/z* = 801.1154. EA Calcd. for $C_{46}H_{39}Cl_6^{\bullet}$: C, 68.68; H, 4.89; Cl, 26.44. Found: C, 68.39; H, 4.79; N, 0.00.

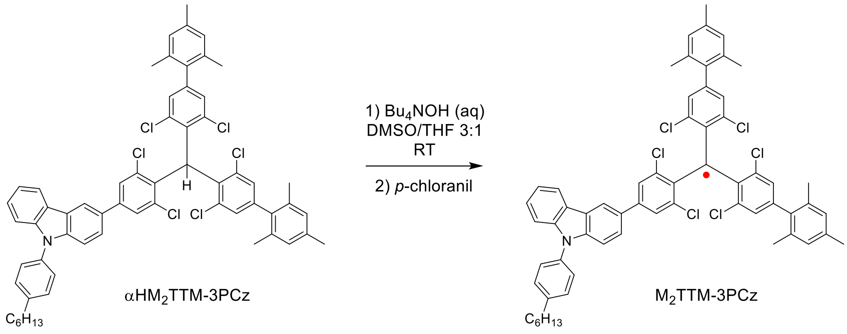

3-(4-(Bis(3,5-dichloro-2',4',6'-trimethyl-[1,1'-biphenyl]-4-yl)methyl)-3,5-dichlorophenyl)-9-(4-hexylphenyl)-9*H*-carbazolyl radical (M2TTM-3PCz). Following the above general procedure, αHM2TTM-3PCz (0.0400 g, 0.039 mmol, 1 equiv.) was dissolved in anhydrous THF (2 mL) followed by addition of anhydrous DMSO (6 mL). 40% Bu_4NOH (aq) (0.05 mL, 0.079 mmol, 2 equiv.) was added and the mixture was stirred for 6 h. Following the addition of base, the initially colorless reaction mixture quickly acquired dark purple color. *p*-Chloranil (0.0243 g, 0.099 mmol, 2.5 equiv.) was added and the mixture was stirred another 6 h, quickly changing color to dark green. Column chromatography

was carried out by gradually increasing the eluent polarity from hexane to 5% (v/v) DCM in hexane. The target compound was collected as dark green solid (0.031 g, 78%). An upscaled reaction yielded 0.148 g (93%). TOF-MS-ASAP$^+$ Calcd. for $[C_{61}H_{53}Cl_6N]^+$: 1009.2309. Found: *m/z* = 1009.2261. EA Calcd. for $C_{61}H_{52}Cl_6N^{\bullet}$: C, 72.41; H, 5.18; Cl, 21.02; N, 1.38. Found: C, 72.35; H, 5.21; N, 1.37.

General procedure for the synthesis of αH polymer precursors. Equimolar amounts of dibromo-functionalized monomer and bis(pinacol) ester derivative of αH precursor monomer, $Pd(OAc)_2$ as the palladium source, SPhos as the phosphine ligand and K_3PO_4 as the base were added into a microwave vial and the vial was subjected to three vacuum/Ar gas refill cycles. Anhydrous 1,4-dioxane was added and the mixture was bubbled with Ar gas for 15 min. The mixture was stirred vigorously at 80 °C oil bath for 72 h. After cooling to RT, the mixture was precipitated by dropping slowly into MeOH. The solids were collected by filtration through a Soxhlet thimble and washed with Soxhlet extraction using MeOH and acetone as the solvent (12 h each step). The polymer was finally collected with chloroform (6 h). The chloroform fraction was purified by passing through a short silica gel plug, then concentrated and precipitated again into MeOH. The solids were collected by filtration and dried in vacuum.

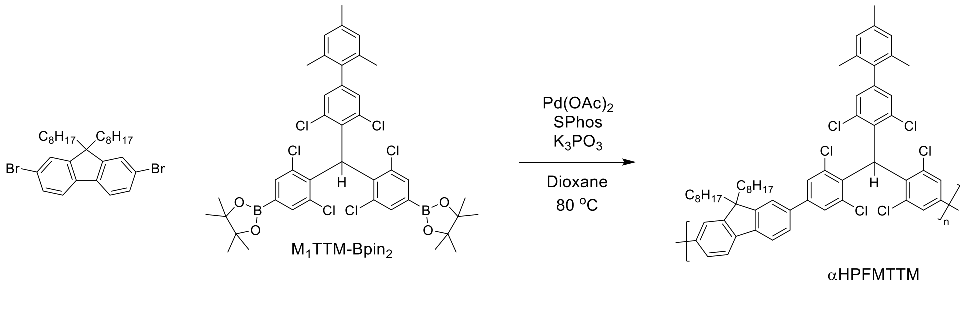

Poly[9,9-dioctyl-9*H*-fluorene-2,7-diyl-*alt*-4'-(bis(2,6-dichlorophenyl)methyl)-3',5'-dichloro-2,4,6-trimethyl-1,1'-biphenyl-4,4'-diyl] (αHPFMTTM). Following the above general procedure, 2,7-dibromo-9,9-dioctyl-9*H*-fluorene (0.0548 g, 0.100 mmol, 1 equiv.), M_1TTM-$Bpin_2$ (0.0821 g, 0.100 mmol, 1 equiv.), $Pd(OAc)_2$ (0.0002 g, 0.0010 mmol, 0.01 equiv.), SPhos (0.0008 g, 0.0020 mmol, 0.02 equiv.), K_3PO_4 (0.0849 g, 0.400 mmol, 4 equiv.) and anhydrous 1,4-dioxane (1 mL) were used. After purifications following the above described procedure, the polymer was collected as off-

white solid (0.065 g, 68%). ^{1}H NMR (400 MHz, $CDCl_3$) δ 7.80 (d, J = 7.6 Hz, 2H), 7.72 (d, J = 6.1 Hz, 2H), 7.65–7.54 (m, 6H), 7.19 (s, 1H), 7.07 (s, 1H), 7.03 (s, 1H), 6.95 (s, 2H), 2.33 (s, 3H), 2.13–1.99 (m, 10H), 1.20–1.03 (m, 20H), 0.79 (t, J = 7.2 Hz, 6H), 0.73–0.58 (m, 4H). GPC: M_n = 25.2 kg/mol, M_w = 50.7 kg/mol, PDI = 2.0, $\bar{X}_n$ = 26.2. EA Calcd. for $H(C_{57}H_{58}Cl_6)_{26}H$: C, 71.62; H, 6.12; Cl, 22.25. Found: C, 71.25; H, 6.07; N, 0.00.

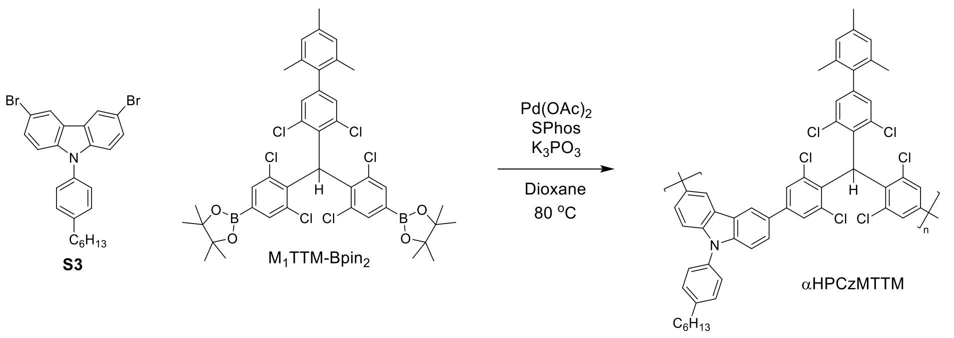

Poly[9-(4-hexylphenyl)-9*H*-carbazole-3,6-diyl-*alt*-4'-(bis(2,6-dichlorophenyl)methyl)-3',5'-dichloro-2,4,6-trimethyl-1,1'-biphenyl-4,4'-diyl] (αHPCzMTTM). Following the above general procedure, **S3** (0.0485 g, 0.100 mmol, 1 equiv.), M_1TTM-Bpin$_2$ (0.0821 g, 0.100 mmol, 1 equiv.), $Pd(OAc)_2$ (0.0002 g, 0.0010 mmol, 0.01 equiv.), SPhos (0.0008 g, 0.0020 mmol, 0.02 equiv.), K_3PO_4 (0.0849 g, 0.400 mmol, 4 equiv.) and anhydrous 1,4-dioxane (1 mL) were used. After purifications following the above described procedure, the polymer was collected as off-white solid (0.044 g, 49%). ^{1}H NMR (400 MHz, $CDCl_3$) δ 8.42 (s, 2H), 7.76 (s, 2H), 7.63 (t, J = 9.4 Hz, 4H), 7.51–7.40 (m, 6H), 7.15 (s, 1H), 7.02 (s, 2H), 6.91 (s, 2H), 2.79–2.67 (m, 2H), 2.29 (s, 3H), 2.04 (s, 6H), 1.78–1.67 (m, 2H), 1.45–1.32 (m, 6H), 0.92 (m, 3H). GPC: M_n = 6.8 kg/mol, M_w = 14.1 kg/mol, PDI = 2.1, $\bar{X}_n$ = 7.6. EA Calcd. for $H(C_{52}H_{41}Cl_6N)_8H$: C, 69.95; H, 4.66; Cl, 23.82; N, 1.57. Found: C, 69.85; H, 4.59; N, 1.57.

General procedure for the conversion of αH polymer precursors to polyradicals. The αH polymer precursor was added into a round-bottom flask and the flask was subjected to three vacuum/Ar gas refill cycles. Anhydrous THF was added to dissolve the polymer. Anhydrous DMSO was added slowly while vigorously stirring the mixture to avoid formation of large precipitates. The ratio of THF/DMSO

was 1:3 (v/v). The cloudy mixture was bubbled with Ar gas for 15 min and covered carefully from light. In the darkness, 40% Bu_4NOH (aq, bubbled with Ar gas for 15 min prior to use) was added slowly and the mixture was stirred vigorously at RT until no longer change was observed in the UV-Vis absorption spectrum (see Supplementary Fig. 19 and 20). At this stage the polymer was fully dissolved due to deprotonation to its polyanionic form. *p*-Chloranil was added and the polymer precipitated from the solution following complete oxidation to a polylradical. Under minimal amount of ambient light, the mixture was diluted with chloroform, poured over hexane and extracted with water (5 × 200 mL) in order to remove the excess base and *p*-chloranil. The organic phase was concentrated and the polymer was precipitated by dropping slowly into MeOH. The solids were collected by filtration through a Soxhlet thimble and washed with Soxhlet extraction under Ar gas using MeOH and acetone as the solvent (12 h each step). The polymer was finally collected with chloroform (6 h). The chloroform fraction was purified by passing through a short silica gel plug, then concentrated and precipitated again into MeOH. The solids were collected by filtration and dried in vacuum. The polyradicals were stable under ambient air in both solution and solid state, but when stored for longer times the polymers were kept under inert gas.

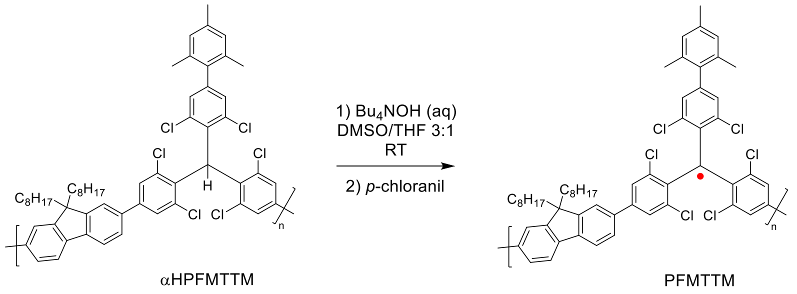

Poly[9,9-dioctyl-9*H*-fluorenyl-2,7-diyl-*alt*-4'-(bis(2,6-dichlorophenyl)methyl)-3',5'-dichloro-2,4,6-trimethyl-1,1'-biphenyl-4,4'-diyl] radical (PFMTTM). Following the above general procedure, αHPFMTTM (0.0400 g, 0.042 mmol, 1 repeating unit equiv.) was dissolved in anhydrous THF (4 mL) followed by addition of anhydrous DMSO (12 mL). 40% Bu_4NOH (aq) (in total 0.11 mL, 0.167 mmol, 4 equiv. per repeating unit) was added in three portions (2 equiv. at T0, 1 equiv. after 42 h and 1 equiv. after 66 h) and the mixture was stirred for 72 h until the deprotonation was complete

(Supplementary Fig. 19). Following the addition of base, the initially cloudy reaction mixture gradually dissolved and acquired dark blue color. *p*-Chloranil (0.0257 g, 0.104 mmol, 2.5 equiv. per repeating unit) was dissolved in 1 mL of anhydrous DMSO/THF mixture and added to the reaction mixture. The mixture was stirred another 12 h. During this time the polymer precipitated from the solution as dark green solid. After purifications following the above described procedure, the polymer was collected as dark green solid (0.027 g, 68%). GPC: M_n = 26.7 kg/mol, M_w = 55.3 kg/mol, PDI = 2.1, $\bar{X}_n$ = 27.9. EA Calcd. for $H(C_{57}H_{57}Cl_6^{\bullet})_{28}H$: C, 71.70; H, 6.03; Cl, 22.28. Found: C, 70.56; H, 6.07; N, 0.00.

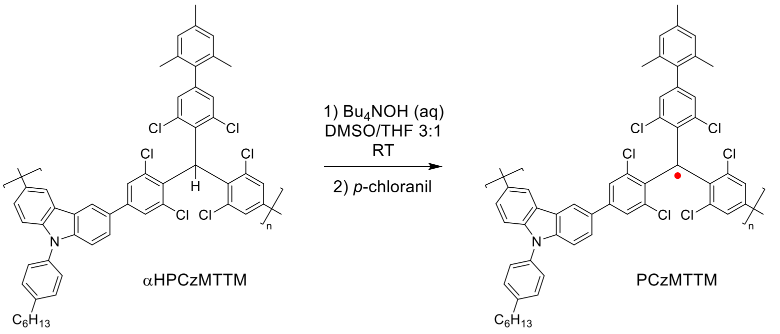

Poly[9-(4-hexylphenyl)-9*H*-carbazolyl-3,6-diyl-*alt*-4'-(bis(2,6-dichlorophenyl)methyl)-3',5'-dichloro-2,4,6-trimethyl-1,1'-biphenyl-4,4'-diyl] radical (PCzMTTM). Following the above general procedure, αHPCzMTTM (0.0300 g, 0.033 mmol, 1 repeating unit equiv.) was dissolved in anhydrous THF (3 mL) followed by addition of anhydrous DMSO (9 mL). 40% Bu_4NOH (aq) (in total 0.07 mL, 0.100 mmol, 3 equiv. per repeating unit) was added in two portions (2 equiv. at T0 and 1 equiv. after 42 h) and the mixture was stirred for 66 h until the deprotonation was complete (Supplementary Fig. 20). Following the addition of base, the initially cloudy reaction mixture gradually dissolved and acquired dark purple color. *p*-Chloranil (0.0206 g, 0.084 mmol, 2.5 equiv. per repeating unit) was dissolved in 1 mL of anhydrous DMSO/THF mixture and added to the reaction mixture. The mixture was stirred another 6 h. During this time the polymer precipitated from the solution as dark green solid. After purifications following the above described procedure, the polymer was collected as dark green solid (0.021 g, 70%). GPC: M_n = 8.4 kg/mol, M_w = 15.2 kg/mol, PDI = 1.8, $\bar{X}_n$ = 9.3. EA Calcd. for $H(C_{52}H_{40}Cl_6N^{\bullet})_9H$: C, 70.03; H, 4.55; Cl, 23.85; N, 1.57. Found: C, 68.86; H, 4.65; N, 1.65.

Supplementary Note 2: NMR Spectra

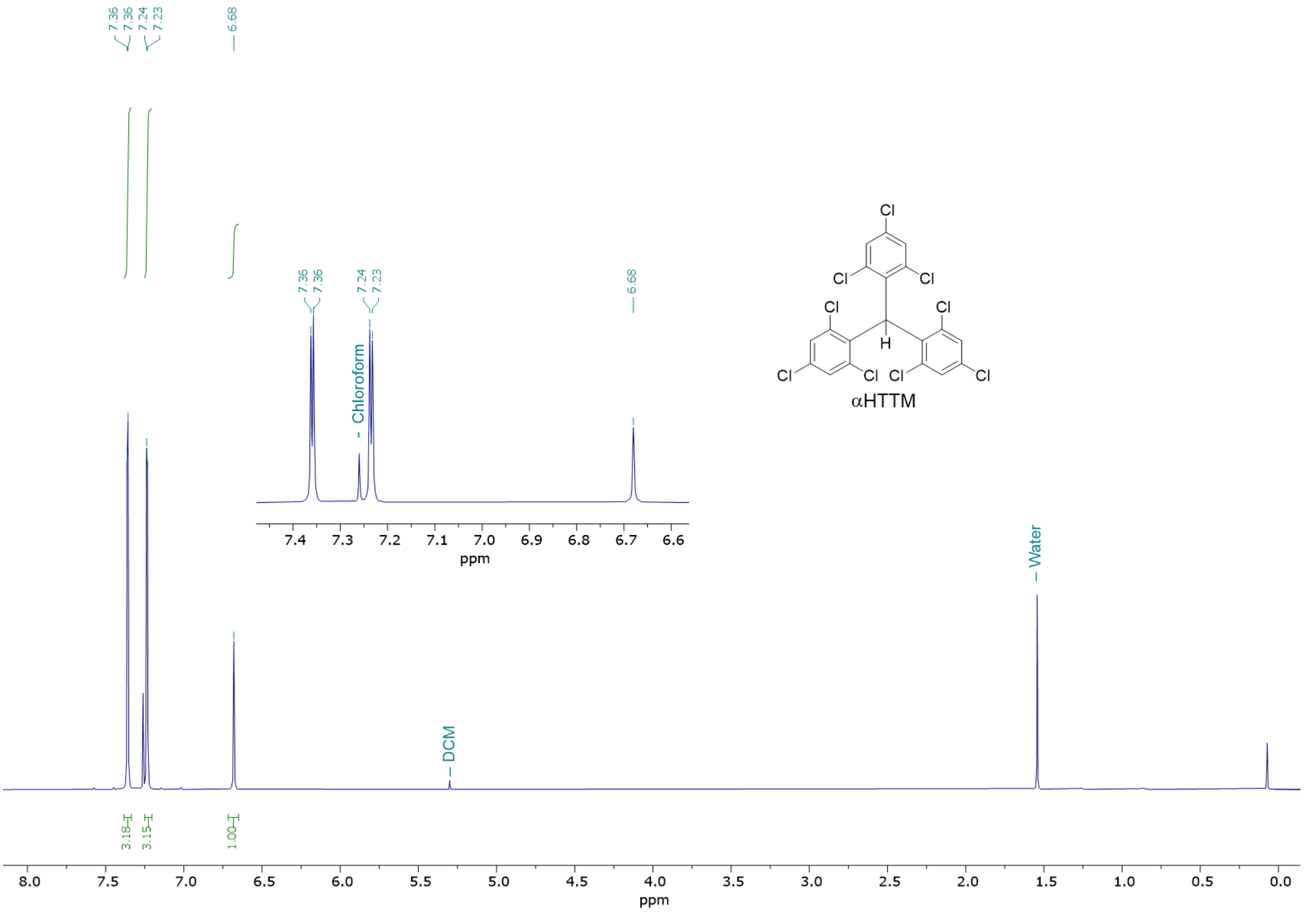

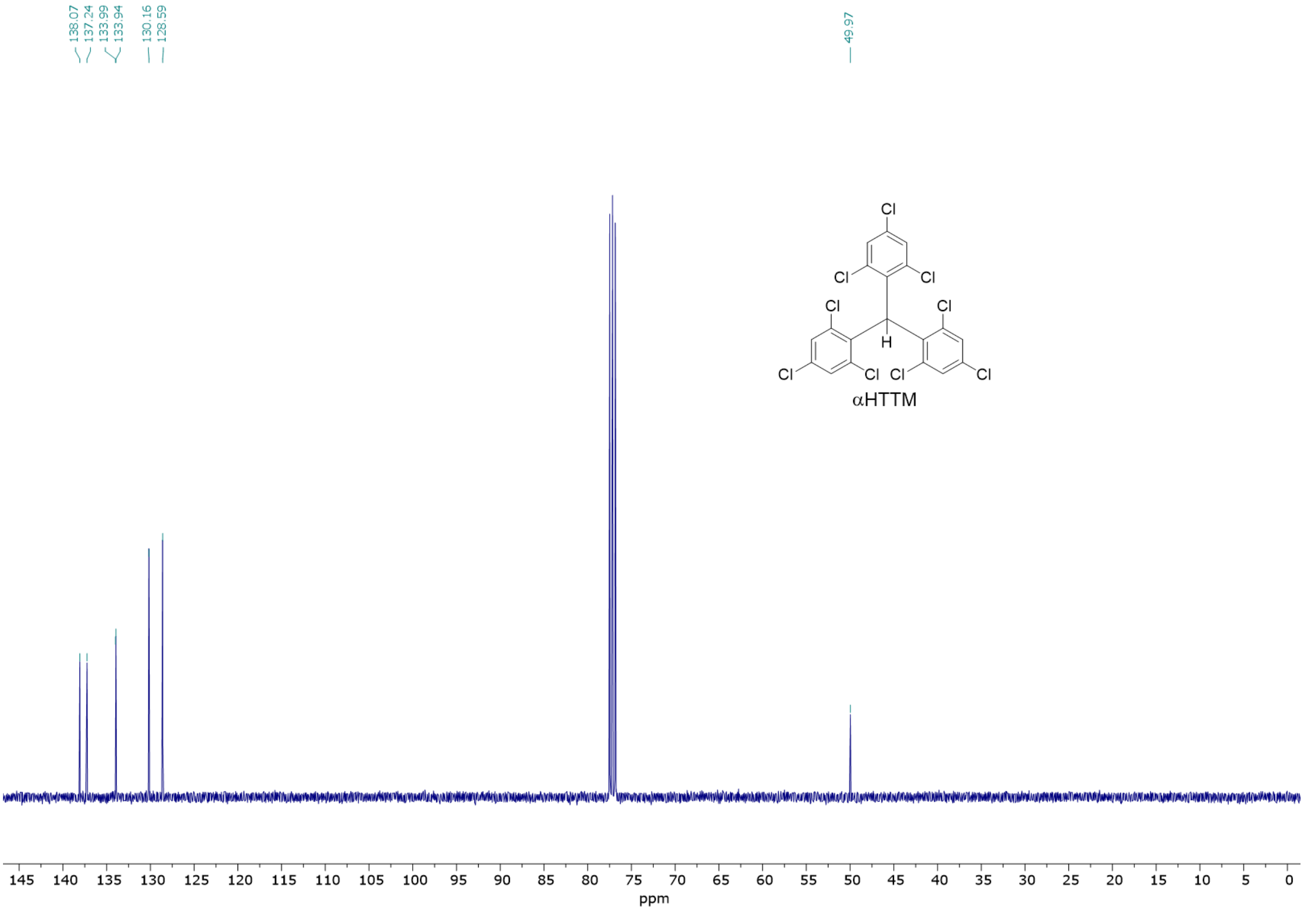

COSY

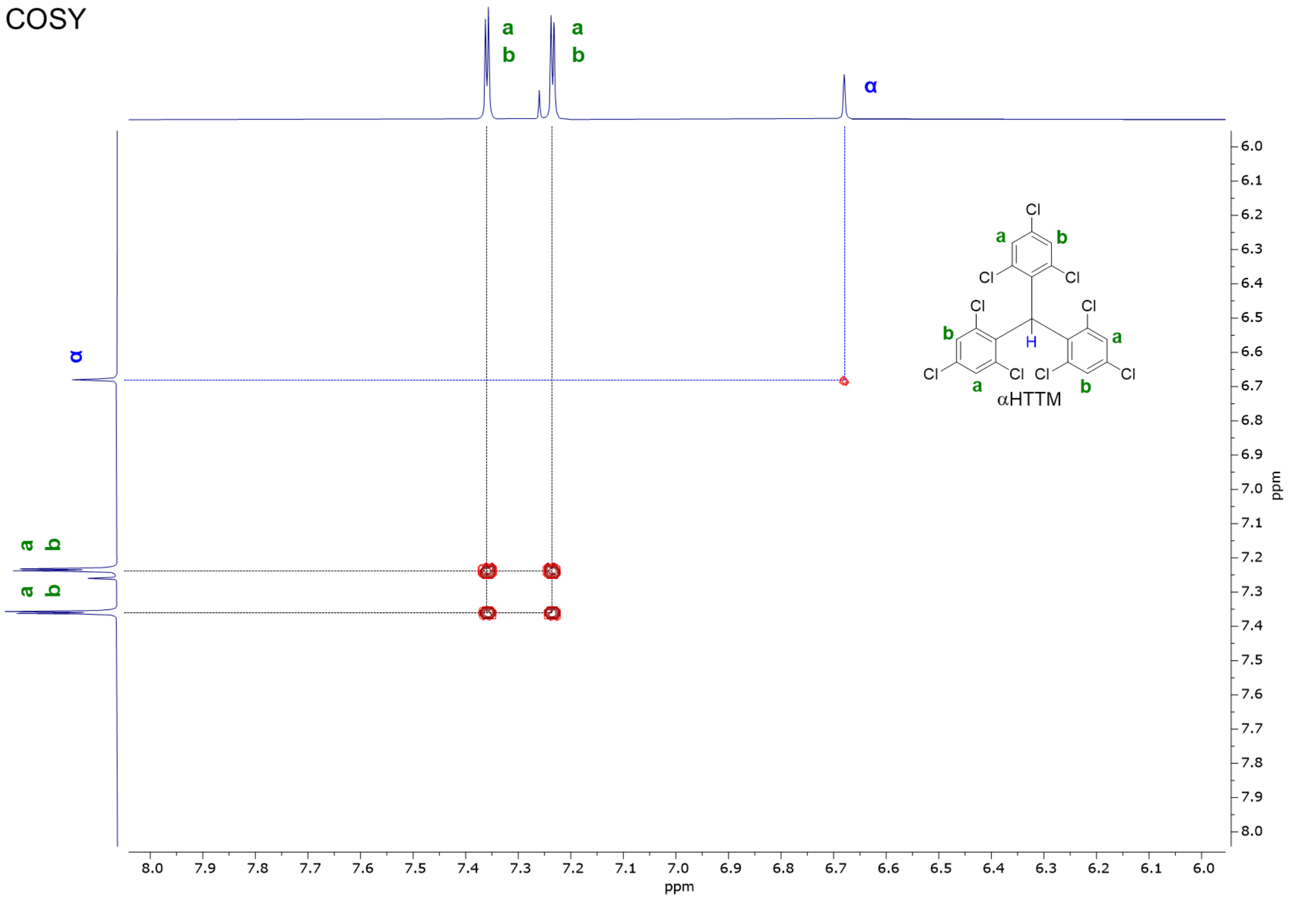

HSQC

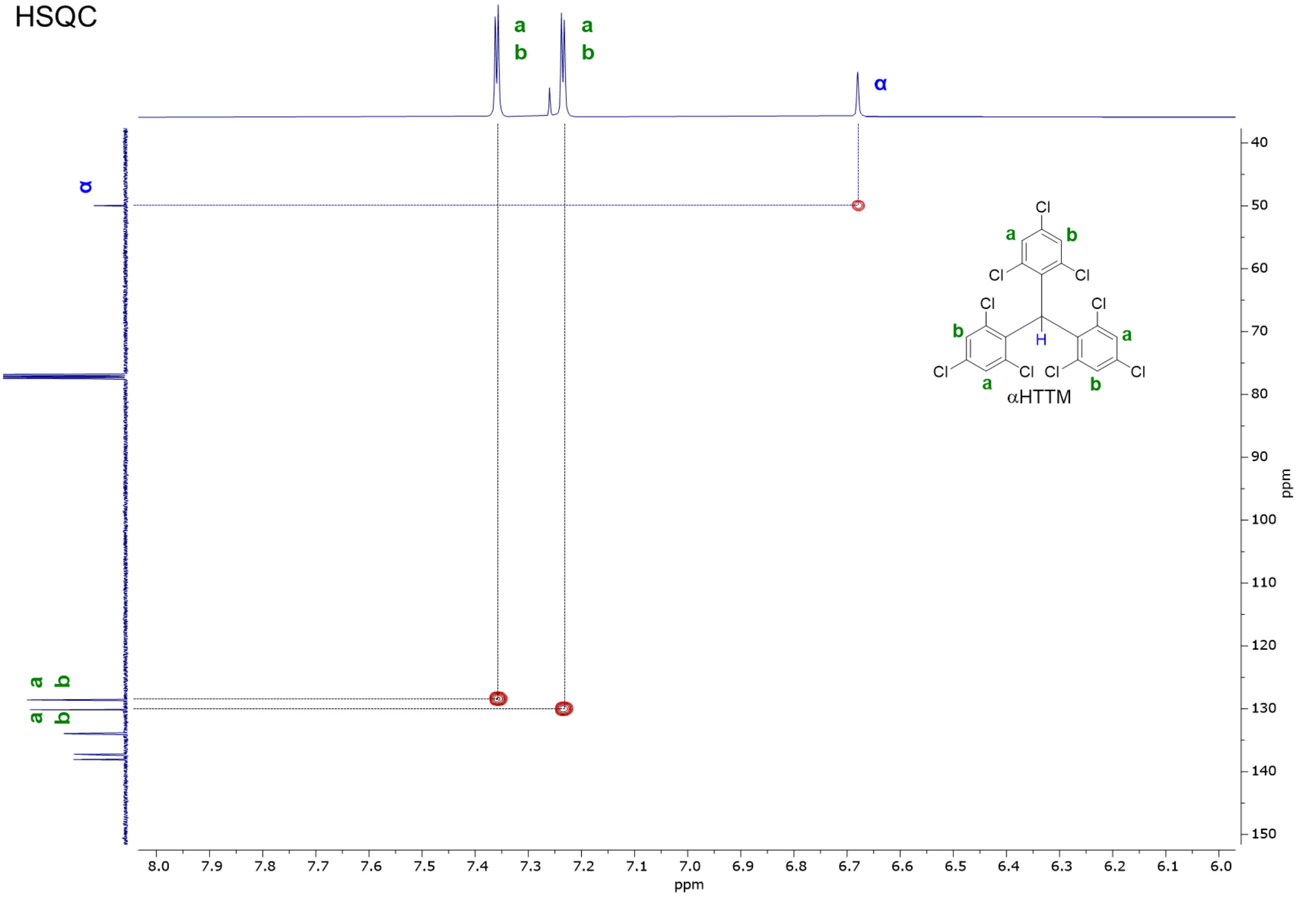

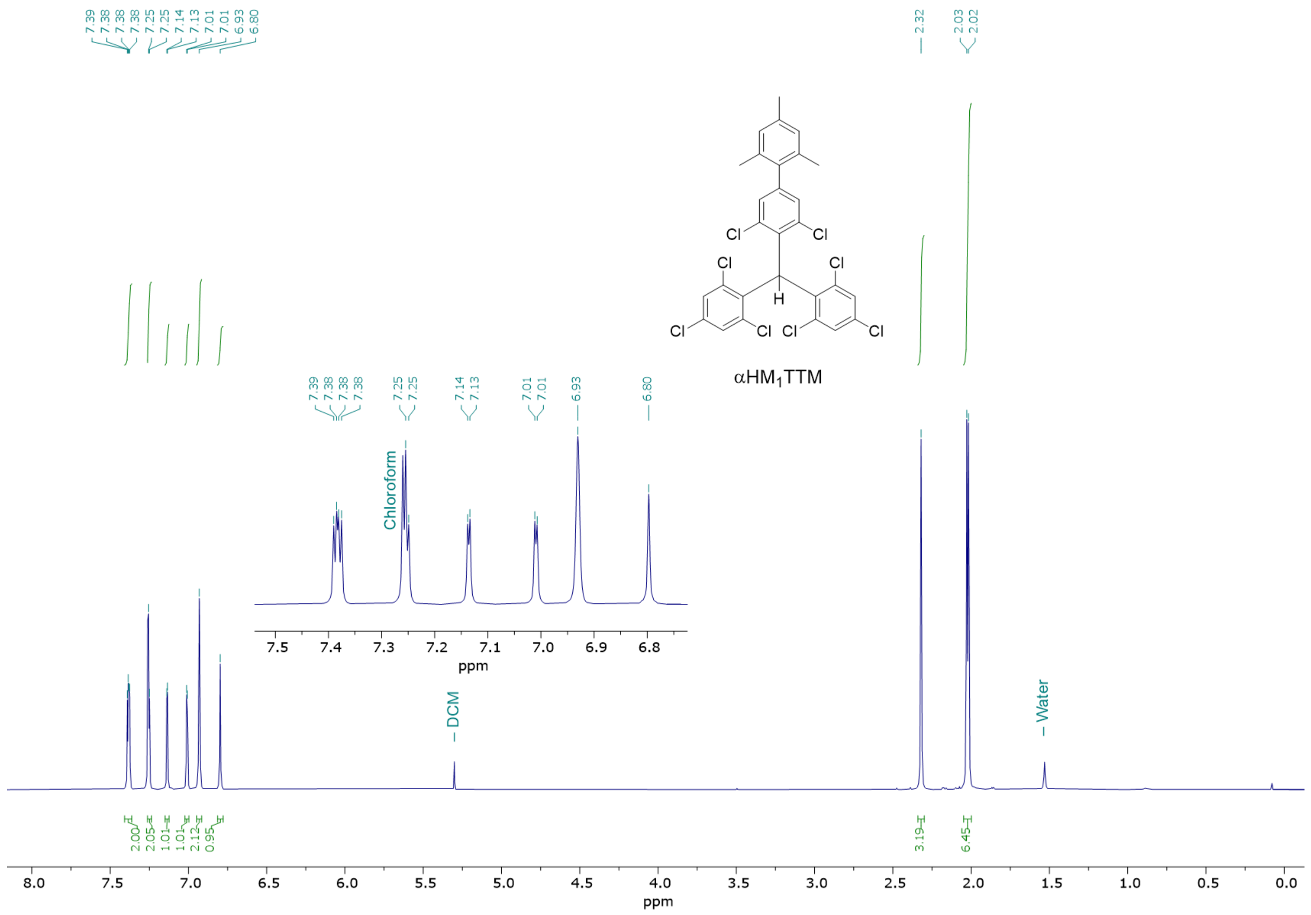
7.39 7.38 7.38 7.38 7.25 7.25 7.14 7.13 7.01 7.01 6.93 6.80
2.32 2.03 2.02
Cl
H
αHM1TTM
Chloroform
DCM
Water
2.00 2.05 1.01 1.01 2.12 0.95
3.19 6.45
ppm

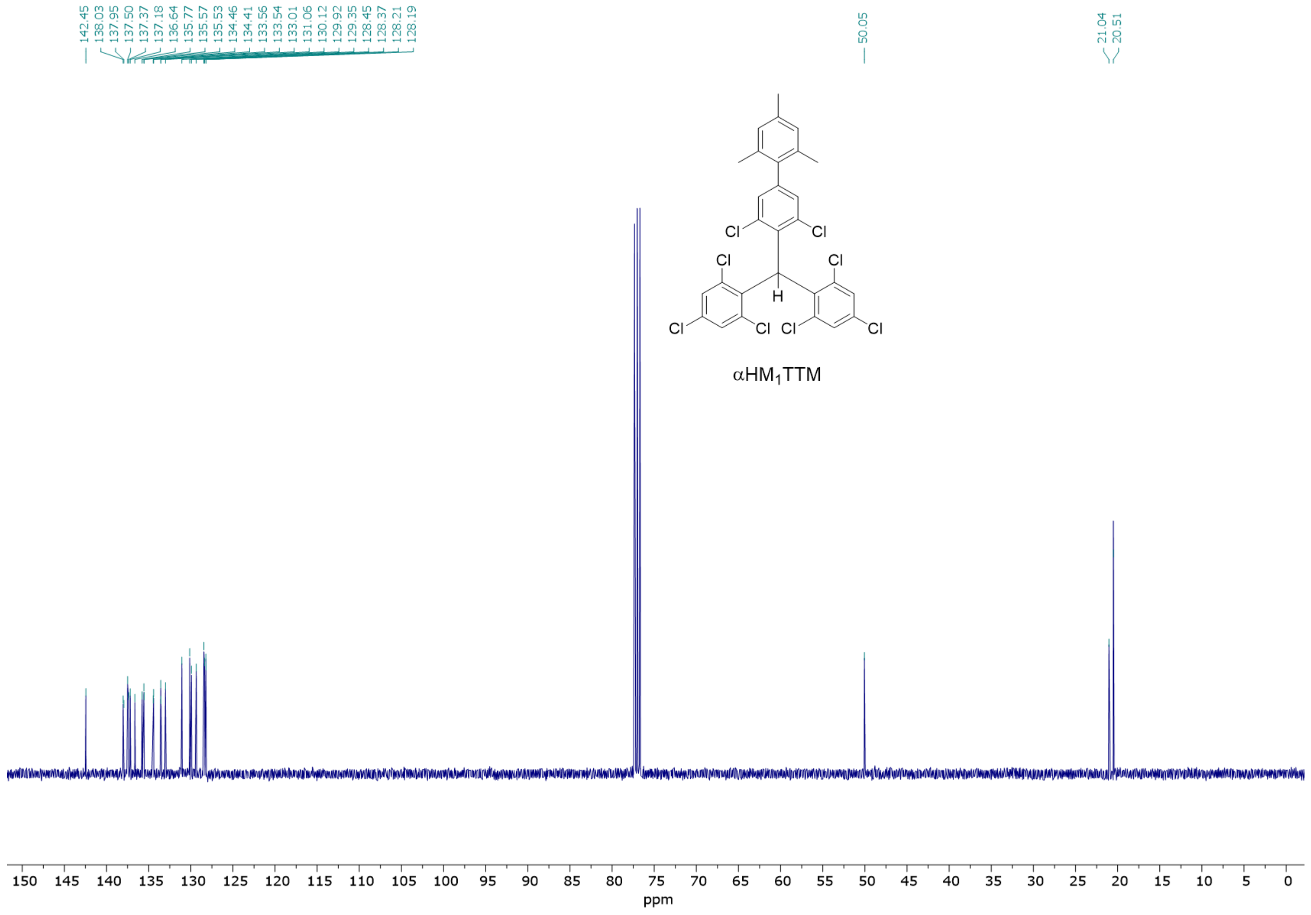
142.45 138.03 137.95 137.50 137.37 137.18 136.64 135.77 135.57 135.53 134.46 134.41 133.56 133.54 133.01 131.06 130.12 129.92 129.35 128.45 128.37 128.21 128.19
50.05
21.04 20.51
Cl
H
αHM1TTM
ppm

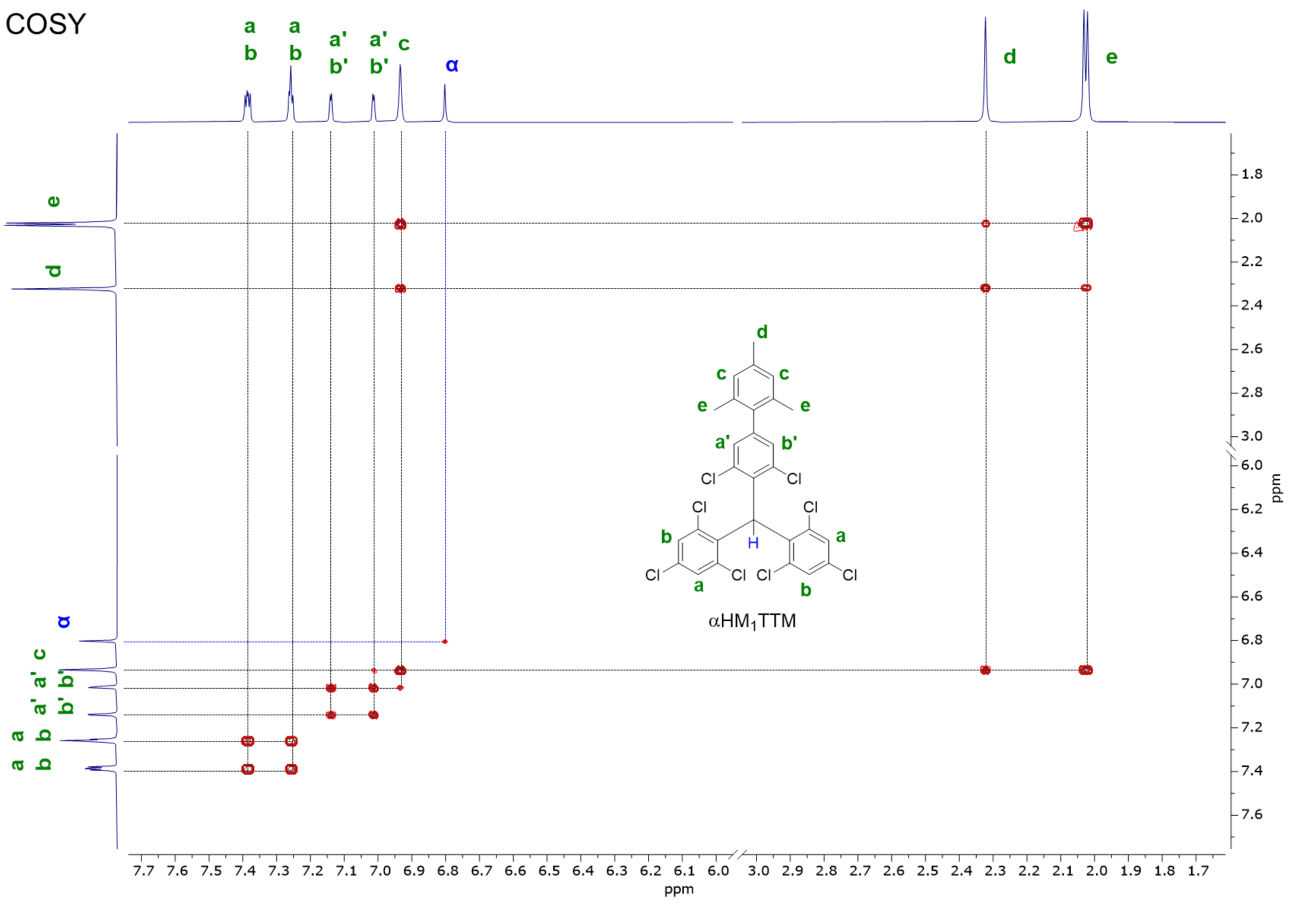
COSY
a
b
a'
b'
c
α
d
e
αHM1TTM
ppm

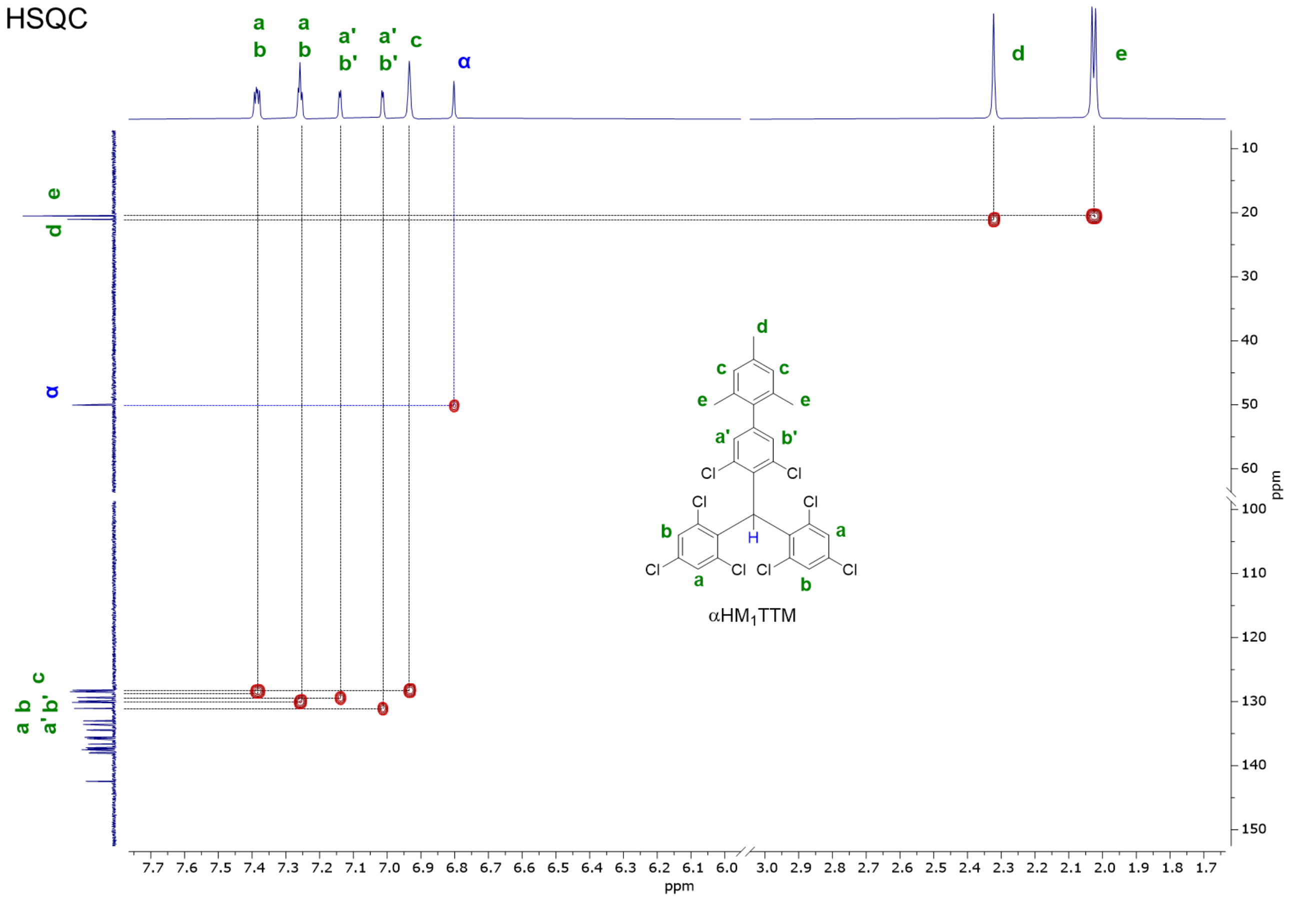
HSQC
a
b
a'
b'
c
α
d
e
αHM1TTM
ppm

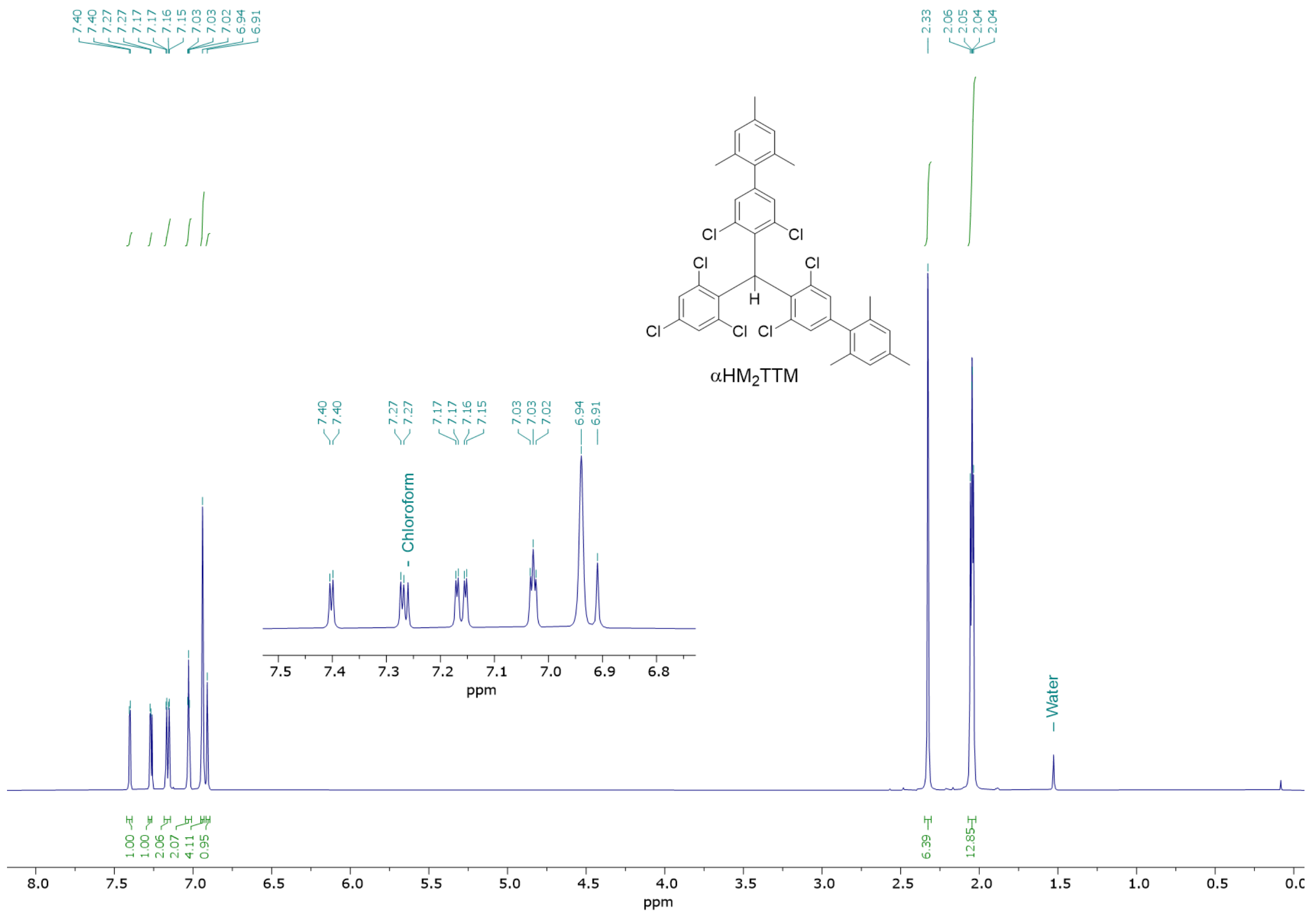
αHM2TTM
Chloroform
Water
ppm

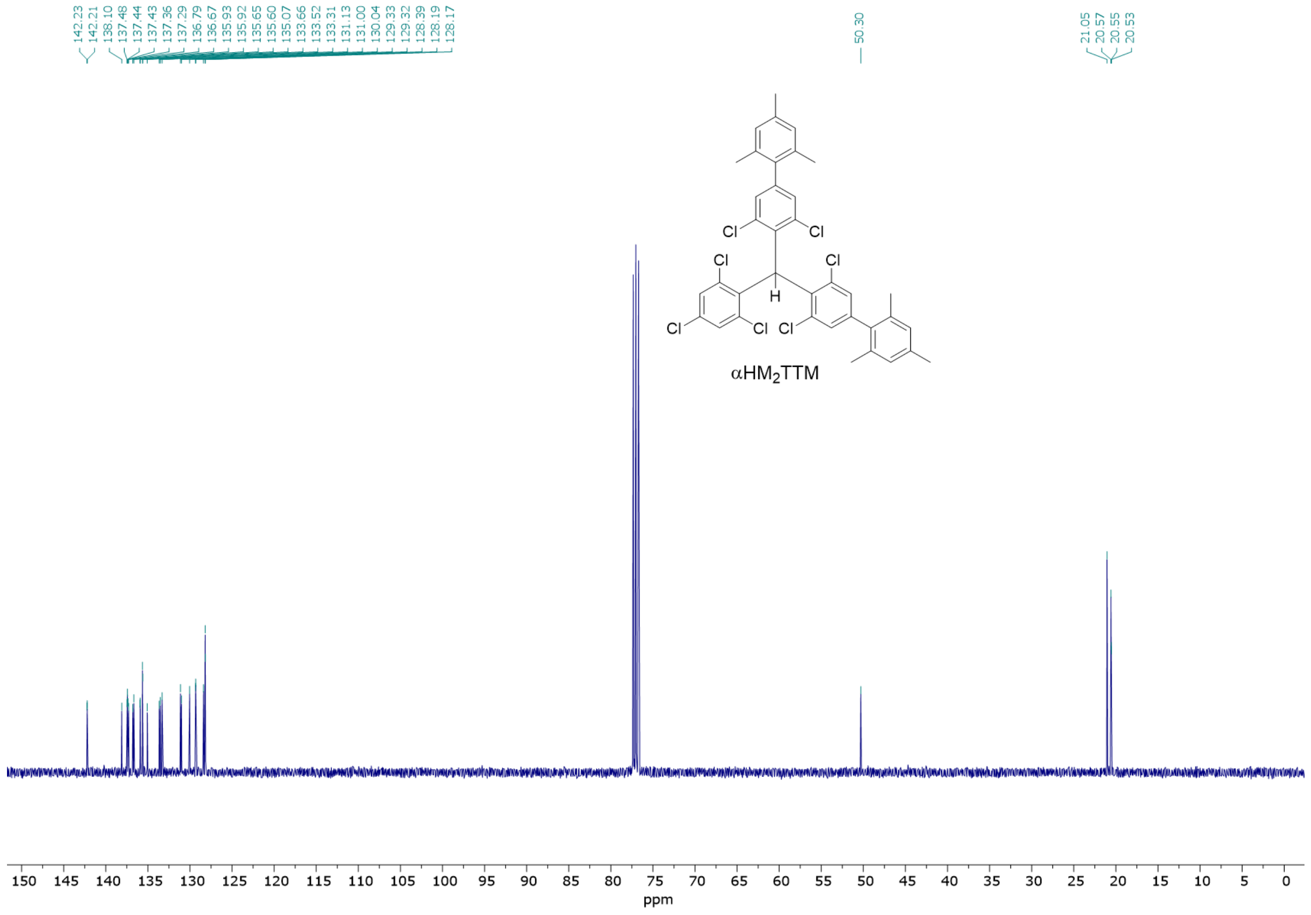
αHM2TTM
ppm

COSY

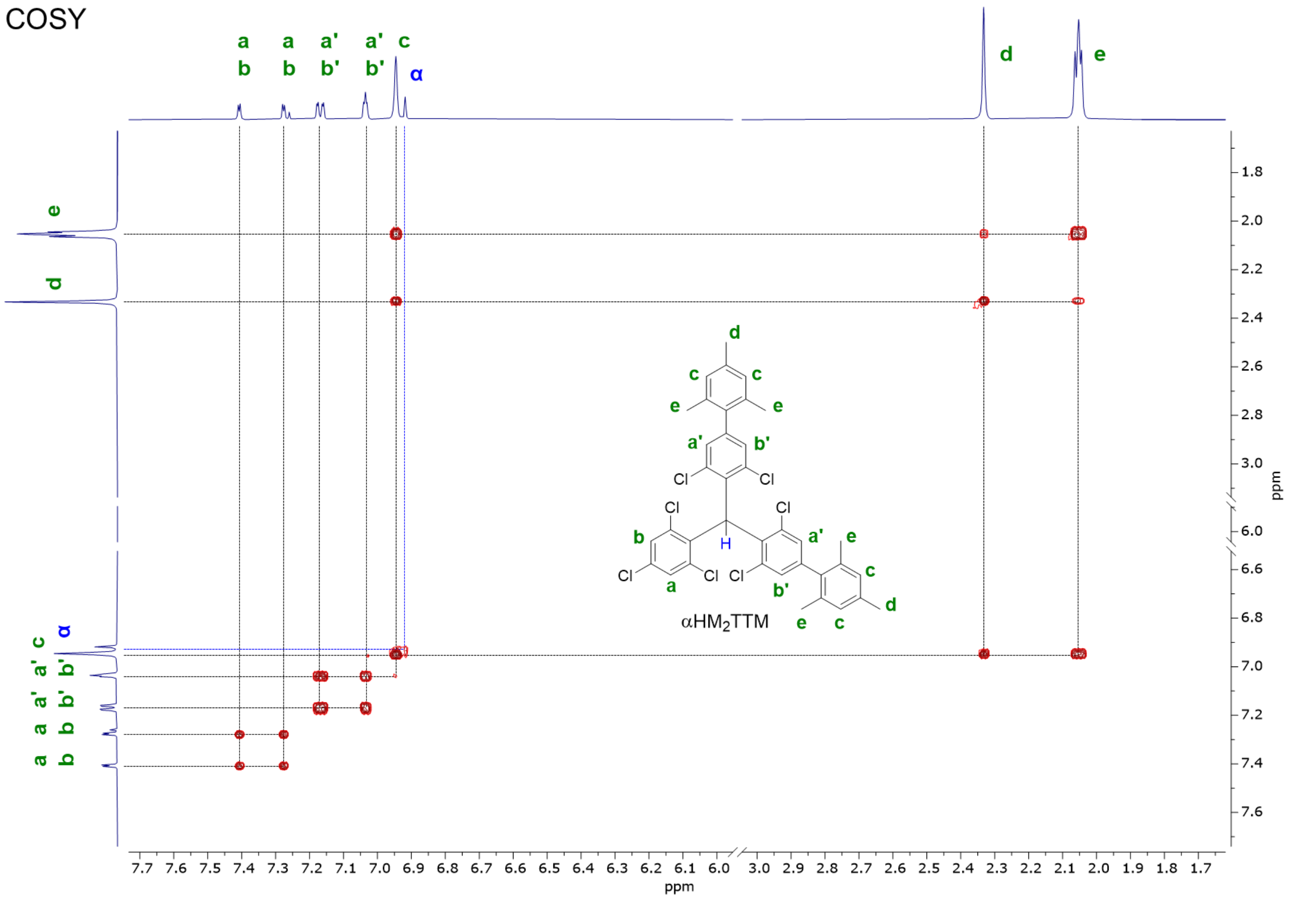

HSQC

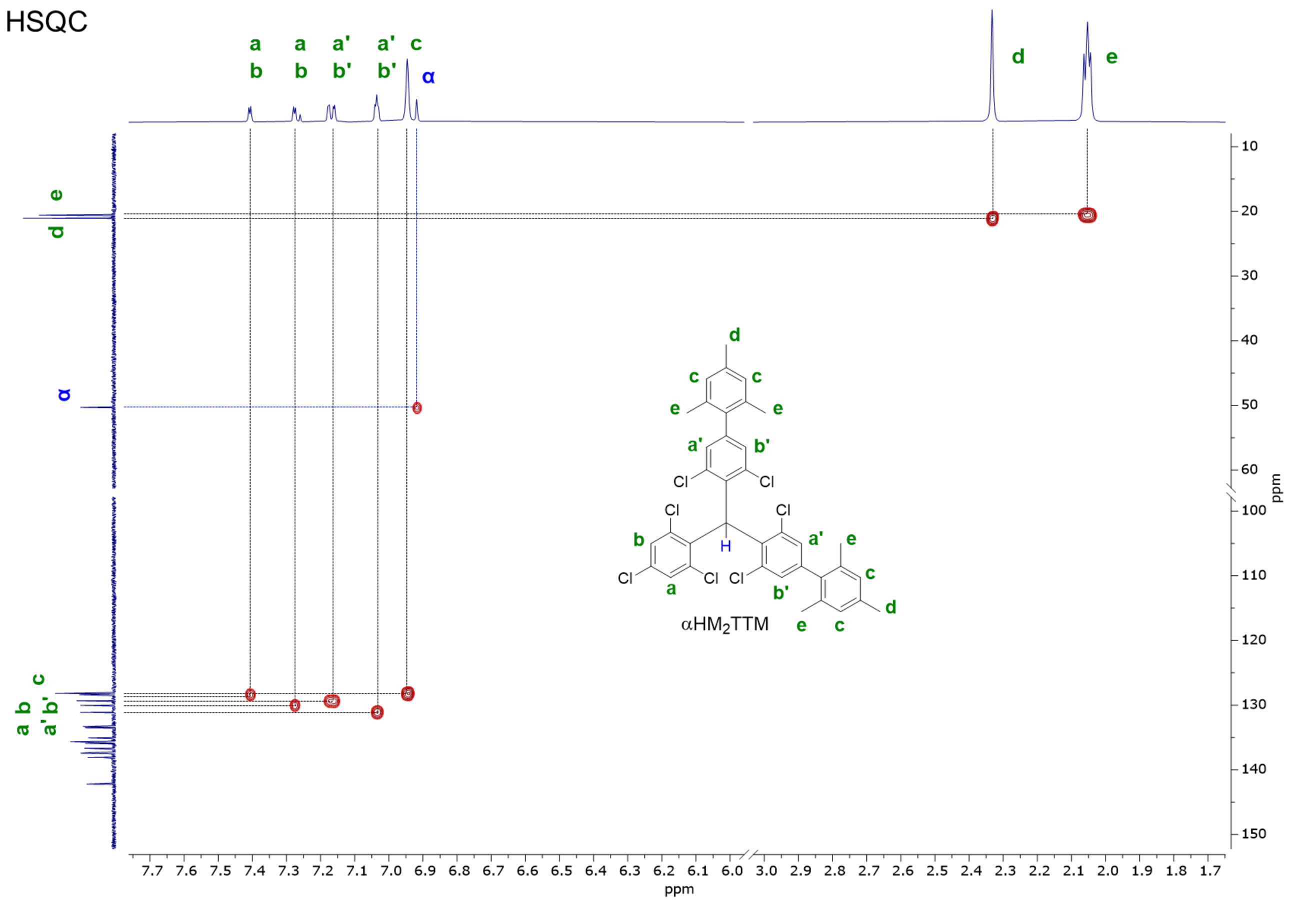

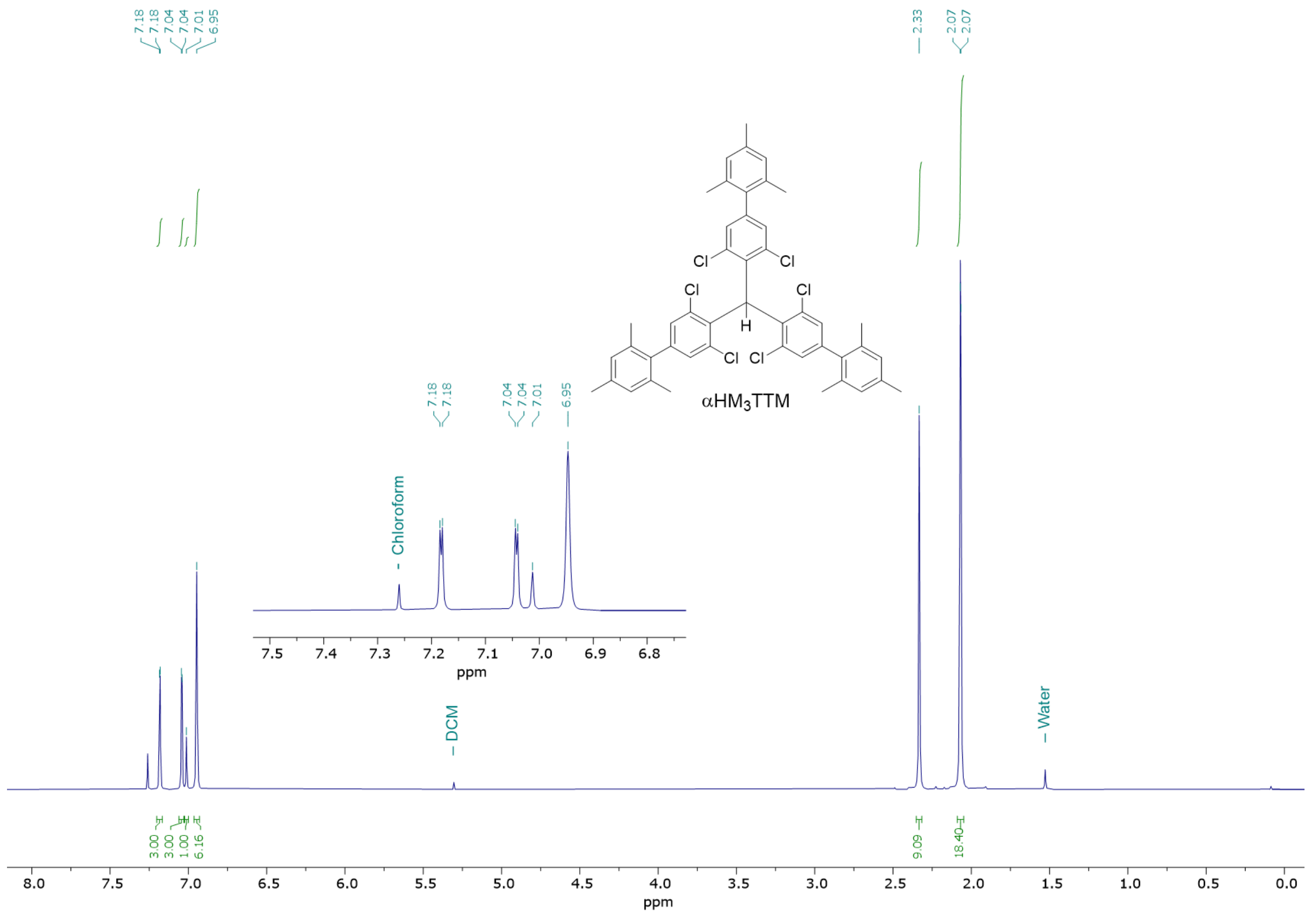
7.18
7.18
7.04
7.04
7.01
6.95
2.33
2.07
2.07
Chloroform
DCM
Water
αHM3TTM
3.00
3.00
1.00
6.16
9.09
18.40
ppm

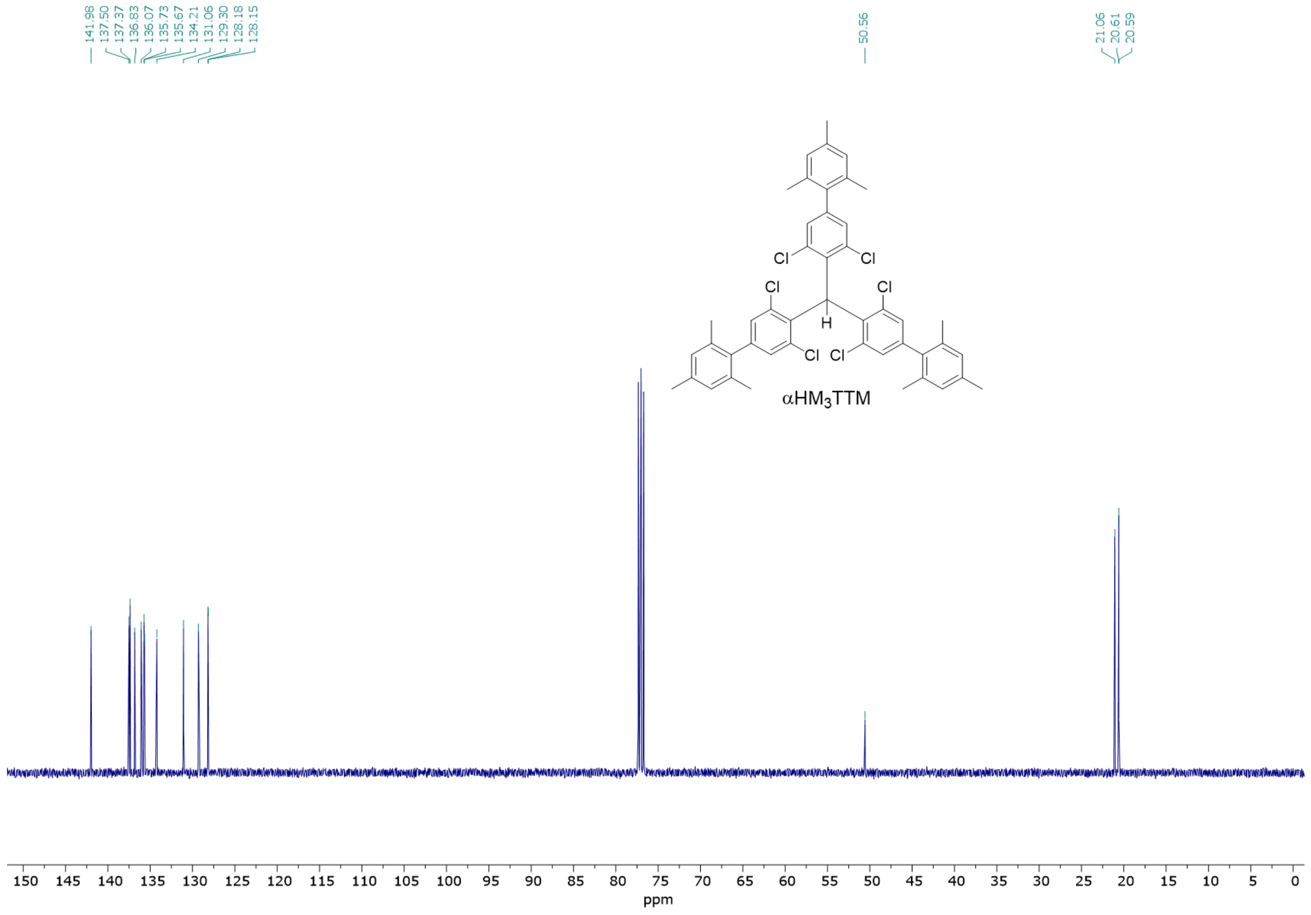
141.98
137.50
137.37
136.83
136.07
135.73
135.67
134.21
131.06
129.30
128.18
128.15
50.56
21.06
20.61
20.59
αHM3TTM
ppm

COSY

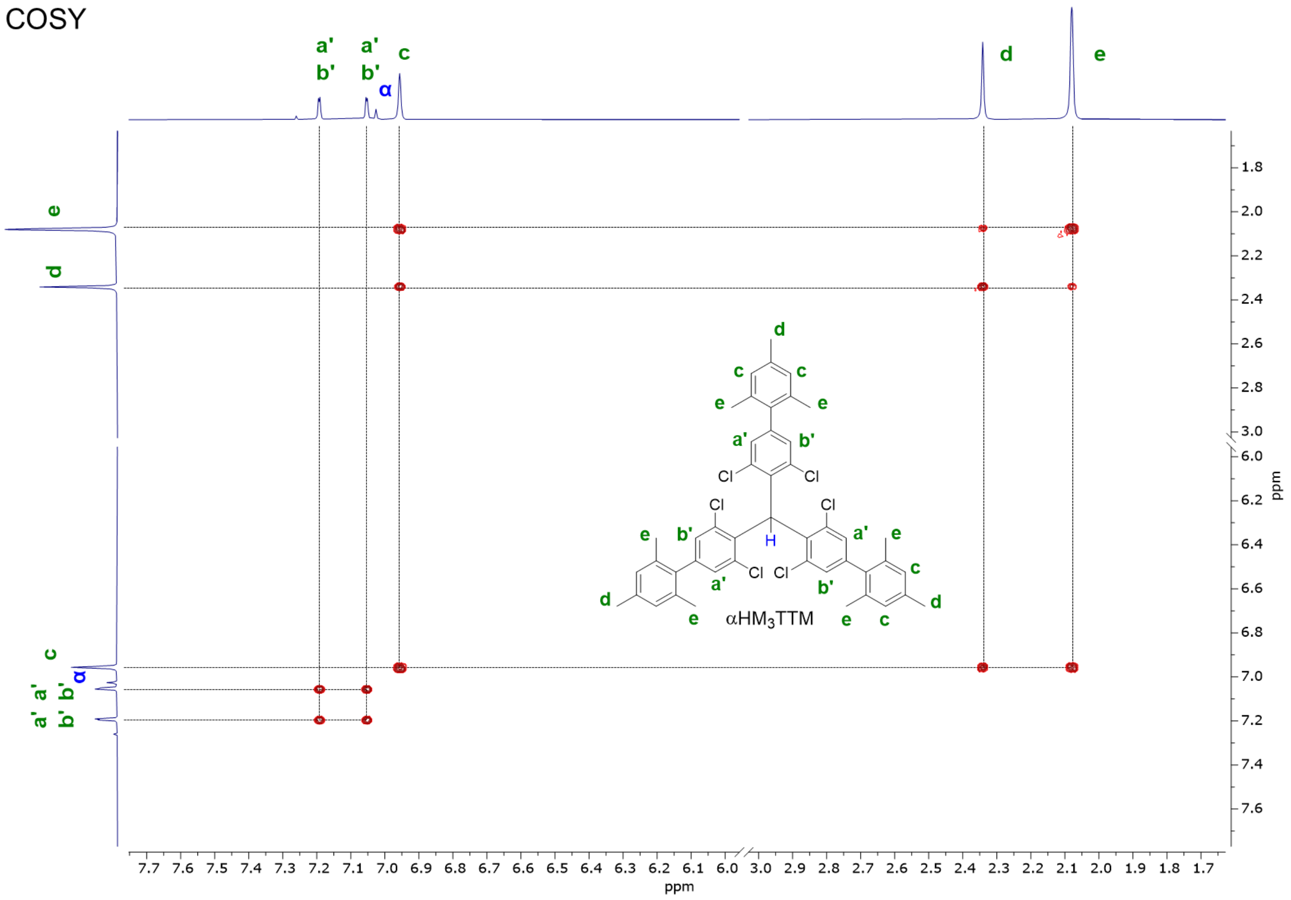

HSQC

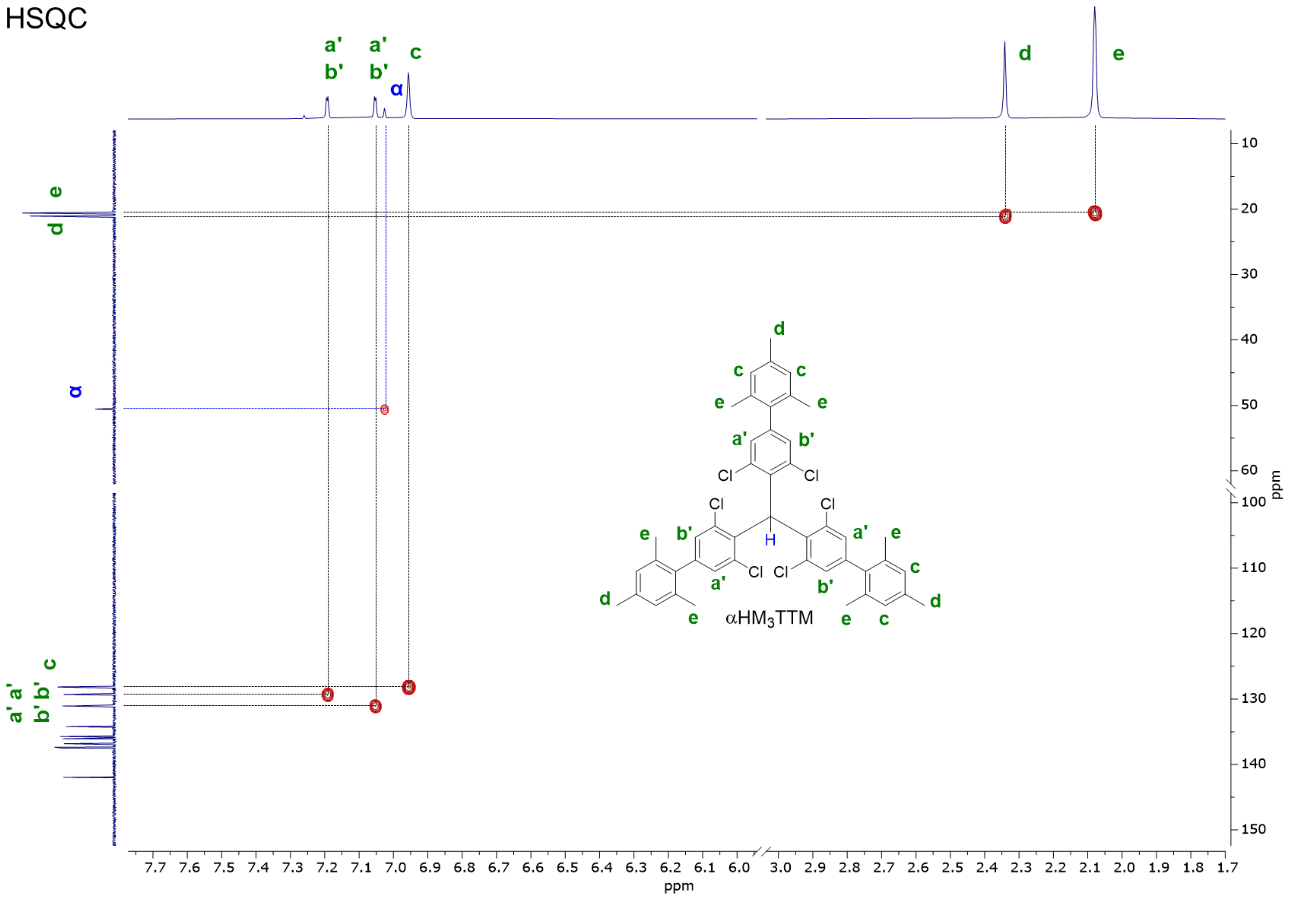

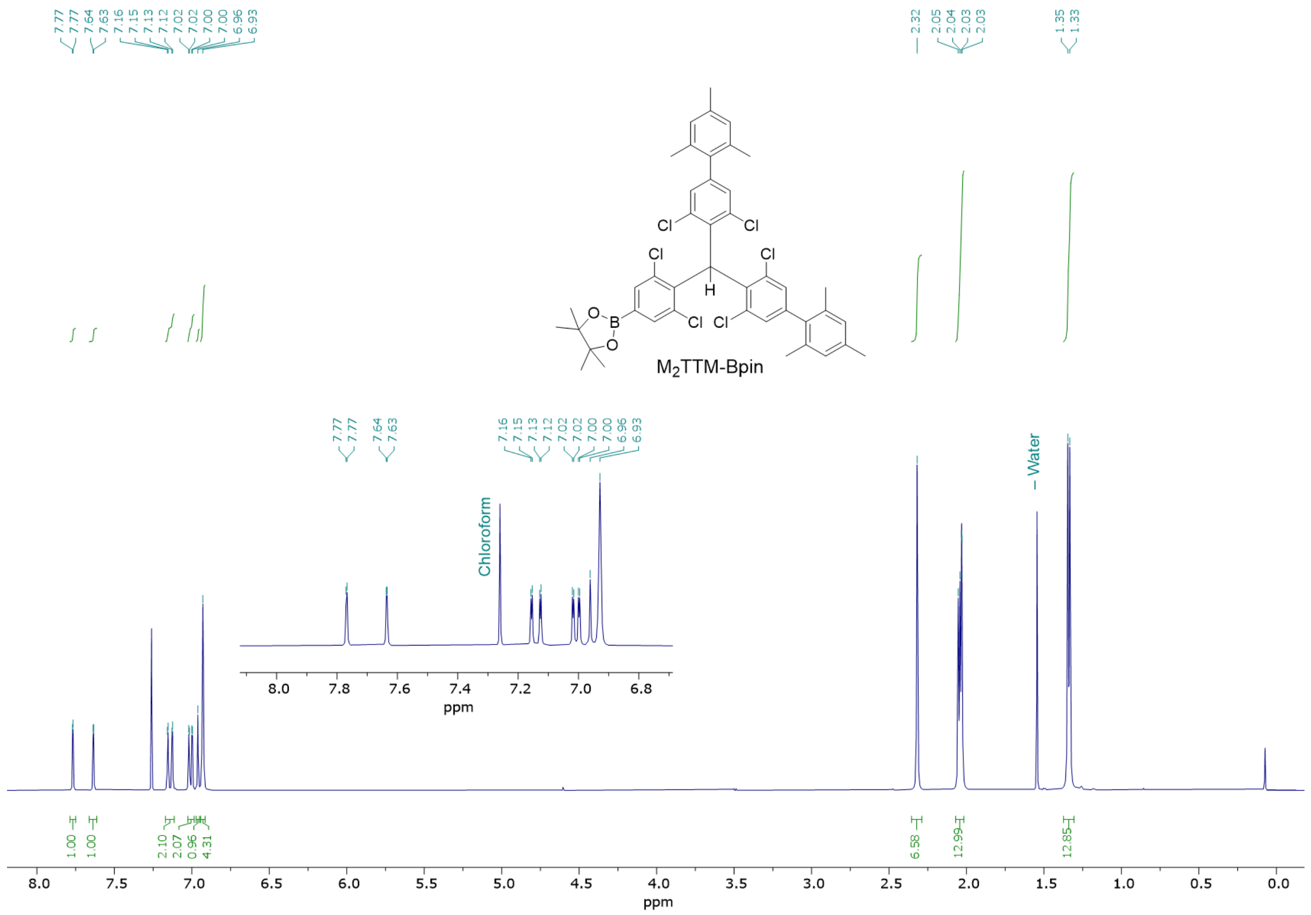

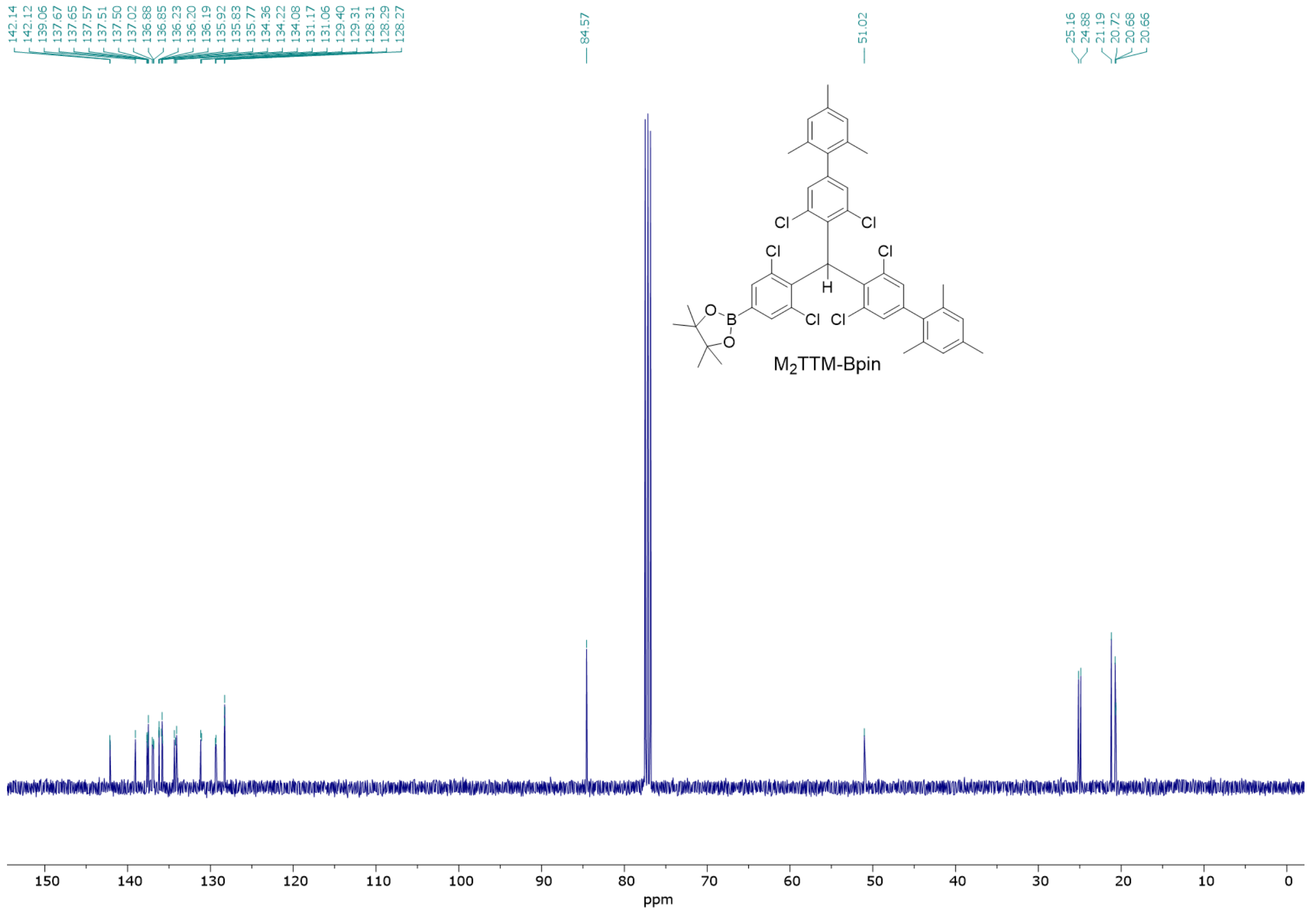

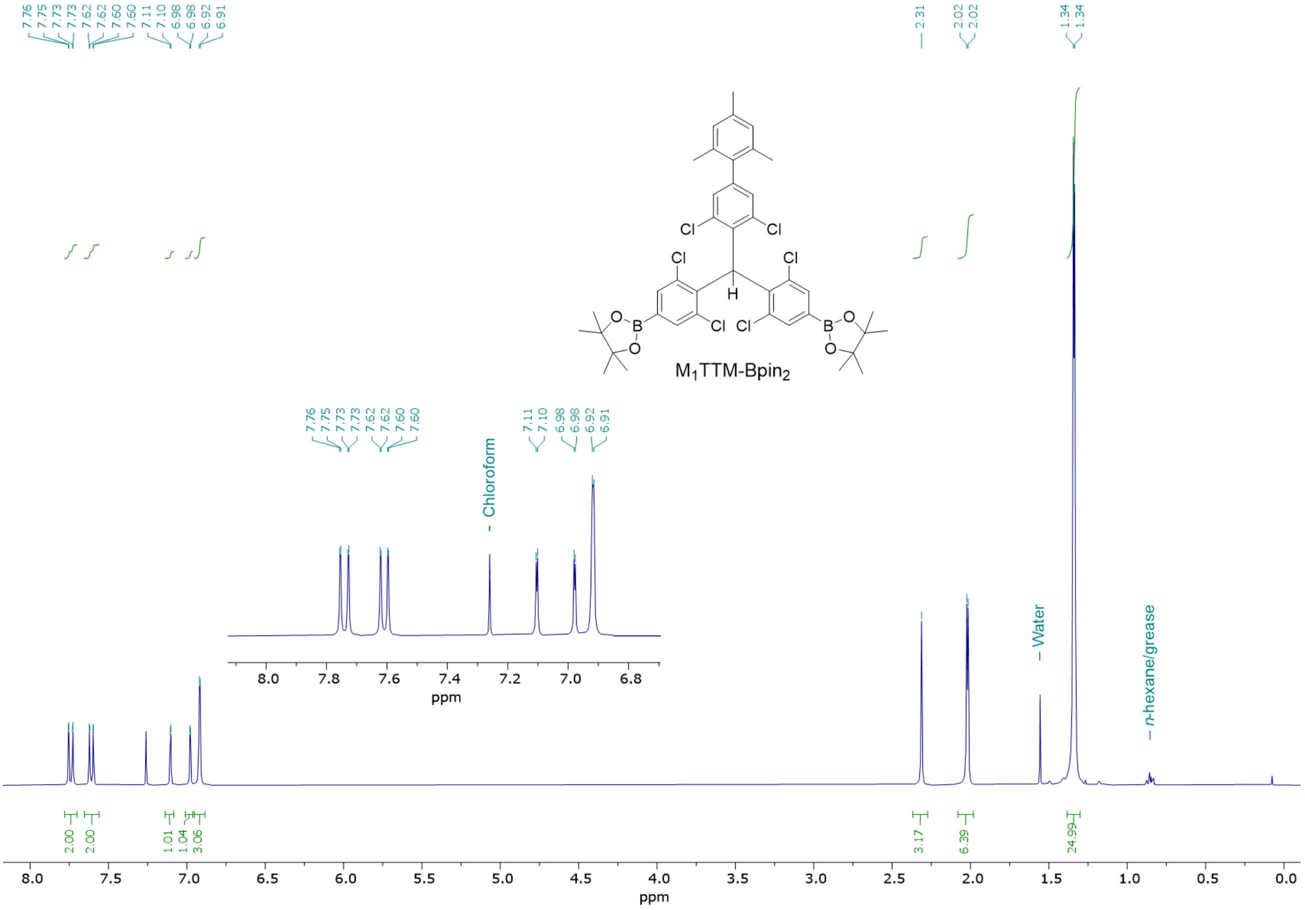
M$_1$TTM-Bpin$_2$
Chloroform
Water
n-hexane/grease
ppm

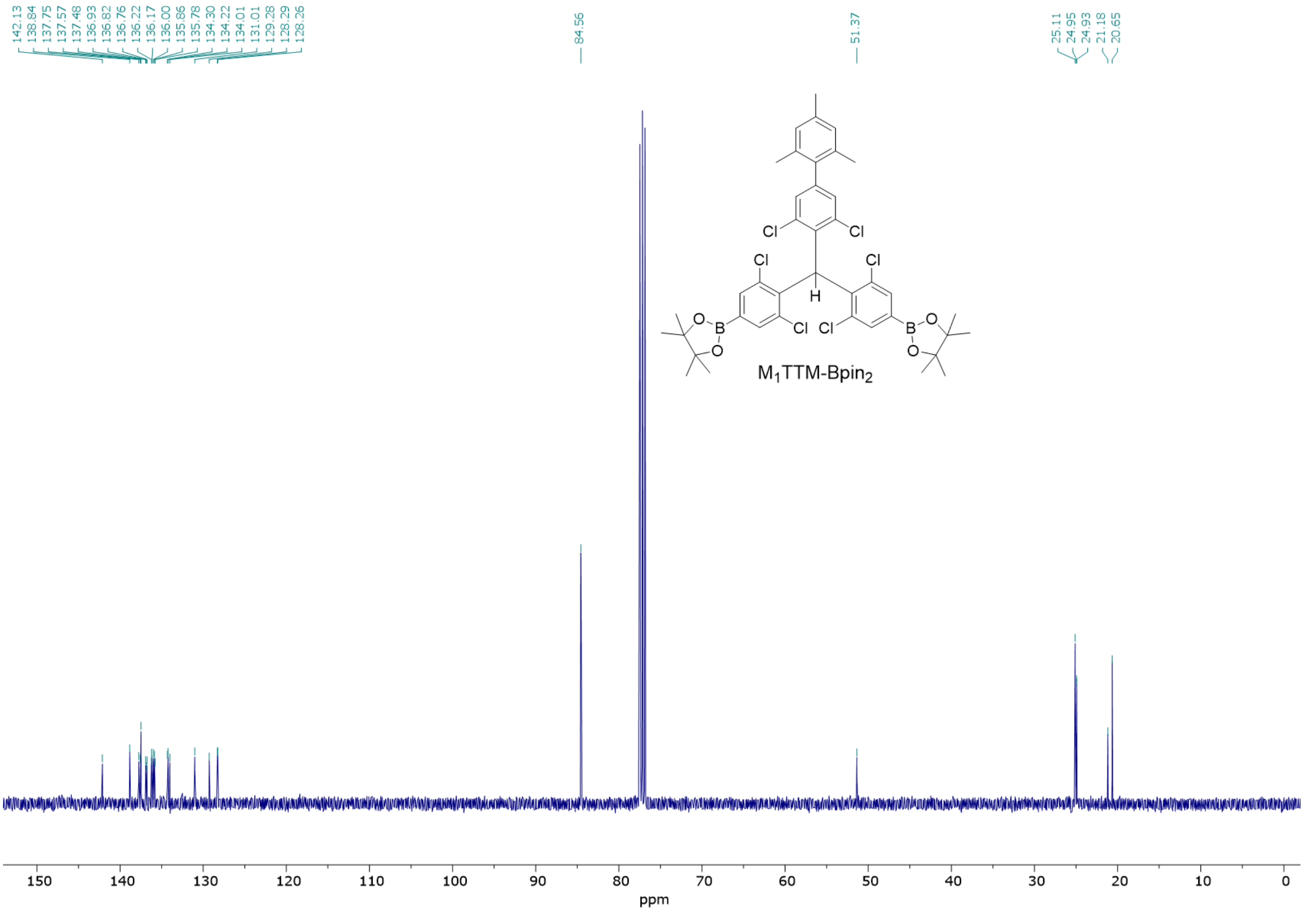
M$_1$TTM-Bpin$_2$
ppm

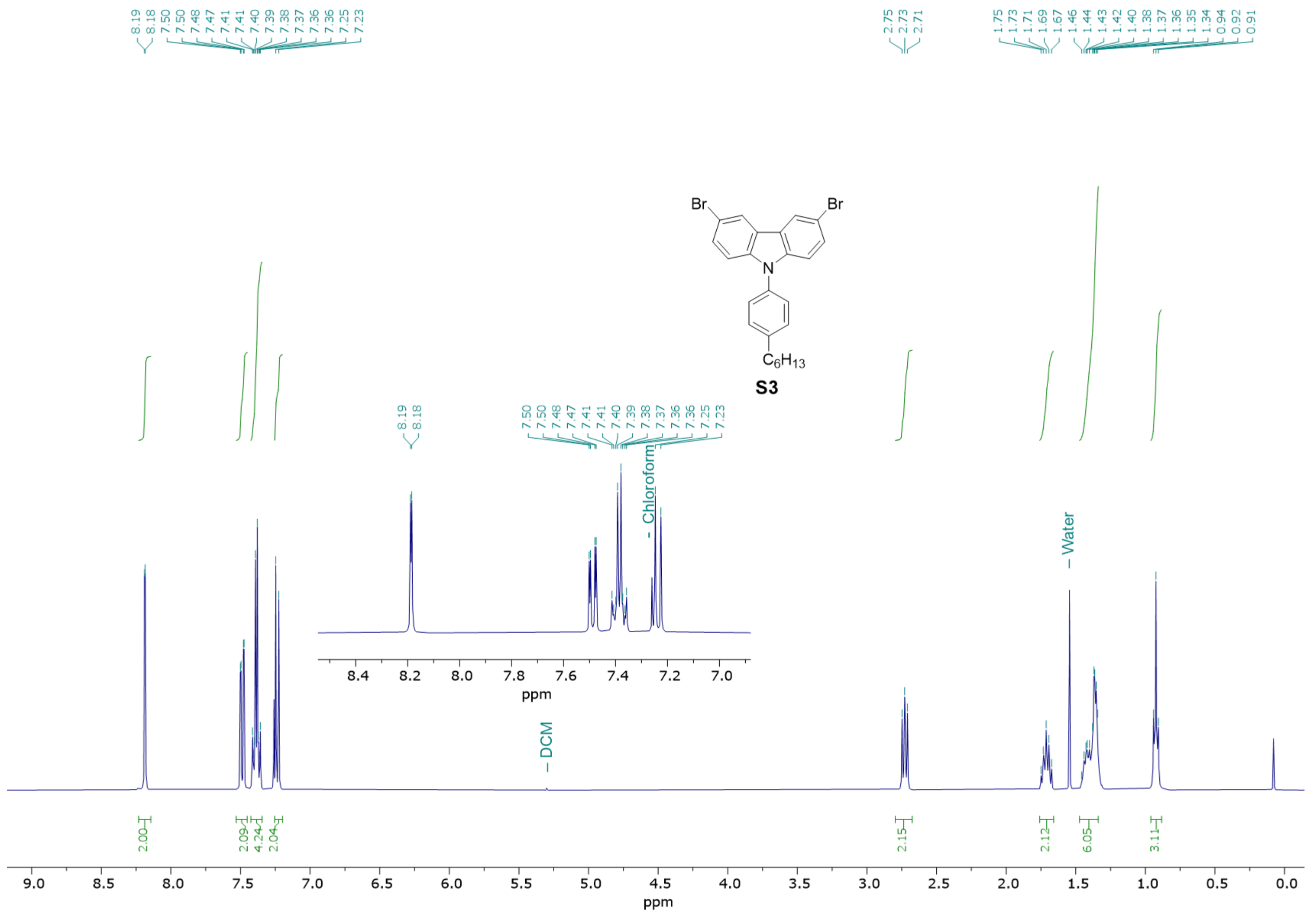

S3
DCM
Chloroform
Water
ppm

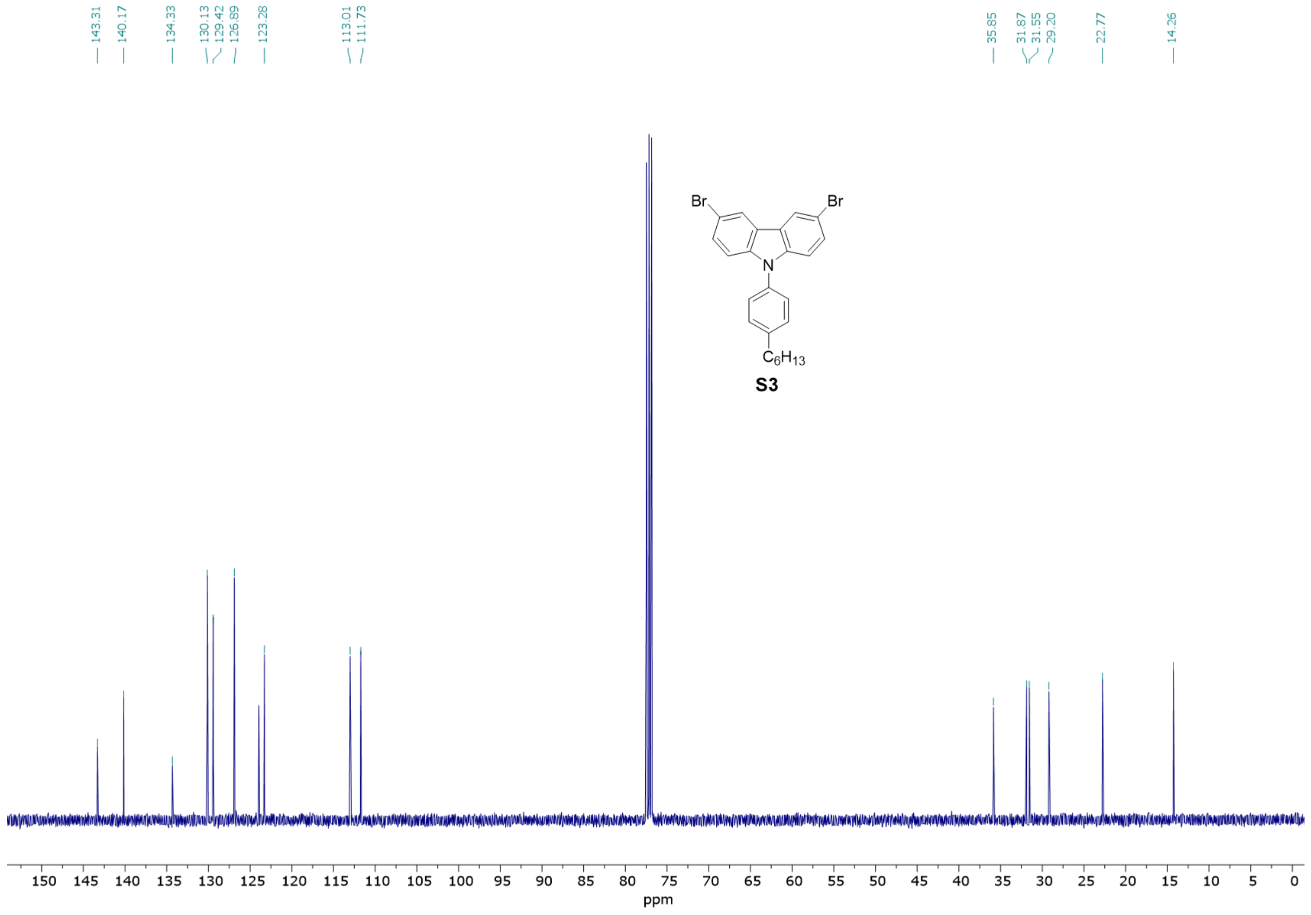

S3
ppm

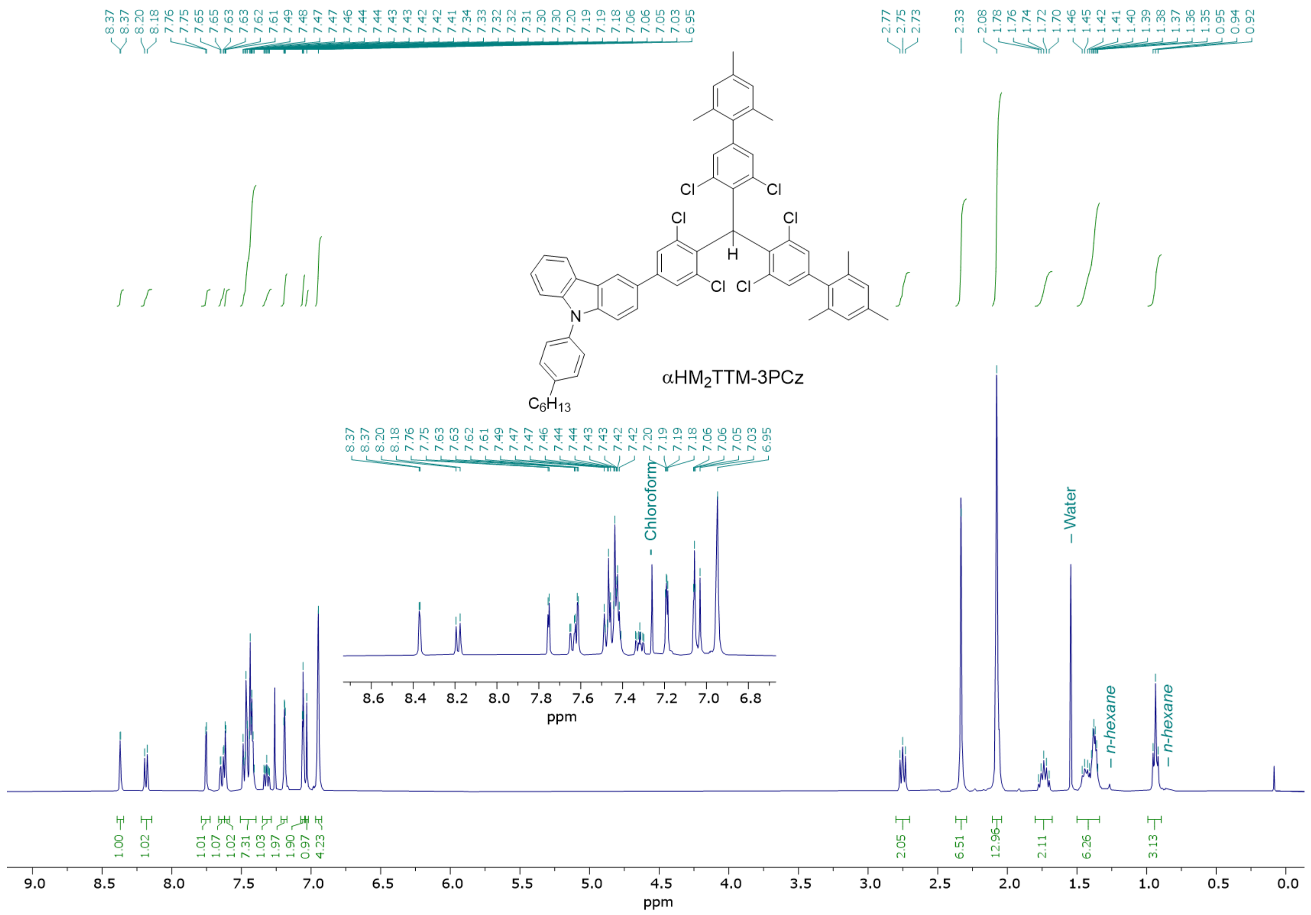
αHM$_2$TTM-3PCz
Chloroform
Water
n-hexane
n-hexane
ppm

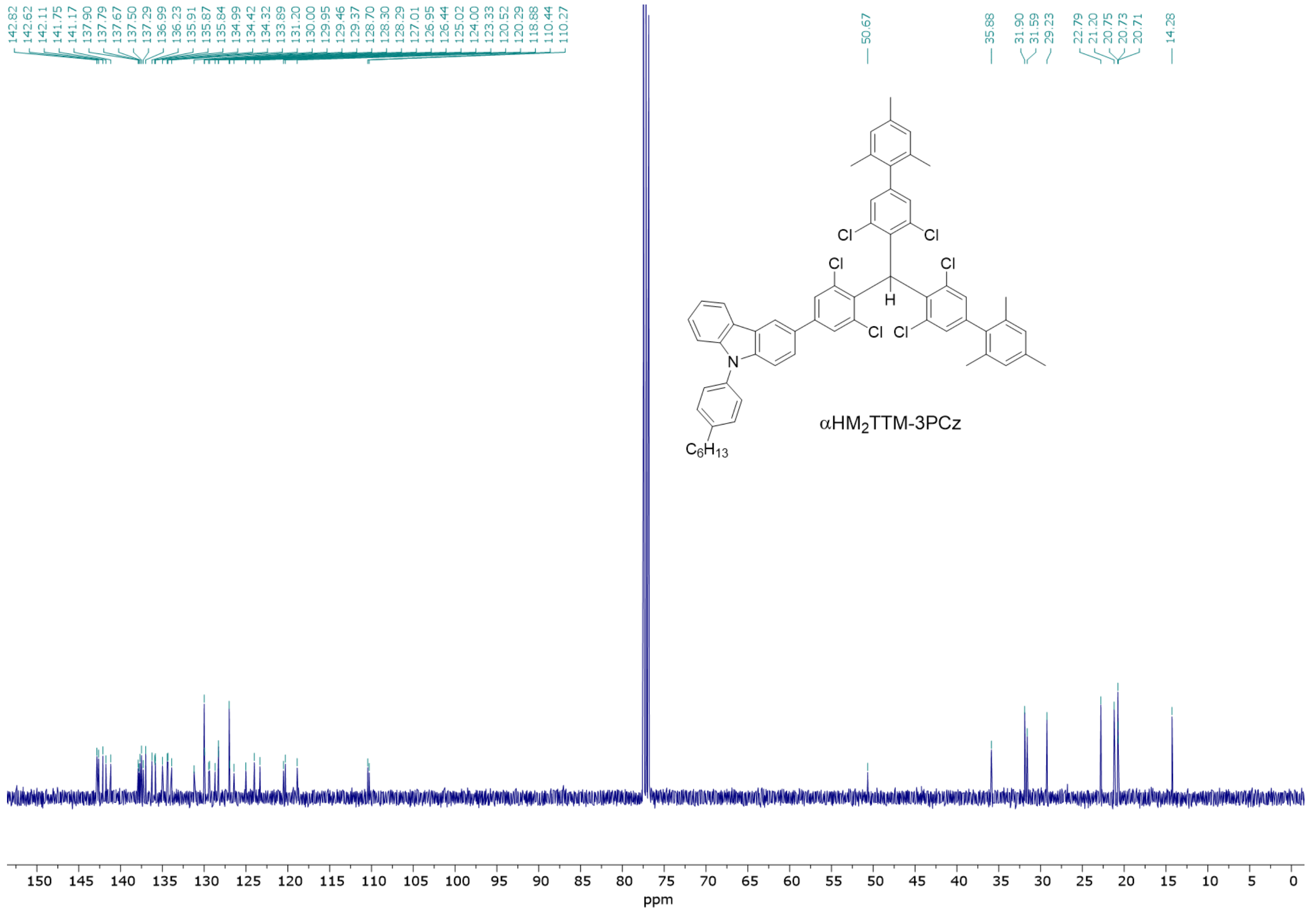
αHM$_2$TTM-3PCz
ppm

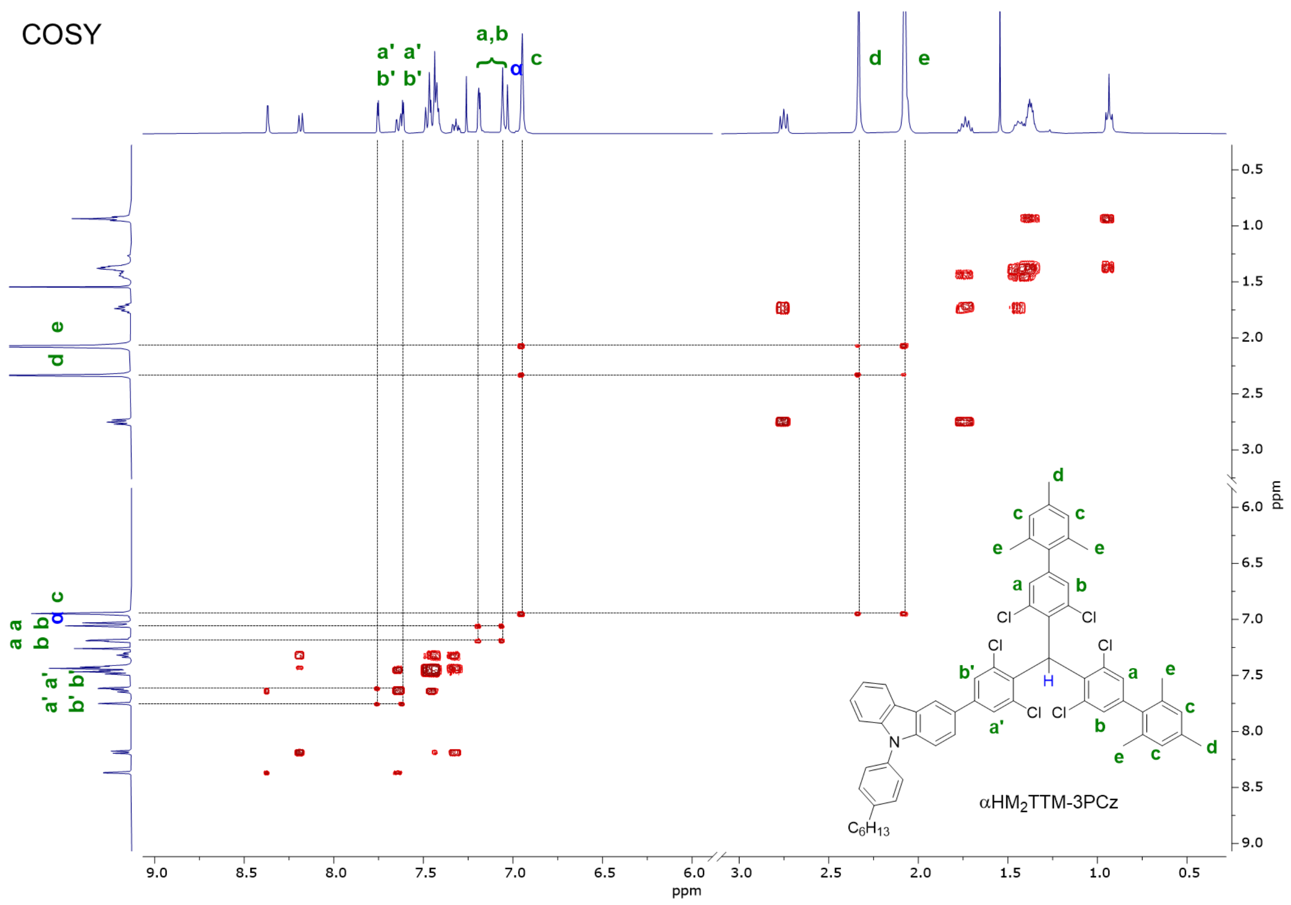
COSY
a' b' a' b'
a,b
α
c
d
e
αHM$_2$TTM-3PCz
C_6H_{13}
ppm

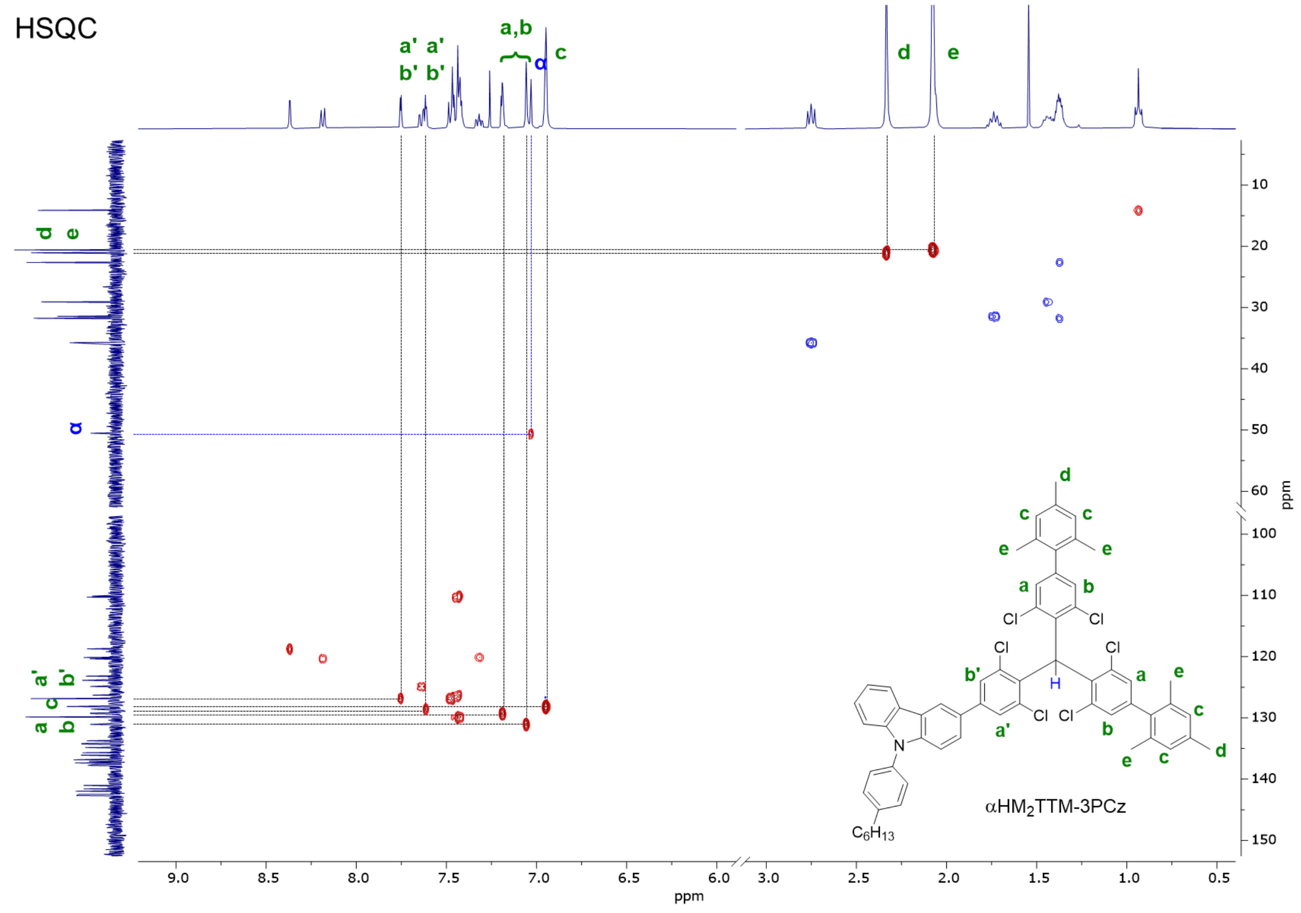
HSQC
a' b' a' b'
a,b
α
c
d
e
αHM$_2$TTM-3PCz
C_6H_{13}
ppm

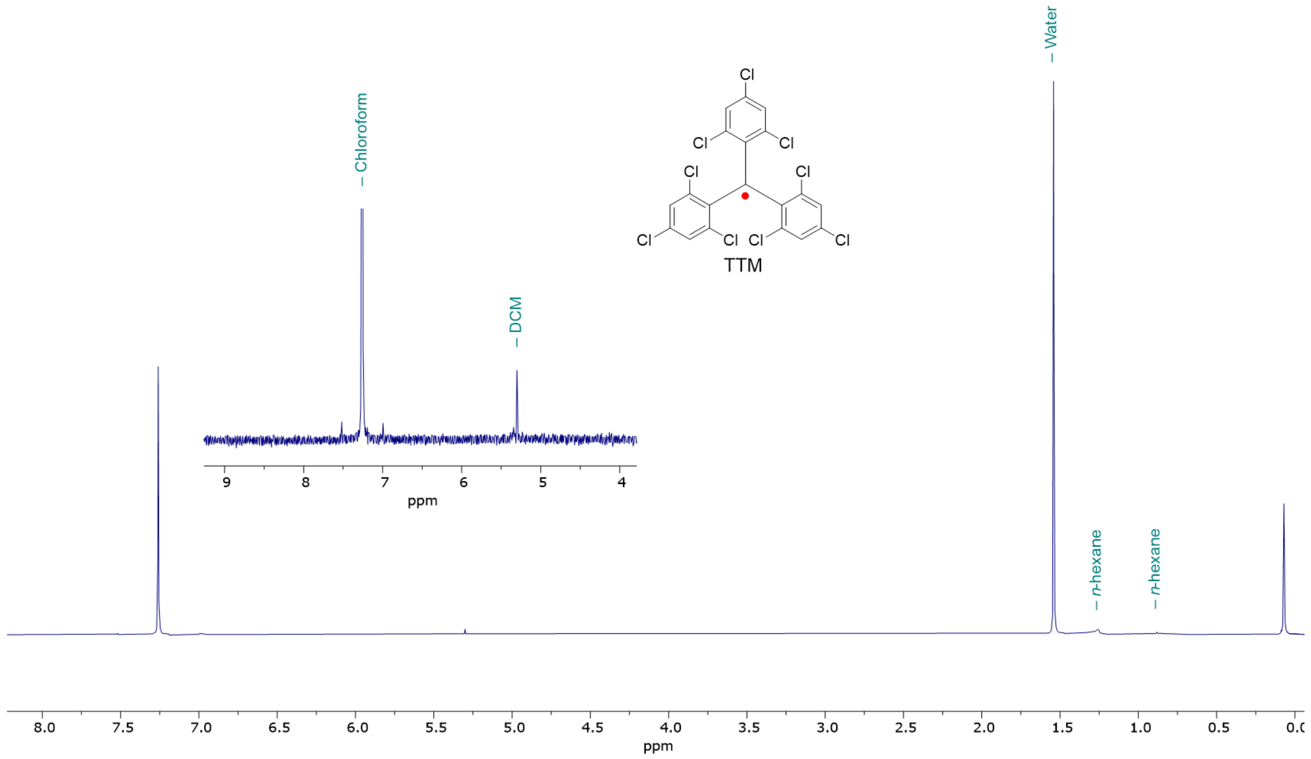

– Water
– Chloroform
– DCM
TTM
– n-hexane
– n-hexane
ppm

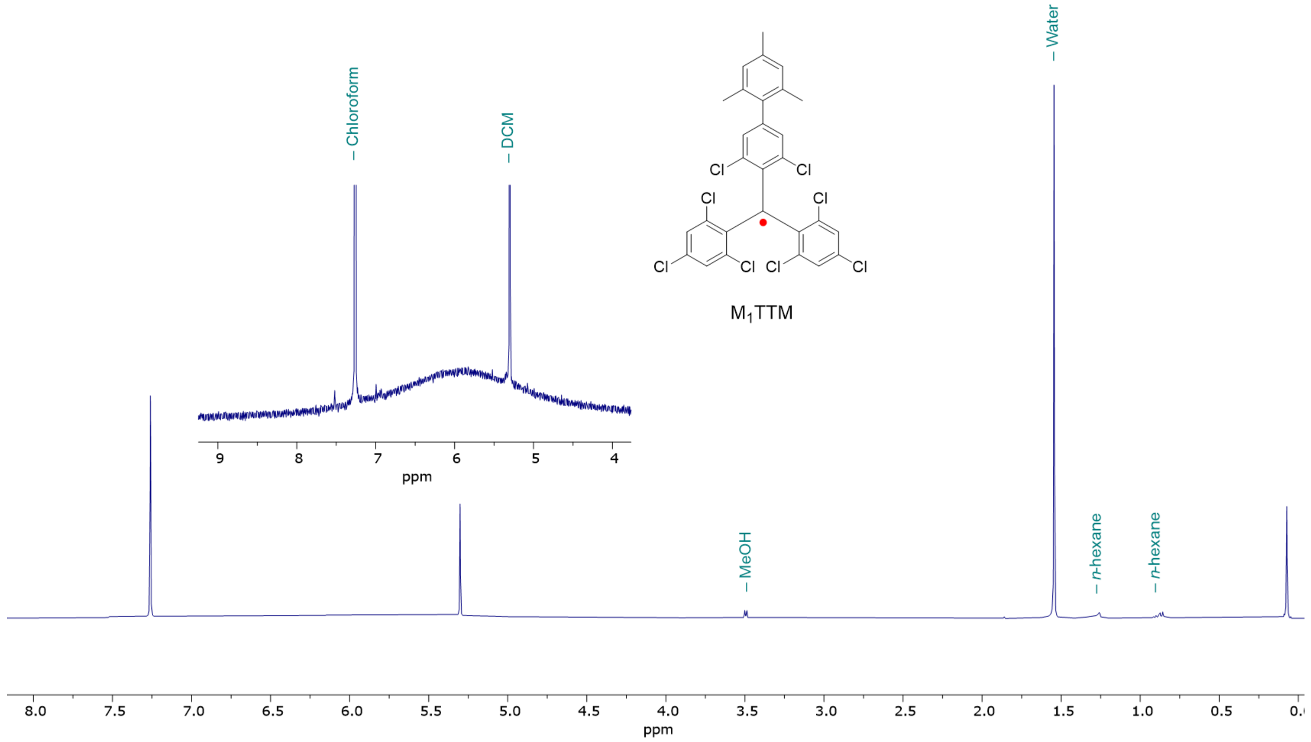

– Water
– Chloroform
– DCM
M_1TTM
– MeOH
– n-hexane
– n-hexane
ppm

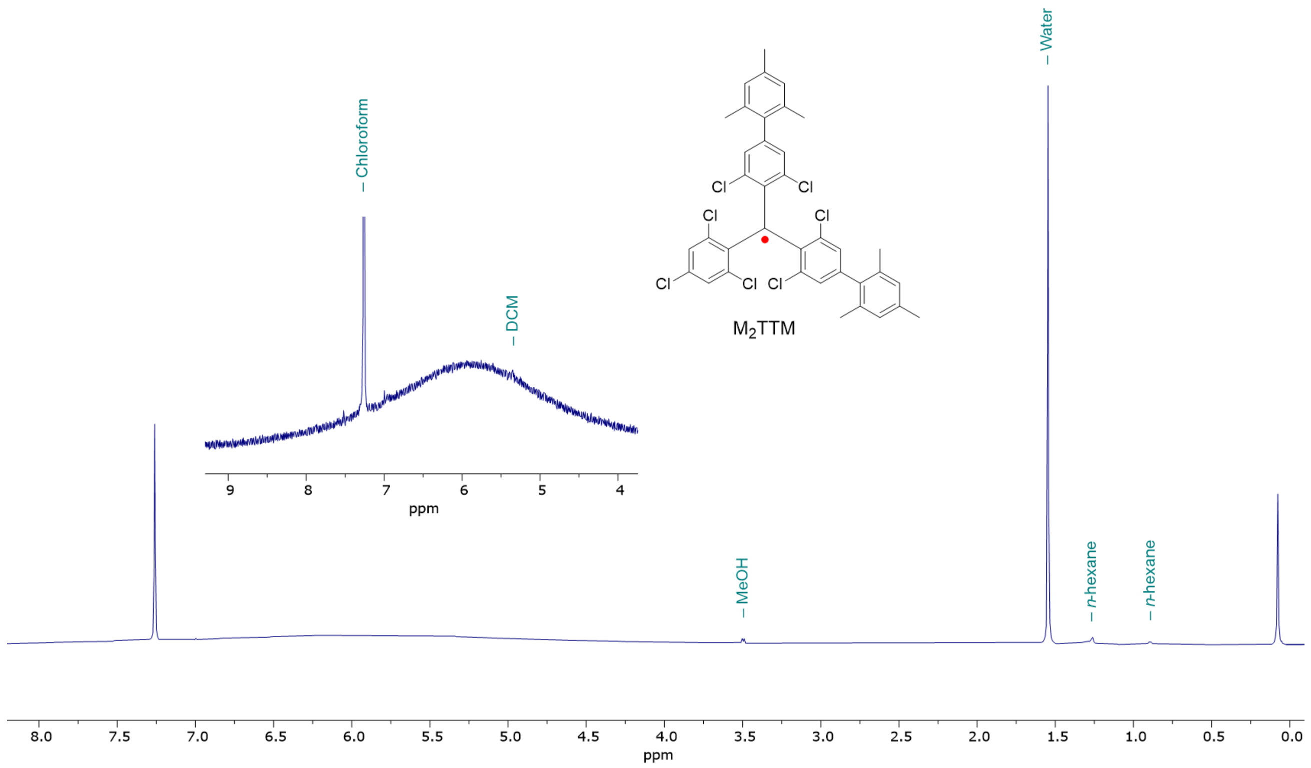

Water
Chloroform
DCM
MeOH
n-hexane
n-hexane
M$_2$TTM
ppm

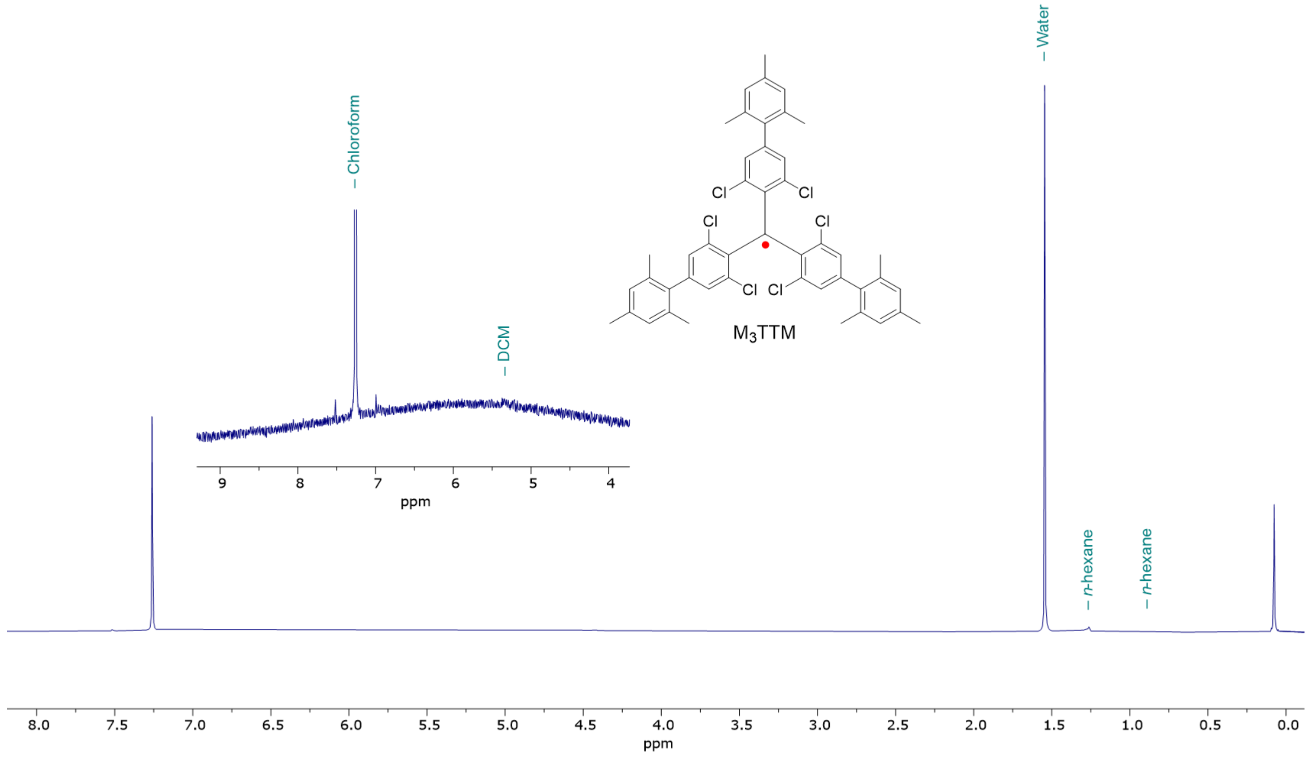

Water
Chloroform
DCM
n-hexane
n-hexane
M$_3$TTM
ppm

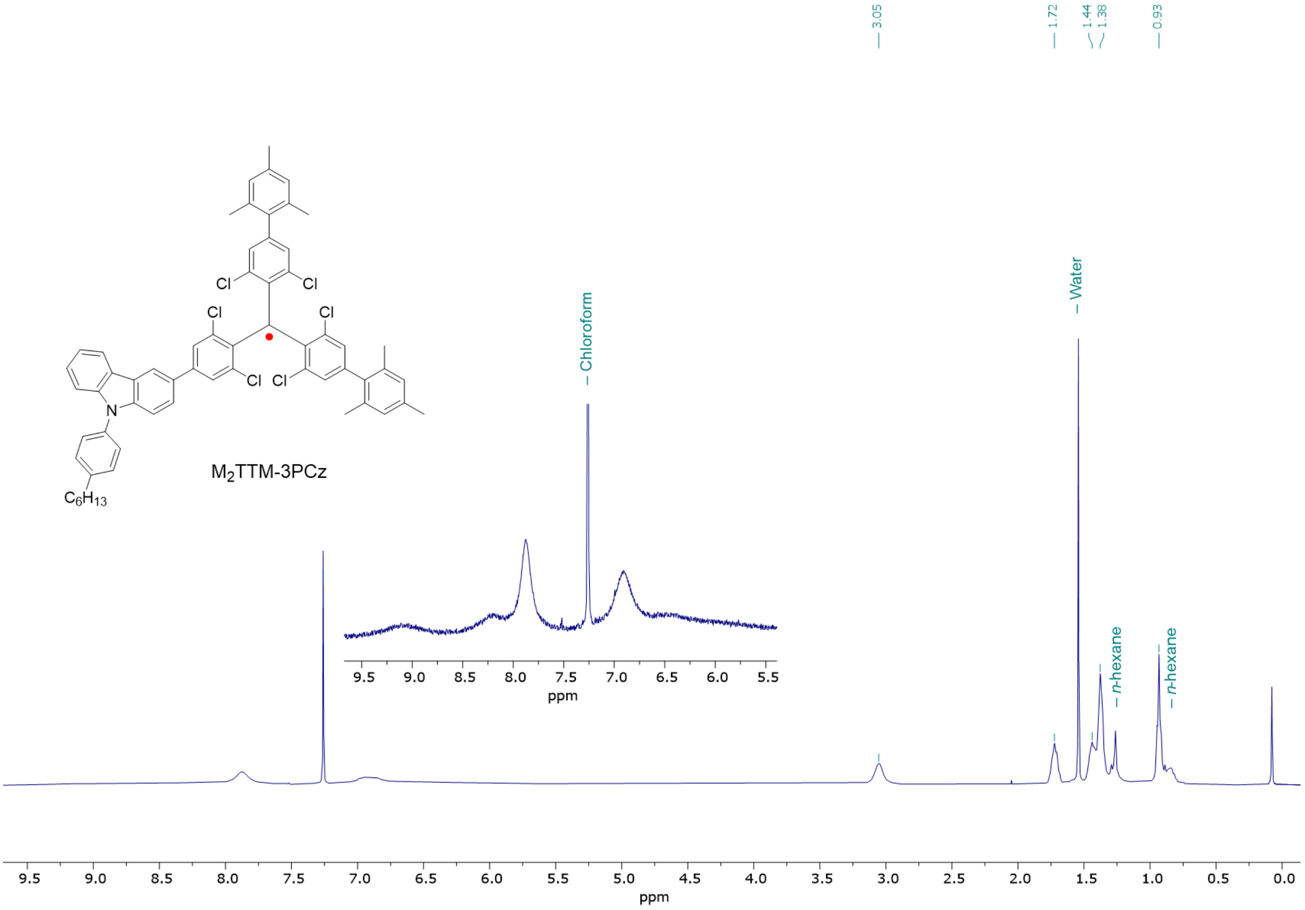
M$_2$TTM-3PCz
Chloroform
Water
n-hexane
ppm

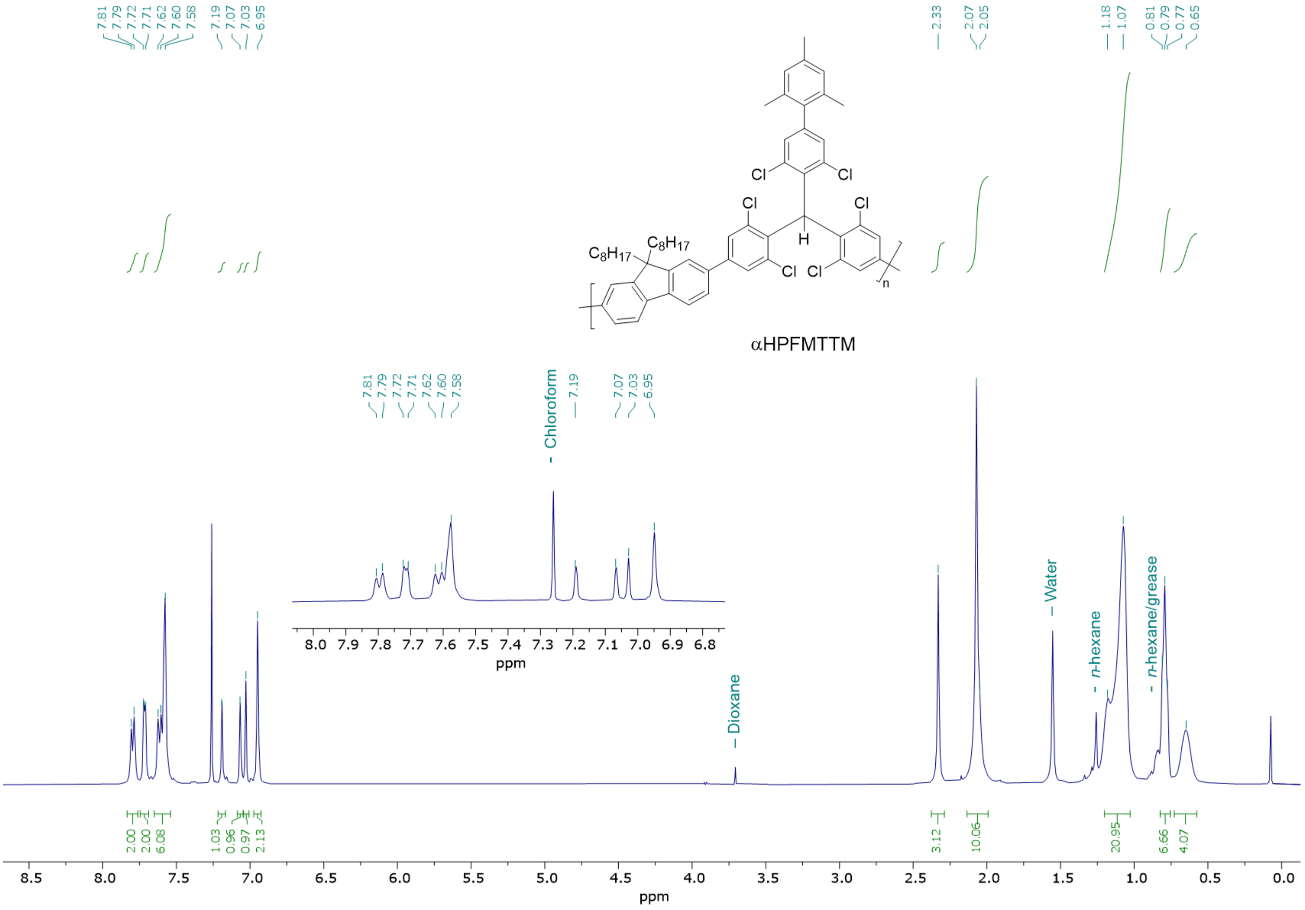
αHPFMTTM
Chloroform
Dioxane
Water
n-hexane
n-hexane/grease
ppm

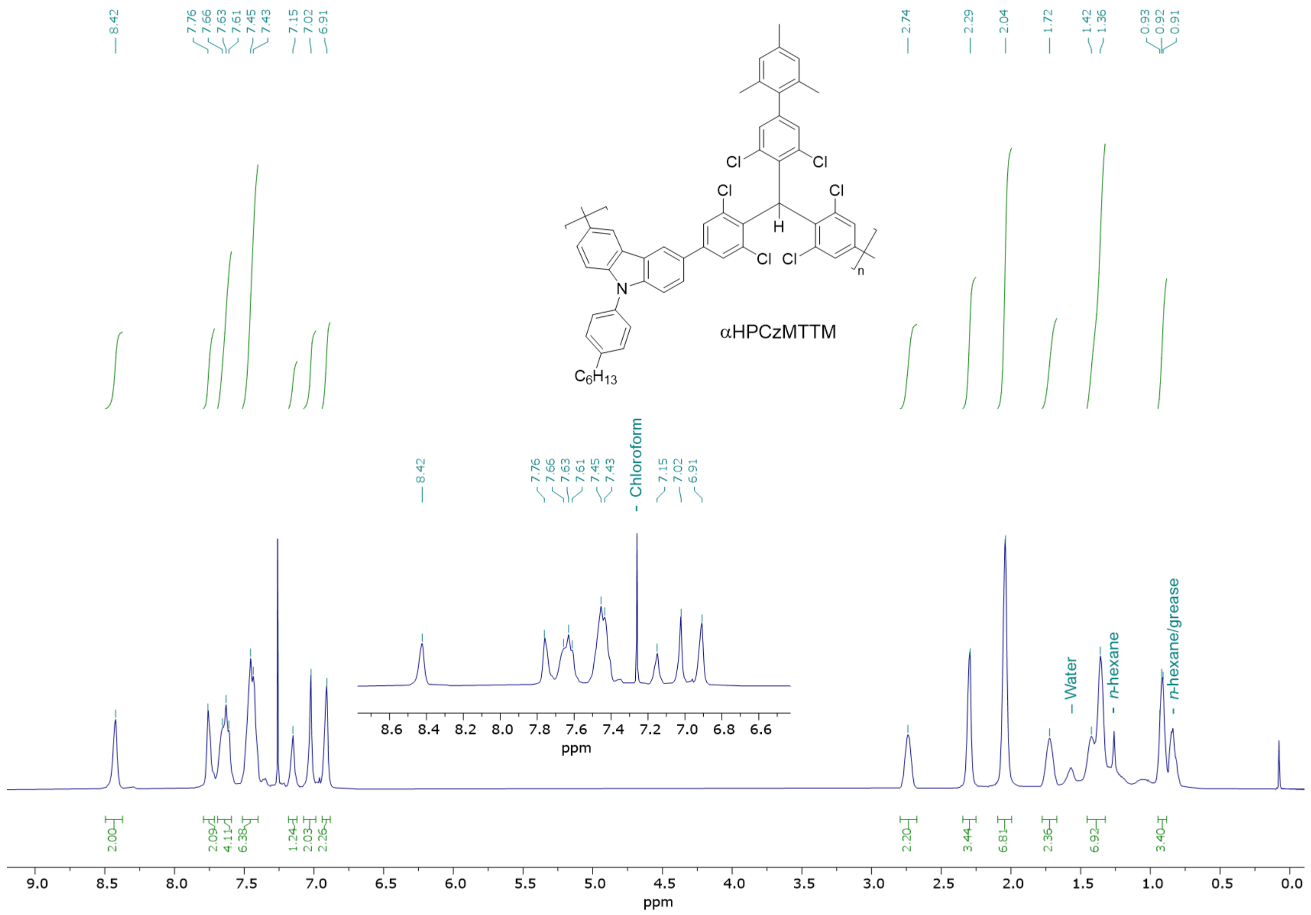
αHPCzMTTM
Chloroform
Water
n-hexane
n-hexane/grease
ppm

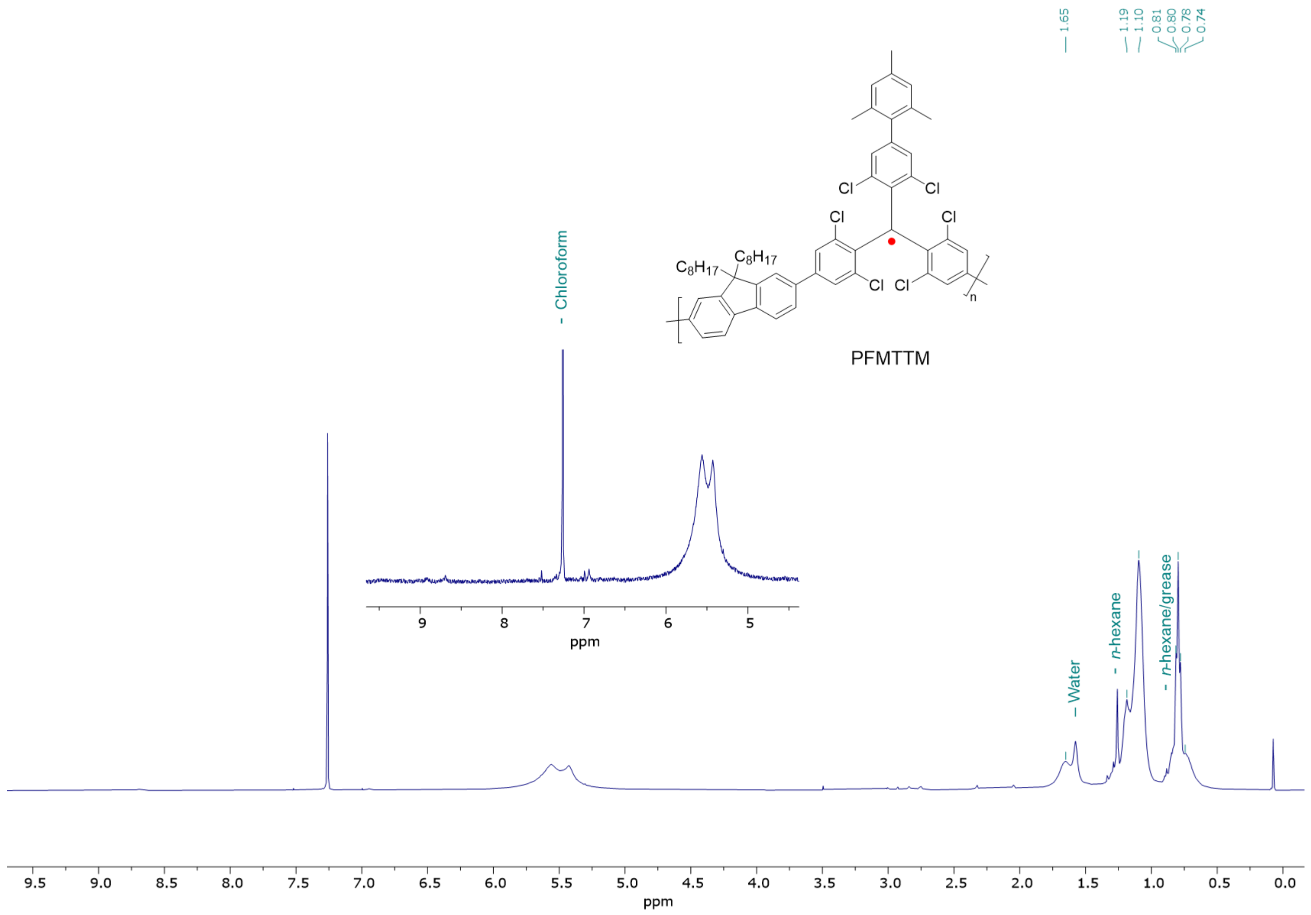
PFMTTM
Chloroform
Water
n-hexane
n-hexane/grease
ppm

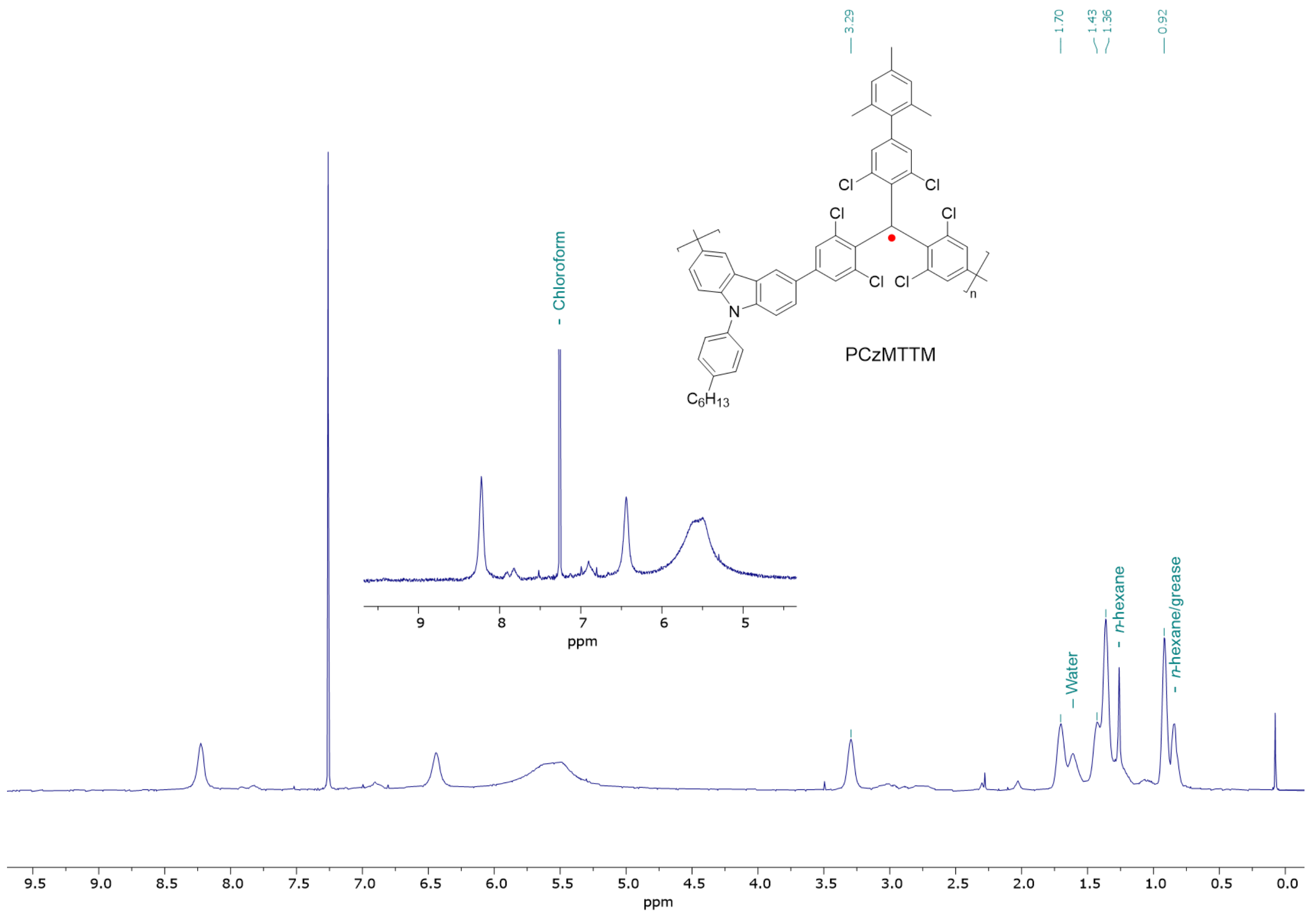
3.29
1.70
1.43
1.36
0.92
PCzMTTM
Chloroform
Water
n-hexane
n-hexane/grease
ppm
ppm

Supplementary Note 3: X-Ray Crystallography

TTM. The crystal structure of TTM at room temperature has been reported previously (CSD: GAHSOX).[11] The structure reported here (at 180 K) does not differ significantly from the room-temperature structure. The molecule is situated on a crystallograpic 2-fold axis, passing through atoms C1/C2/C5/Cl2.

M_1TTM. The molecule is situated on a crystallograpic 2-fold axis, passing through atoms C1/C2/C5/C6/C9/C10. H atoms of the Me group on C10 are placed geometrically and refined as riding, with rotation allowed around the local 3-fold axis of the Me group. The imposed crystallographic 2-fold symmetry results in disorder, giving two apparent orientations of the Me group.

M_2TTM. The molecule is situated on a general position within the crystal structure. The core of the molecule retains close 2-fold rotation symmetry, passing through atoms C1/C2/C5/Cl2, but the symmetry is effectively broken by the outer mesityl groups.

M_3TTM. Structure solution and refinement of the M_3TTM radical proceeded without difficulty. The molecule is situated on a crystallograpic 2-fold axis, passing through atoms C1/C2/C5/C6/C9/C10. The structure contains very large channels running along the *c* axis (= the 3-fold axis in the trigonal crystal structure), summing to approx. one third of the unit-cell volume. These voids contain $CDCl_3$ solvent molecules, but it is difficult to resolve any individual molecules. The SQUEEZE algorithm has therefore been applied.[12] There are three unique channels per unit cell. In each channel, 1018 electrons are corrected for, which corresponds to ca. 18 $CDCl_3$ molecules per channel (= 54 molecules per unit cell). This suggests a total formula $M_3TTM{\cdot}3CDCl_3$.

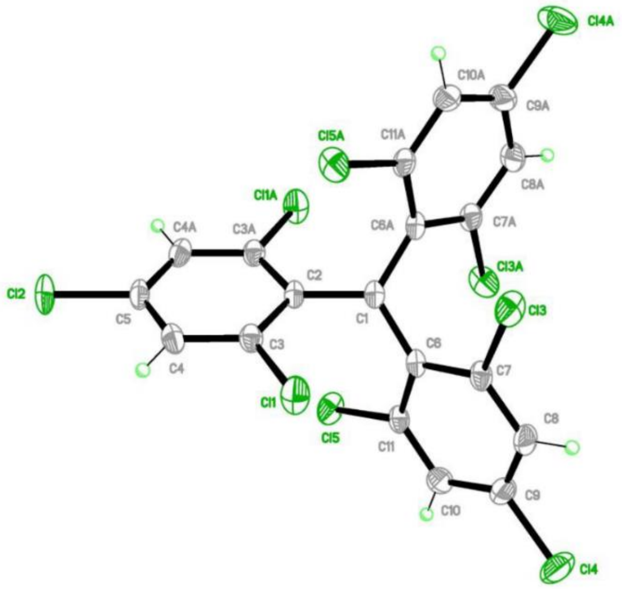

Supplementary Fig. 9 X-Ray crystal structure of TTM with atom numbering.

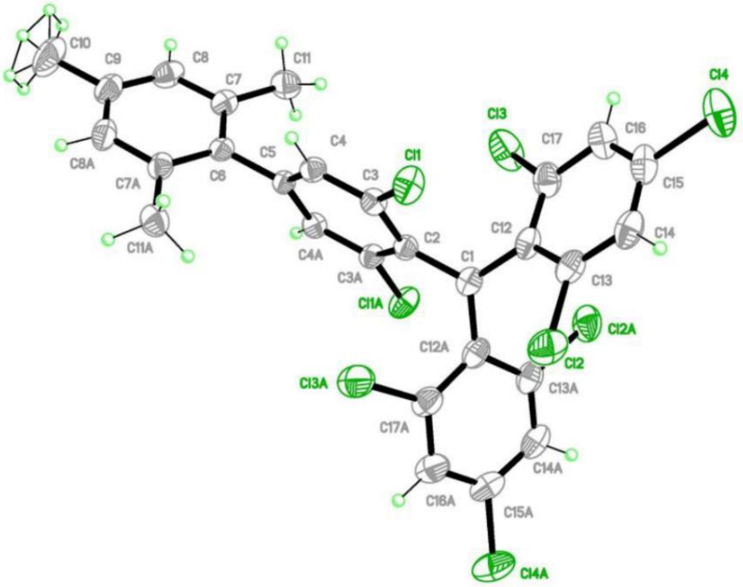

Supplementary Fig. 10 X-Ray crystal structure of M_1TTM with atom numbering.

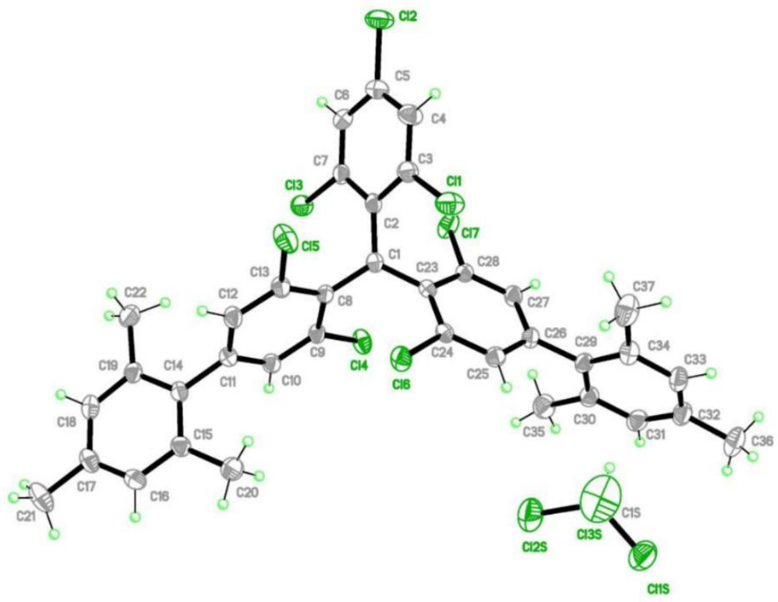

Supplementary Fig. 11 X-Ray crystal structure of M2TTM with atom numbering.

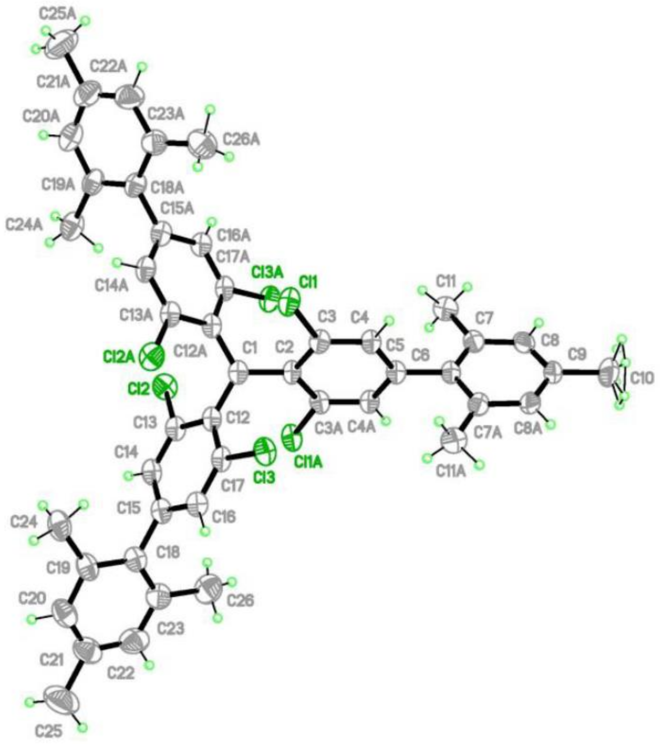

Supplementary Fig. 12 X-Ray crystal structure of M3TTM with atom numbering.

Supplementary Table 1 X-Ray crystallogrhic data of M_xTTM radicals

	**TTM**	**M_1TTM**	**M_2TTM·$CHCl_3$**	**M_3TTM [+ solvent]***
CCDC number	2195365	2195368	2195367	2195366
Cambridge data ID	HB_B2_0038	HB_B2_0034	HB_B1_0049	HB_B2_0035
Chemical formula	$C_{19}H_6Cl_9$	$C_{28}H_{17}Cl_8$	$C_{37}H_{28}Cl_7$, $CHCl_3$	$C_{46}H_{39}Cl_6$ [+ solvent]
Formula weight / g mol^{-1}	553.29	637.02	840.11	804.47
Temperature / K	180(2)	180(2)	180(2)	180(2)
Crystal system	monoclinic	tetragonal	orthorhombic	trigonal
Space group	$C2/c$	$P4_32_12$	$Pna2_1$	$R\bar{3}c$
a / Å	11.6282(7)	14.8373(5)	15.9187(4)	47.638(3)
b / Å	11.3201(6)	14.8373(5)	20.6752(5)	47.638(3)
c / Å	16.7291(10)	12.5035(6)	11.6563(3)	12.9562(12)
α / °	90	90	90	90
β / °	106.217(2)	90	90	90
γ / °	90	90	90	120
Unit-cell volume / Å^3	2114.5(2)	2752.6(2)	3836.35(17)	25463(4)
Z	4	4	4	18
Calc. density / g cm^{-3}	1.738	1.537	1.455	0.944
F (000)	1092	1284	1708	7506
Radiation type	CuKα	CuKα	CuKα	CuKα
Absorption coefficient / mm^{-1}	10.948	7.622	6.864	2.941
Crystal size / mm^3	0.28×0.22×0.06	0.18×0.10×0.06	0.25×0.25×0.05	0.18×0.10×0.08
2θ range / °	11.02–133.05	8.43–133.19	7.01–133.16	6.43–133.31
Completeness to max 2θ	0.997	1.000	0.996	0.999
No. of reflections measured	15157	35196	28474	105631
No. of independent reflections	1869	2441	6498	5020
R(int)	0.0347	0.0461	0.0381	0.0963
No. parameters/restrains	129/0	168/0	439/1	242/0
Final R1 values (I > 2σ(I))	0.0246	0.0220	0.0263	0.0442
Final wR(F^2) values (all data)	0.0627	0.0593	0.0682	0.1352
Goodness-of-fit on F^2	1.090	1.044	1.052	1.037
Largest difference peak & hole / e Å^{-3}	0.258, –0.407	0.193, –0.182	0.309, –0.406	0.286, –0.251
Flack parameter		–0.022(6)	0.025(5)	

* SQUEEZE algorithm has been applied. The correction suggests ca. 54 $CDCl_3$ per unit cell, which corresponds to a sum formula $C_{46}H_{39}Cl_6·3CDCl_3$.

Supplementary Note 4: Radical Conversion and α-Hydrogenation

General procedure for the conversion of αH precursors to π-radicals. A Wilmad® NMR tube was sealed with a Precision Seal® rubber septum cap and teflon tape. The tube was subjected to three short vacuum/Ar gas refill cycles. The αH precursor (5.0 mg, 1 equiv.) and the internal standard 1,3,5-trimethoxybenzene (1.0 mg) were added into a separate septum-sealed sample vial and the vial was subjected to three short vacuum/Ar gas refill cycles. THF-d_8 and DMSO-d_6 were bubbled with Ar gas for 5 min each and 0.25 mL and 0.75 mL were added into the vial, respectively. The mixture was transferred into the NMR tube using an Ar gas purged syringe and needle. T0 ^{1}H NMR spectrum was recorded at this point (Supplementary Fig. 13–18, black spectra). The tube was covered from light and 40% Bu_4NOH (aq, 2 equiv. bubbled with Ar gas for 15 min prior to use) was added. The tube was agitated at RT until deprotonation was complete (after addition of base, B, ^{1}H NMR spectra were recorded at different time points as shown in Supplementary Fig. 13–18, teal spectra). *p*-Chloranil (2.5 equiv.) was added into a syringe and the syringe was purged with Ar gas. In the darkness, some of the reaction mixture was taken into the syringe *via* a syringe needle to dissolve all *p*-chloranil and the mixture was pressed back into the tube. The mixture was agitated until complete oxidation to π-radical (after addition of oxidant, *p*C, ^{1}H NMR spectra were recorded and the specrtra are shown in Supplementary Fig. 13–18, red spectra). The radical was purified from the internal standard, base, oxidant, etc. as described in the experimental part (Supplementary Note 1).

Radical conversion of TTM. A common literature procedure for the conversion of various types of αHTTM derivatives to their π-radical forms involves deprotonation with a strong base like Bu_4NOH in THF solution, followed by one-electron oxidation of the corresponding carbanion with *p*-chloranil as the oxidant. We observed that carbanion of TTM was unstable in THF solution and it spontaneously oxidized to TTM radical, possibly due to trace oxygen in the degassed solvent (Supplementary Fig. 13).[13] Given that π-radicals are NMR silent, this observation raises a question about quantitative synthesis of π-radical derivatives, especially more sophisticated ones. In some cases, an unstable

carbanion form may limit the radical conversion so that the reaction does not reach completion. This is significant because the remaining αH species are often difficult to remove from the radical product using common purification techniques. We were able to somewhat stabilize the carbanion of TTM by increasing the solvent polarity (Supplementary Fig. 14), but ideally the anionic species should be so stable that it allows full synthetic control of the deprotonation–oxidation process. Mesityl substitution provides the necessary stabilization of M_xTTM anions. These anionic species and their conversions can be monitored quantitatively by NMR spectroscopy, unlike π-radicals, as discussed in the main manuscript.

Radical conversion of conjugated D–A$^{\bullet}$ derivatives. Deprotonation of αHM_2TTM-3PCz proceeded quantitatively and the carbanion was stable up to 4 h before any obvious sign of oxidation (Supplementary Fig. 18). Interestingly, M_2TTM-3PCz featured two weak and broadened ^{1}H NMR signals in the aromatic region, which we assign to the solubilizing 4-hexylphenyl group as it is less directly conjugated to the radical. Accordingly, the aliphatic 4-hexyl side chain signals were better resolved the further away they were from the conjugated backbone, whereas all signals associated with M_2TTM radical were lost (both aromatic and aliphatic).

Polyradical conversion. Conversion of αHPCzMTTM and αHPFMTTM deviated from the small molecule reactions in that the αH polymer precursors were insoluble in the highly polar solvent mixtures, making reaction monitoring by NMR impractical. However, finely dispersed polymers in DMSO/THF 3:1 (v/v) mixture dissolved completely as they were gradually deprotonated to their polyanionic forms following the addition of Bu_4NOH. Deprotonation was accompanied by a strong color change in both cases, which was utilized in qualitative reaction monitoring by UV-vis spectroscopy (Supplementary Fig. 19 and 20). Addition of *p*-chloranil caused heavy precipitation due to oxidation to neutral PCzMTTM and PFMTTM polyradicals. Following purification of the polymers, their molecular weights were largely unchanged from their αH precursors, albeit their lowest-molecular weight fractions were somewhat washed off during the radical purification step.

General procedure for the α-hydrogenation of π-radicals. A Wilmad® NMR tube was sealed with a Precision Seal® rubber septum cap and teflon tape. The tube was subjected to three short vacuum/Ar gas refill cycles. The radical (5.0 mg, 1 equiv.) and the internal standard 1,3,5-trimethoxybenzene (1.0 mg) were added into a separate septum-sealed sample vial and the vial was subjected to three short vacuum/Ar gas refill cycles. THF-d_8 and DMSO-d_6 were bubbled with Ar gas for 5 min each and 0.75 mL and 0.75 mL were added into the vial in the dark, respectively. Ultrapure water was bubbled with Ar gas for 15 min and 5 drops were added into the vial. The mixture was transferred into the NMR tube using an Ar gas purged syringe and needle. T0 ^{1}H NMR spectrum was recorded at this point (Supplementary Fig. 18 and Fig. 2 in the main manuscript). L-ascorbic acid (20 equiv.) was added into a syringe and the syringe was purged with Ar gas. In the darkness, some of the reaction mixture was taken into the syringe *via* a syringe needle to dissolve all L-ascorbic acid and the mixture was pressed back into the tube. The mixture was agitated at RT. After no longer significant change in the ^{1}H NMR spectrum was observed, 1 drop of 1 M NaOH (aq) was added to deprotonate the remaining L-ascorbic acid (see the overlapping ^{1}H NMR signals in the aromatic region in Supplementary Fig. 22), so as to better resolve the aromatic signals of the target compound. This also confirms that the weak additional signals in the baselines of α-hydrogenated products are not from reaction intermediates or by-products, but they originate from L-ascorbic acid. ^{1}H NMR spectra are shown after adding both L-ascorbic acid and base (AA, Supplementary Fig. 18 and Fig. 2 in the main manuscript).

α-Hydrogenation of conjugated D–A$^•$ derivatives. The α-hydrogenation of M_2TTM-3PCz followed the same protocol as that of the M_xTTM series discussed in the main manuscript, albeit even slower, which can be understood as further stabilization of the radical by the D–A$^•$ structure and its extended conjugation. In this case ^{1}H NMR spectra were recorded after 5 weeks of reaction, again confirming that the α-hydrogenated product was the same species as the corresponding αH precursor (Supplementary Fig. 18).

Preparation of deuterated L-ascorbic acid (AA-d_4). L-ascorbic acid (100 mg) was dissolved in D_2O (1 mL). The hydrogen/deuterium exchange was confirmed by NMR giving AA-d_4 with full conversion directly after dissolving. To remove any hydrogen source (H_2O, etc.), all solvent was removed under reduced pressure and the solids were dried under vacuum. AA-d_4 was collected as white solid, and it was stored under Ar gas and used without further purification.

General procedure for the α-deuteration of polyradicals. A Wilmad® NMR tube was sealed with a Precision Seal® rubber septum cap and teflon tape. The tube was subjected to three short vacuum/Ar gas refill cycles. The polyradical (5.0 mg, 1 repeating unit equiv.) was added into a separate septum-sealed sample vial and the vial was subjected to three short vacuum/Ar gas refill cycles. THF-d_8 was bubbled with Ar gas for 5 min and 1 mL was added into the vial in the dark. After the polymer was dissolved, the mixture was transferred into the NMR tube using an Ar gas purged syringe and needle. T0 ^{1}H NMR spectrum was recorded at this point (Supplementary Fig. 21). Freshly prepared 1 M solution of AA-d_4 in D_2O (20 equiv. per repeating unit, prepared by dissolving AA-d_4 in D_2O, bubbled with Ar gas for 15 min prior to use) was added into the tube. The mixture was agitated at RT. ^{1}H NMR spectra are shown without adding base due to the slow reaction (see below).

α-Deuteration of polyradicals. α-Deuteration was attempted to quantify the radical content in the polymers by their αH/αD ratio. Deuterated L-ascorbic acid in D_2O was used because both ascorbic acid and water may function coordinately as the H/D source, while THF-d_8 was used as the solvent due to the limited solubility of the polymers and because the reverse reactions did not proceed *via* soluble polyanionic forms (see the discussion in the main manuscript). After 8 weeks reaction, the ^{1}H NMR spectra of the polymers show vanishingly small amount of α-deuterated product (Supplementary Fig. 21), as the extention of conjugation from M_xTTM over M_2TTM-3PCz finally to PFMTTM and PCzMTTM polyradicals provides systematic stabilization of TTM radical.

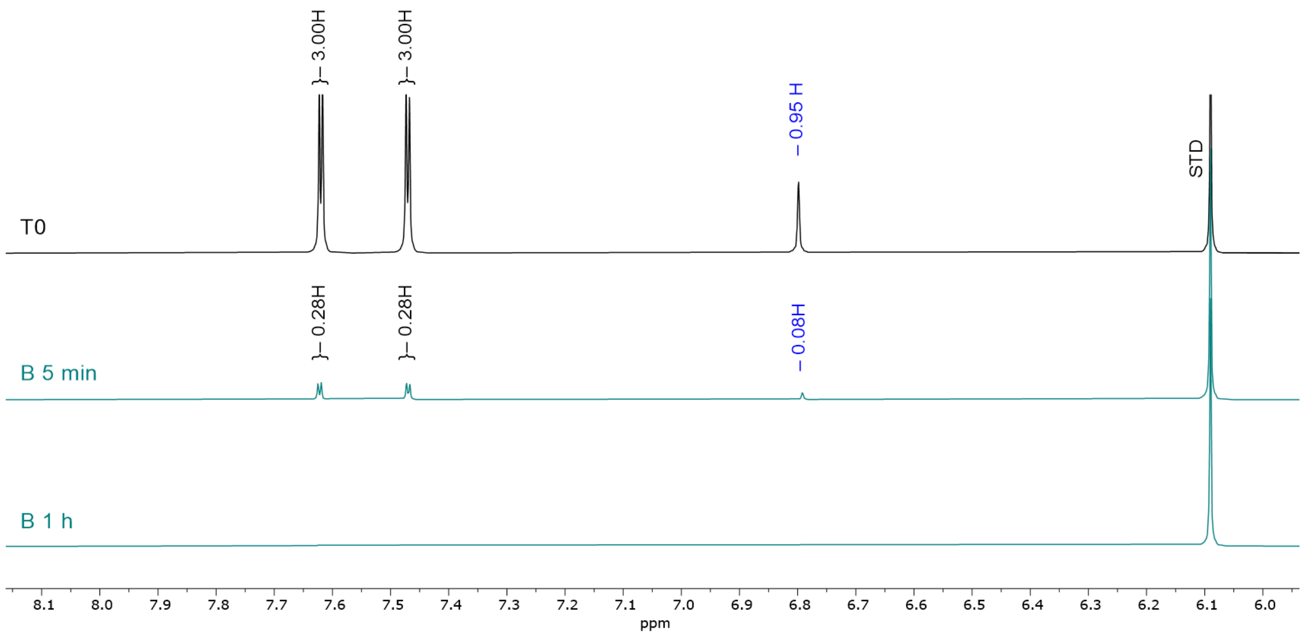

Supplementary Fig. 13 Deprotonation of αHTTM monitored by NMR (THF-*d*$_8$). ^{1}H NMR spectra of the αH precursor before deprotonation (black line), and 5 min and 1 h after addition of base showing spontaneous oxidation to NMR silent TTM radical (teal lines), as indicated in the legend. The peak integrals illustrate rapid deprotonation–oxidation process. No carbanion species was observed in the reaction. All spectra have been referenced against 1,3,5-trimethoxybenzene (^{1}H, 6.09 ppm) as the internal standard.

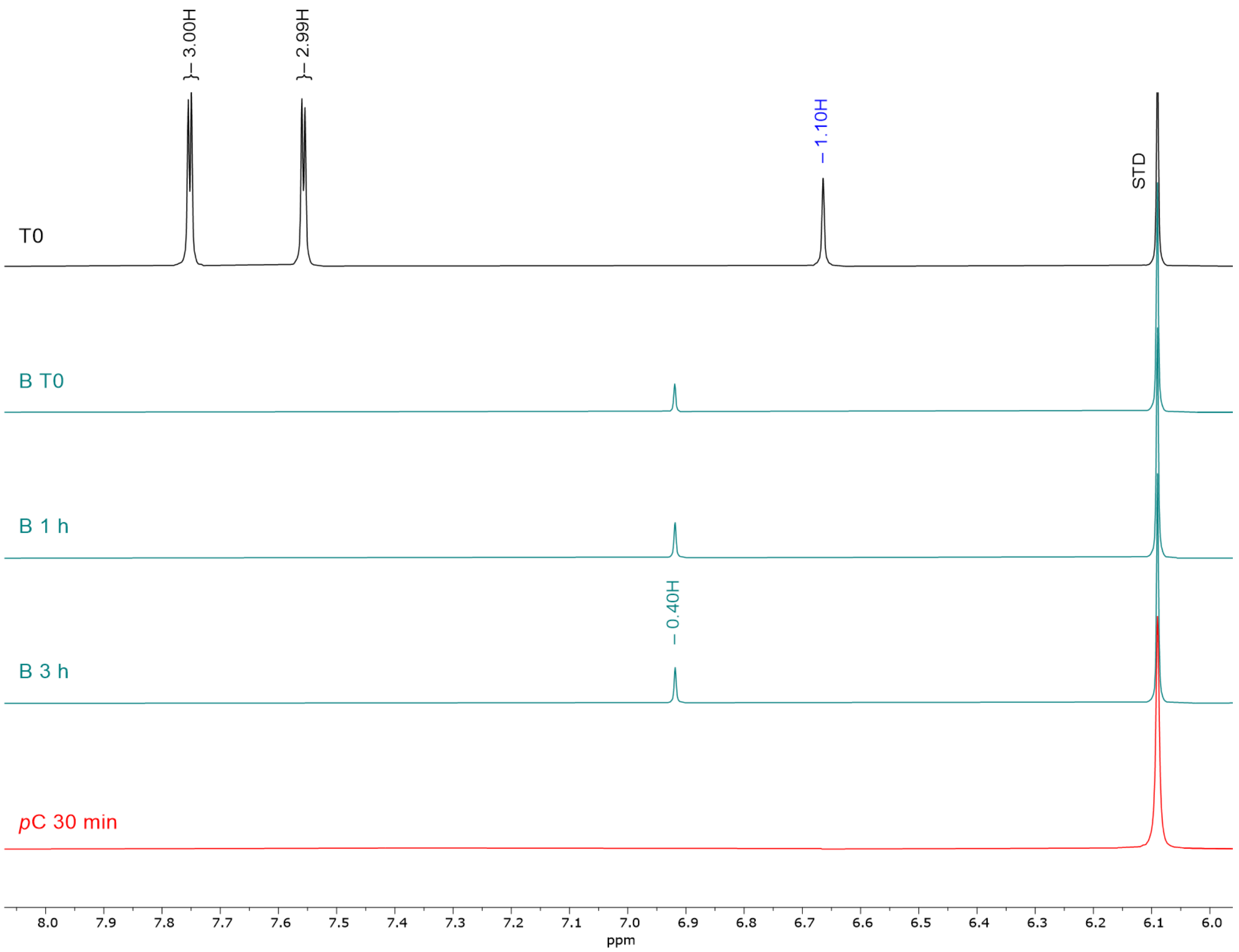

Supplementary Fig. 14 Radical conversion of αHTTM monitored by NMR. Deprotonation of the αH precursor (40% Bu_4NOH, aq, 2 equiv.) followed by one-electron oxidation (*p*-chloranil, 2.5 equiv.) in DMSO-d_6/THF-d_8 3:1 (v/v). ^{1}H NMR spectra of the αH precursor before deprotonation (black line), at T0 and 1 h and 3 h after addition of base showing formation of the carbanion (teal lines) and 30 min after addition of oxidant showing formation of the radical (red line), as indicated in the legend. "B T0" stands for spectrum taken directly after adding the base. Selected peak integrals are shown to illustrate complete deprotonation and formation of anionic species within 3 hours. All spectra have been referenced against 1,3,5-trimethoxybenzene (^{1}H, 6.09 ppm) as the internal standard.

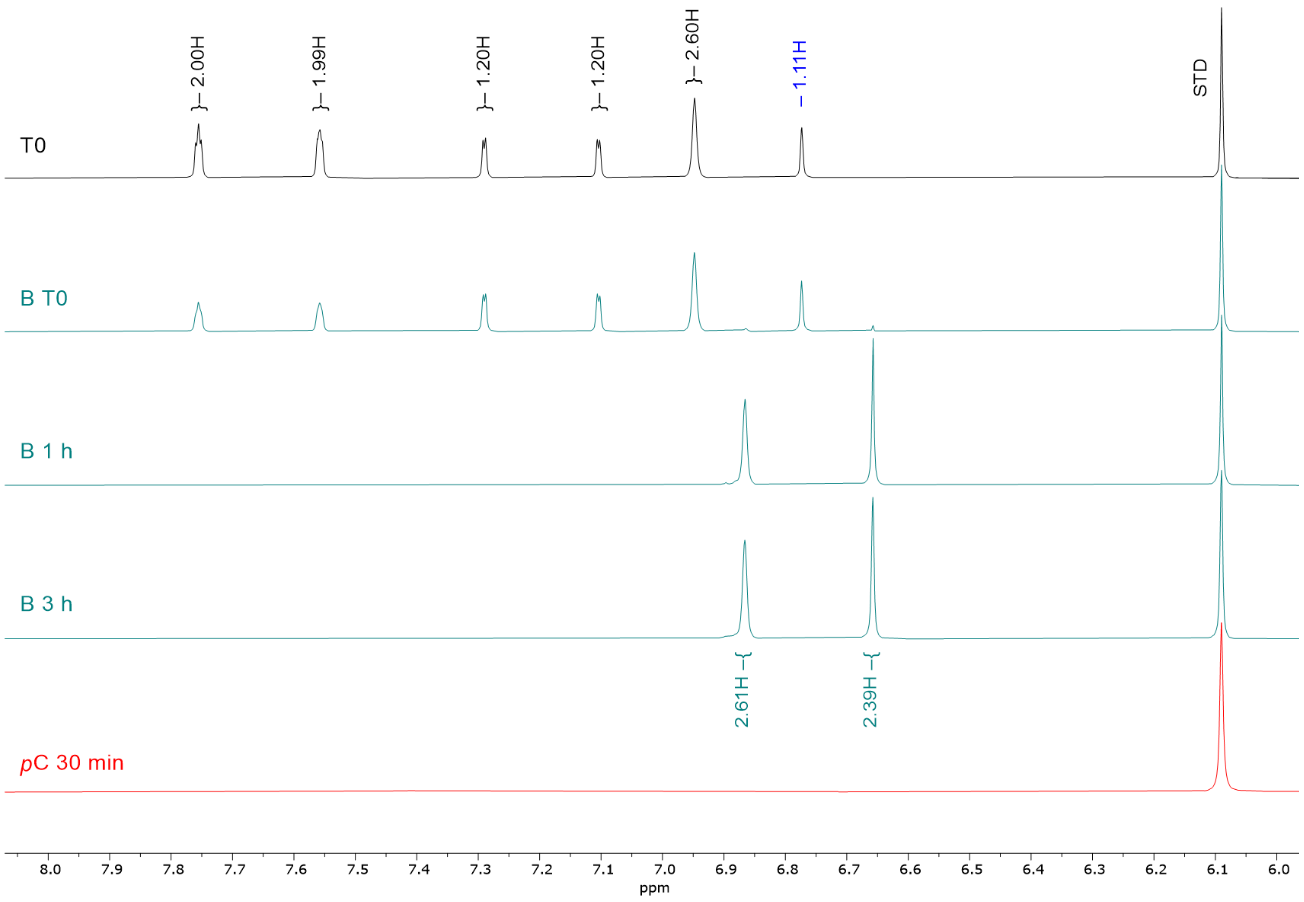

Supplementary Fig. 15 Radical conversion of αHM$_1$TTM monitored by NMR. Deprotonation of the αH precursor (40% Bu_4NOH, aq, 2 equiv.) followed by one-electron oxidation (*p*-chloranil, 2.5 equiv.) in DMSO-d_6/THF-d_8 3:1 (v/v). ^{1}H NMR spectra of the αH precursor before deprotonation (black line), at T0 and 1 h and 3 h after addition of base showing formation of the carbanion (teal lines) and 30 min after addition of oxidant showing formation of the radical (red line), as indicated in the legend. "B T0" stands for spectrum taken directly after adding the base. Selected peak integrals are shown to illustrate complete deprotonation and formation of anionic species within 3 hours. All spectra have been referenced against 1,3,5-trimethoxybenzene (^{1}H, 6.09 ppm) as the internal standard.

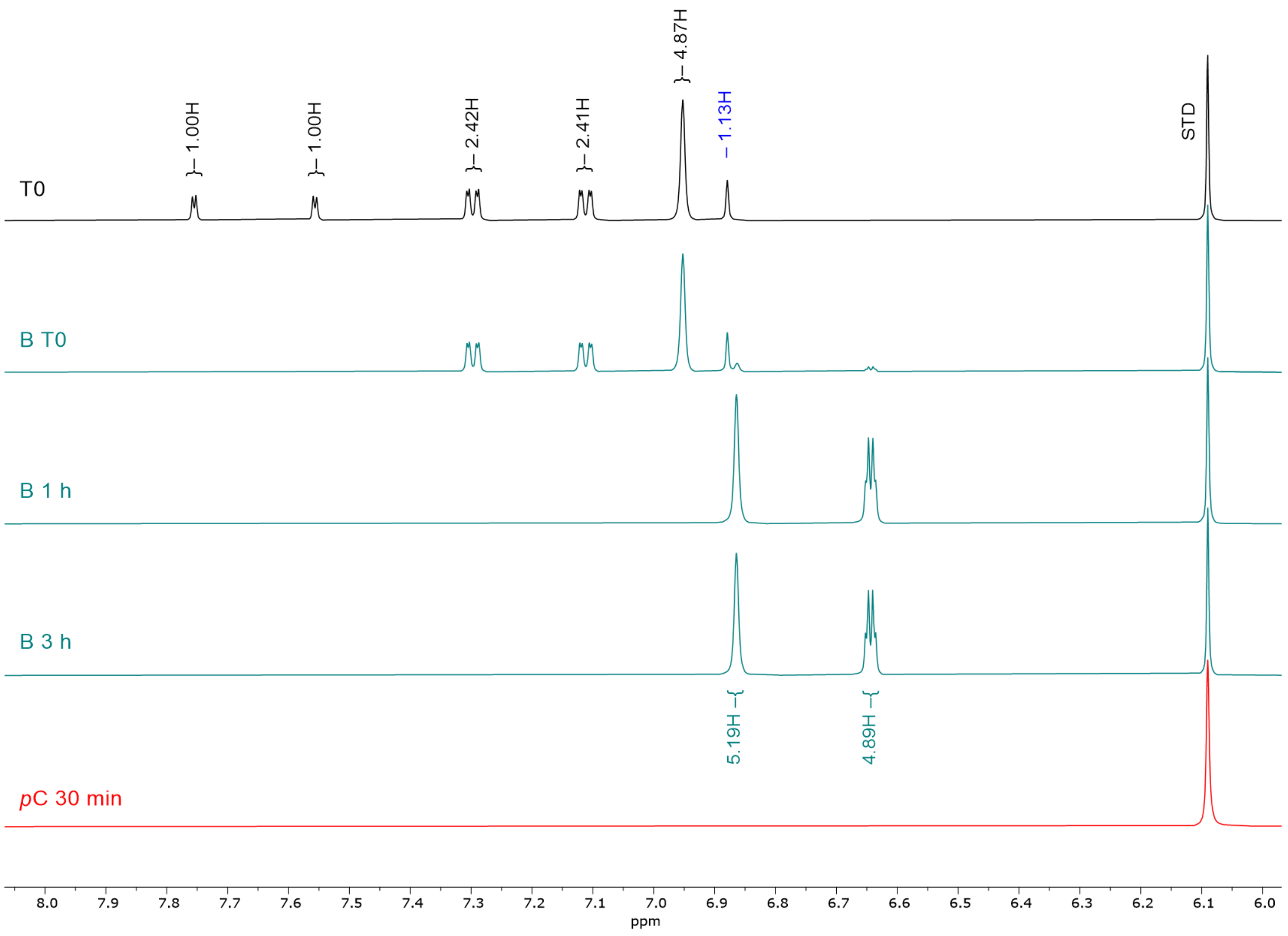

Supplementary Fig. 16 Radical conversion of αHM$_2$TTM monitored by NMR. Deprotonation of the αH precursor (40% Bu_4NOH, aq, 2 equiv.) followed by one-electron oxidation (*p*-chloranil, 2.5 equiv.) in DMSO-d_6/THF-d_8 3:1 (v/v). ^{1}H NMR spectra of the αH precursor before deprotonation (black line), at T0 and 1 h and 3 h after addition of base showing formation of the carbanion (teal lines) and 30 min after addition of oxidant showing formation of the radical (red line), as indicated in the legend. “B T0” stands for spectrum taken directly after adding the base. Selected peak integrals are shown to illustrate complete deprotonation and formation of anionic species within 3 hours. All spectra have been referenced against 1,3,5-trimethoxybenzene (^{1}H, 6.09 ppm) as the internal standard.

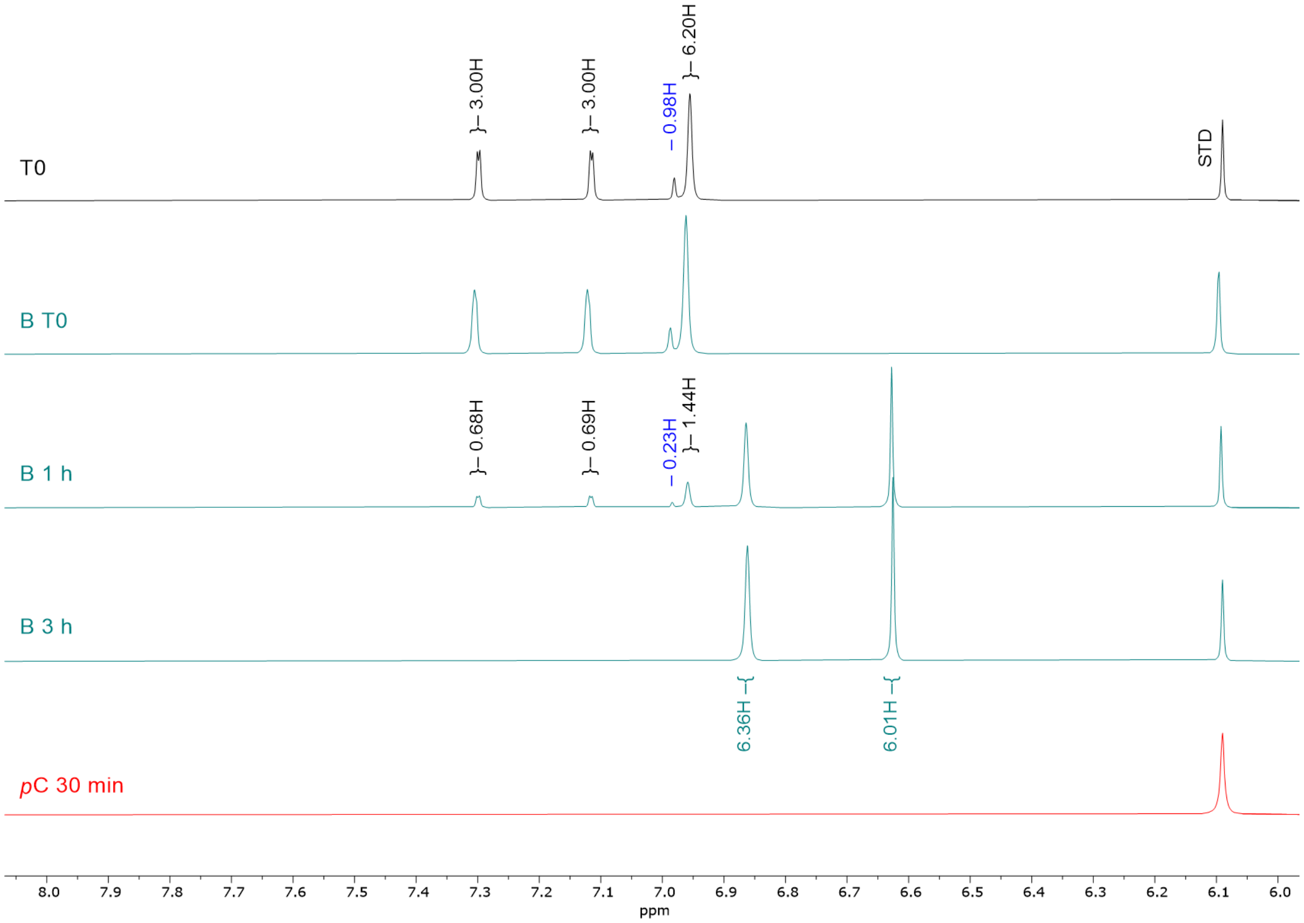

Supplementary Fig. 17 Radical conversion of αHM$_3$TTM monitored by NMR. Deprotonation of the αH precursor (40% Bu_4NOH, aq, 2 equiv.) followed by one-electron oxidation (*p*-chloranil, 2.5 equiv.) in DMSO-*d*$_6$/THF-*d*$_8$ 3:1 (v/v). ^{1}H NMR spectra of the αH precursor before deprotonation (black line), at T0 and 1 h and 3 h after addition of base showing formation of the carbanion (teal lines) and 30 min after addition of oxidant showing formation of the radical (red line), as indicated in the legend. "B T0" stands for spectrum taken directly after adding the base. Selected peak integrals are shown to illustrate complete deprotonation and formation of anionic species within 3 hours. All spectra have been referenced against 1,3,5-trimethoxybenzene (^{1}H, 6.09 ppm) as the internal standard.

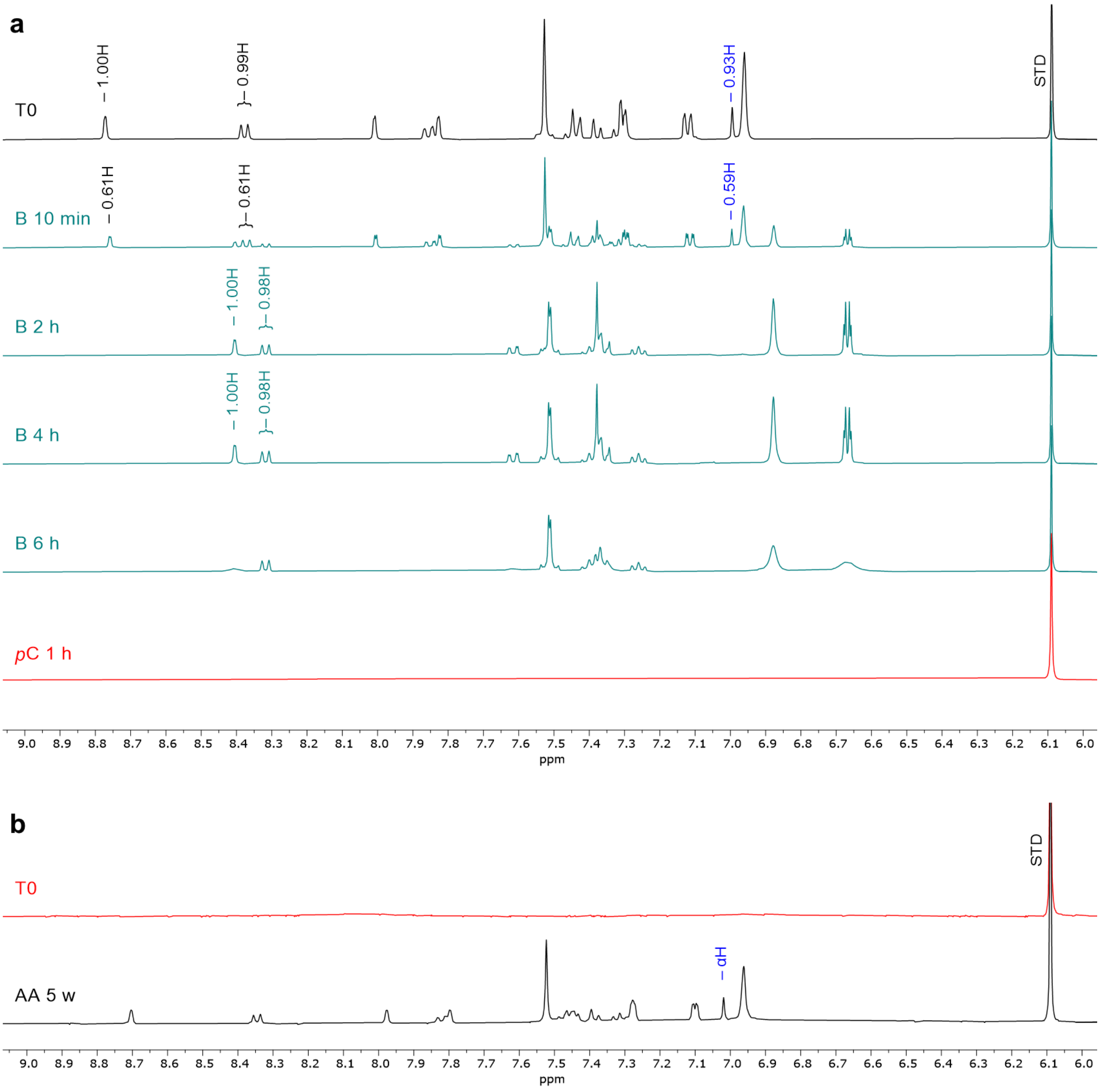

Supplementary Fig. 18 Radical conversion and α-hydrogenation of M_2TTM-3PCz monitored by NMR. a, Deprotonation of the αH precursor (40% Bu_4NOH, aq, 2 equiv.) followed by one-electron oxidation (*p*-chloranil, 2.5 equiv.) in DMSO-d_6/THF-d_8 3:1 (v/v). ^{1}H NMR spectra of the αH precursor before deprotonation (black line), 10 min, 2 h, 4 h and 6 h after addition of base showing formation of the carbanion (teal lines) and 1 h after addition of oxidant showing formation of the radical (red line), as indicated in the legend. Selected peak integrals are shown to illustrate complete deprotonation and formation of anionic species within 4 hours. **b**, α-Hydrogenation of the radical (L-ascorbic acid, aq, 20 equiv.) in DMSO-d_6/THF-d_8 1:1 (v/v). ^{1}H NMR spectra of the radical before α-hydrogenation (red line) and 5 weeks after addition of L-ascorbic acid showing slow formation of the α-hydrogenated

product (black line), as indicated in the legend. No carbanion species was observed in the reverse reaction. All spectra have been referenced against 1,3,5-trimethoxybenzene (^{1}H, 6.09 ppm) as the internal standard.

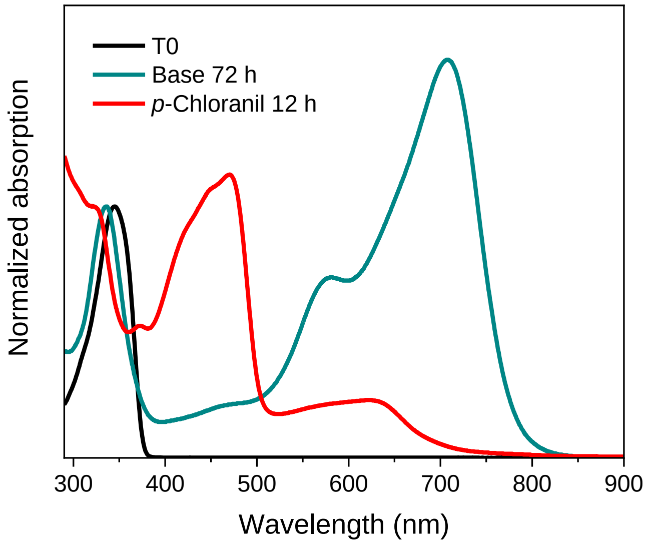

Supplementary Fig. 19 Radical conversion of αHPFMTTM monitored by UV-vis spectroscopy in THF solution. Deprotonation of αH polymer precursor (40% Bu_4NOH, aq, 4 equiv. per repeating unit) followed by one-electron oxidation (*p*-chloranil, 2.5 equiv. per repeating unit) in DMSO/THF 3:1 (v/v). The αH polymer precursor before deprotonation (black line), 72 h after addition of base showing formation of stable polyanion (teal line) and 12 h after addition of oxidant showing formation of the polyradical (red line), as indicated in the legend. The spectra are normalized relative to the UV band peaking at ca. 350 nm. In the polyradical spectrum (red line) the absorption band below 300 nm belongs to *p*-chloranil.

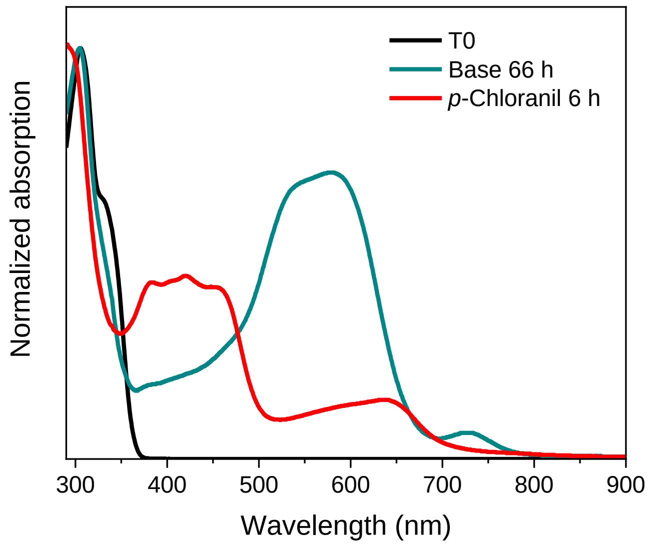

Supplementary Fig. 20 Radical conversion of αHPCzMTTM monitored by UV-vis spectroscopy in THF solution. Deprotonation of αH polymer precursor (40% Bu_4NOH, aq, 3 equiv. per repeating unit) followed by one-electron oxidation (*p*-chloranil, 2.5 equiv. per repeating unit) in DMSO/THF 3:1 (v/v). The αH polymer precursor before deprotonation (black line), 66 h after addition of base showing formation of stable polyanion (teal line) and 6 h after addition of oxidant showing formation of the polyradical (red line), as indicated in the legend. The spectra are normalized relative to the UV band peaking at ca. 300 nm. In the polyradical spectrum (red line) the absorption band below 300 nm belongs to *p*-chloranil.

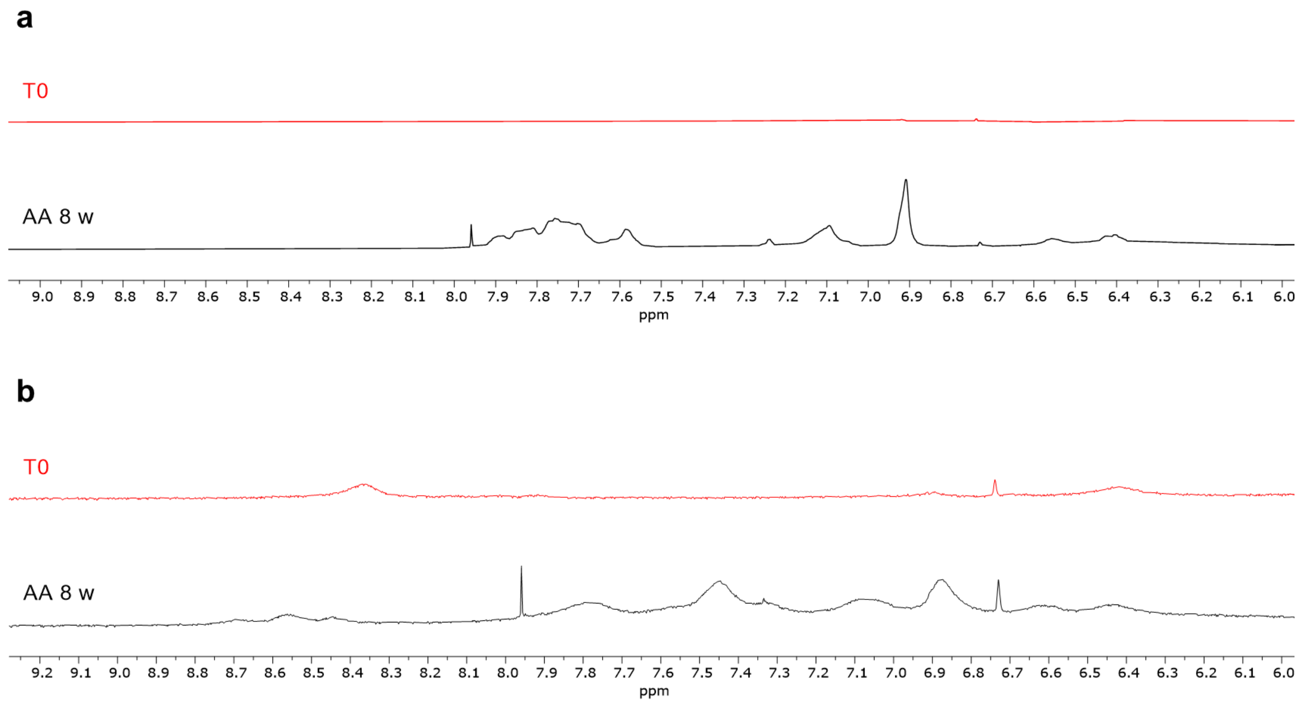

Supplementary Fig. 21 α-Deuteration of the polyradicals monitored by NMR. a, PFMTTM, **b**, PCzMTTM (AA-d_4, 20 equiv. per repeating unit, in THF-d_8). ^{1}H NMR spectra of the polymers before α-deuteration (red lines) and 8 weeks after addition of AA-d_4 showing slow formation of the α-deuterated polymers (black lines), as indicated in the legend. The emerging signals are weak due to the slow reverse reaction. All spectra have been referenced against THF-d_8 solvent peak (^{1}H, 3.58 ppm).

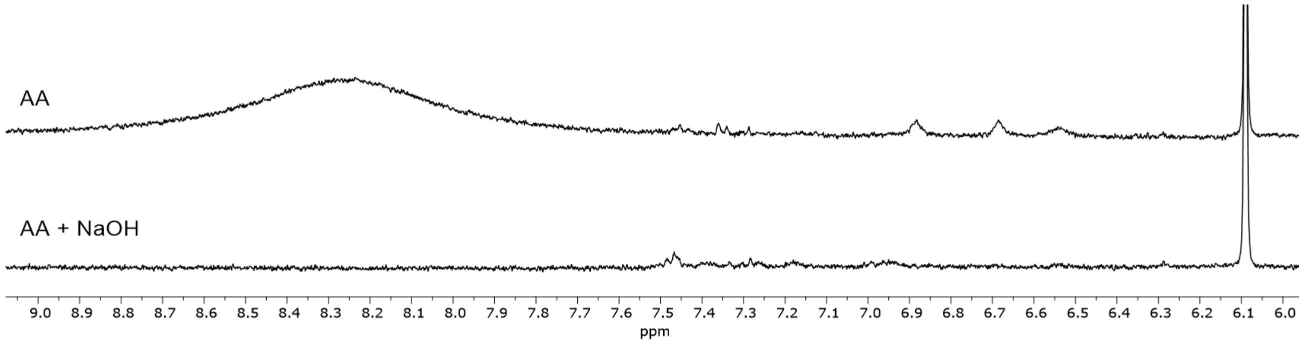

Supplementary Fig. 22 L-ascorbic acid (aq) in DMSO-d_6/THF-d_8 1:1 (v/v). ^{1}H NMR spectra before (top) and after (bottom) adding 1 drop of 1 M NaOH (aq), as indicated in the legend. The spectra have been referenced against 1,3,5-trimethoxybenzene (^{1}H, 6.09 ppm) as the internal standard.

Supplementary Note 5: DFT Calculations

Electronic structures of M_xTTM radicals. The influence of the mesityl substituents on the frontier molecular orbitals energy ordering of TTM was evaluated by unrestricted DFT calculations. The mesityls dominated contribution to the highest doubly occupied molecular orbitals (HOMO, HOMO–1, etc.) shallowing the SOMO energy involving the unpaired electron (see below). This is expected due to electronic effect of the mesityl groups (replacement of the electron-withdrawing *p*-chlorines by the mesityls and blocking of relevant orbital distribution beyond the *p*-positions), but it also contrasts with previously reported D–A$^{\bullet}$ type radical derivatives where the radical is stabilized by deepening the SOMO energy (Supplementary Fig. 1).

Radiative decay rate of M_3TTM. To further analyse the emission nature of M_3TTM, we scanned models based on its optimized D_1 state geometry with manually set dihedral angle between one of the dichlorobenzene ligands and its corresponding mesityl group from 0 to 90° while the other two dihedrals were fixed at 90°. The radiative decay rate was estimated by the Einstein equation[14,15] with calculated excited state energy levels and relevant oscillator strengths (see the Methods Section in the main manuscript).[16,17] As shown in Supplementary Fig. 23, the radiative decay rates were about an order of magnitude lower than those of CT type radical emitters,[15,18] in line with the experimental decay rates (Table 1 in the main manuscript), due to the excited state transition dipole moment cancellation of the highly symmetric TTM core. The radiative decay rates were higher when the geometry was less symmetric, which is observed as relaxation from a darker D_0 geometry to a brighter D_1 geometry.

Charge transfer features of D–A$^{\bullet}$ backbones. As discussed in the main manuscript, substitution of the chlorophenyl ligand with an electron-rich donor unit can break the molecular orbital symmetry and strengthen the HOMO–SOMO interacation,[19] which can be intuitively regarded as interaction between the donor and the radical centre bridged by the chlorophenyl ligand. Therefore, the radical involved

D_1 state of mesityl substituted D–A$^•$ derivatives like M_2TTM-3PCz can be described as a hybrid of short-range CT character delocalized within the M_xTTM moiety and long-range CT character assigned to the D–A$^•$ interaction. The donor unit does not significantly enhance the $D_0 \rightarrow D_1$ excitation as the long-range CT brings in very small oscillator strength due to small overlap of relevant molecular orbitals. The emission, on the other hand, can be strenghtened by two factors: enhanced short-range CT arising from molecular symmetry breaking following D_1 relaxation and 2) long-range CT dominated by the intensity borrowing effect reported previously.[19] This is observed experimentally as doubling of PLQE for the M_xTTM derived TTM-3PCz radical (see the main manuscript).

Supplementary Table 2 Energy levels of frontier molecular orbitals of trityl radicals

Radical	HOMOα	HOMOβ	SOMO	SUMO	LUMOα	LUMOβ
TTM	–6.98	–6.96	–5.65	–3.39	–1.46	–1.35
M_1TTM	–6.56	–6.56	–5.52	–3.26	–1.35	–1.25
M_2TTM	–6.51	–6.51	–5.41	–3.14	–1.21	–1.11
M_3TTM	–6.46	–6.46	–5.29	–3.01	–1.05	–0.94
PTM	–7.10	–7.06	–6.11	–3.79	–1.91	–1.83
PyBTM	–7.05	–7.01	–5.88	–3.59	–1.67	–1.57
bisPyTM	–7.20	–7.15	–6.14	–3.79	–1.84	–1.74
trisPyM	–7.34	–7.30	–6.44	–3.99	–1.94	–1.85

Supplementary Table 3 Energy levels of frontier molecular orbitals of CBP and αH precursors

Structure	HOMO	LUMO
CBP	–5.52	–1.68
αHTTM	–6.87	–1.39
αHM_1TTM	–6.54	–1.29
αHM_2TTM	–6.47	–1.16
αHM_3TTM	–6.42	–0.97
αHPTM	–7.07	–1.86
αHPyBTM	–7.01	–1.64
αHbisPyTM	–7.12	–1.78
αHtrisPyM	–7.29	–1.87

TTM

	α	β
LUMO	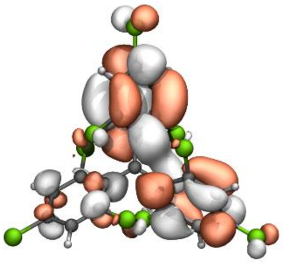 138a = –1.46 eV Occ.:0	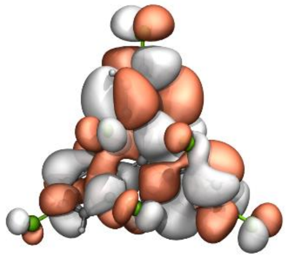 138b = –1.35 eV Occ.:0
SOMO	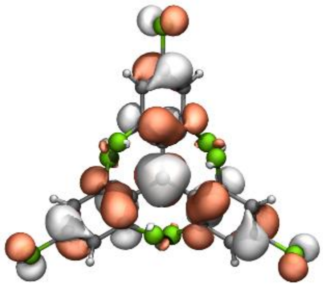 137a = –5.65 eV Occ.:1	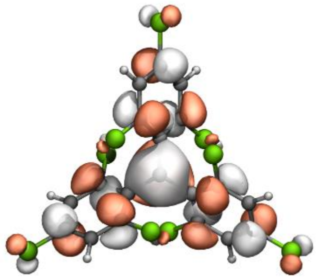 137b = –3.39 eV Occ.:0
HOMO	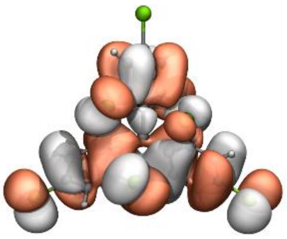 136a = –6.98 eV Occ.:1	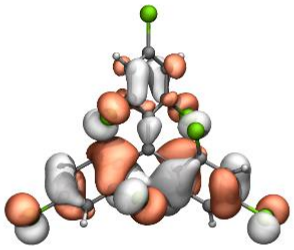 136b = –6.96 eV Occ.:1
HOMO–1	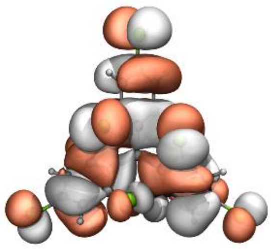 135a = –6.98 eV Occ.:1	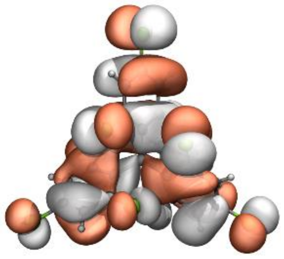 135b = –6.96 eV Occ.:1

M$_1$TTM

	α	β
LUMO	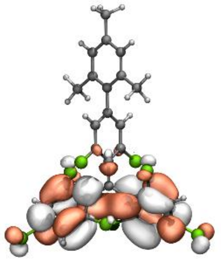 162a = –1.35 eV Occ.:0	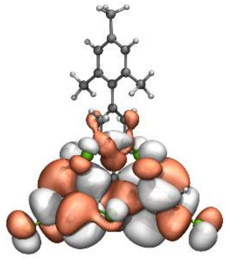 162b = –1.25 eV Occ.:0
SOMO	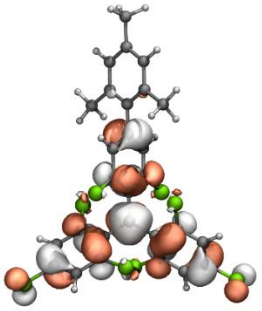 161a = –5.52 eV Occ.:1	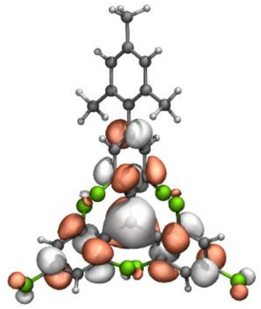 161b = –3.26 eV Occ.:0
HOMO	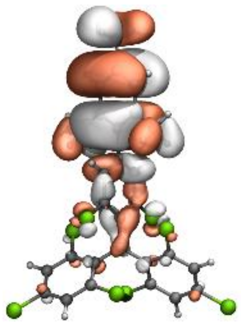 160a = –6.56 eV Occ.:1	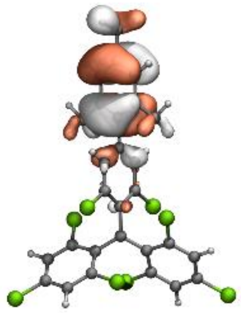 160b = –6.56 eV Occ.:1
HOMO–1	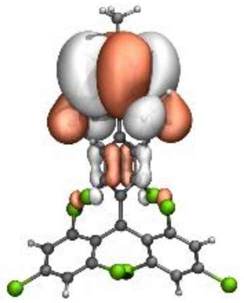 159a = –6.66 eV Occ.:1	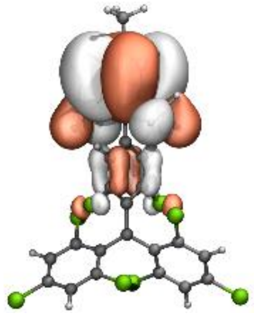 159b = –6.66 eV Occ.:1
HOMO–2	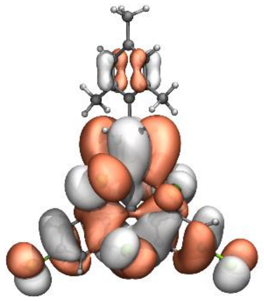 158a = –6.84 eV Occ.:1	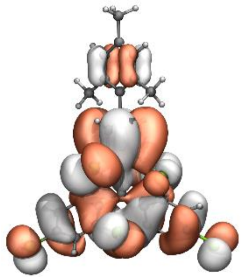 158b = –6.81 eV Occ.:1

M_2TTM

	α	β
LUMO	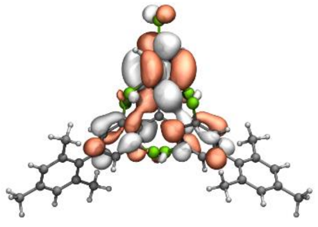 186a = –1.21 eV Occ.:0	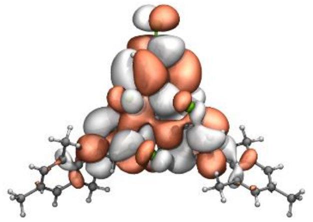 186b = –1.11 eV Occ.:0
SOMO	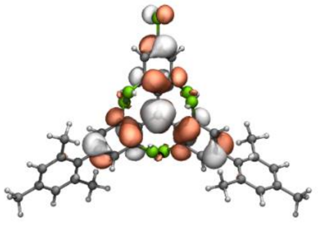 185a = –5.41 eV Occ.:1	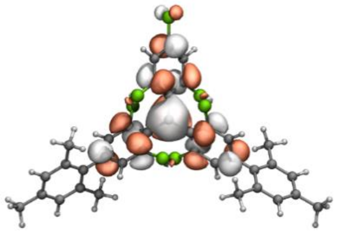 185b = –3.14 eV Occ.:0
HOMO	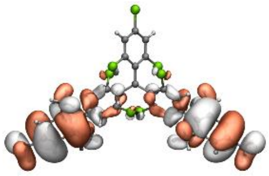 184a = –6.51 eV Occ.:1	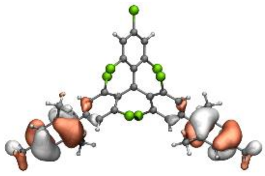 184b = –6.51 eV Occ.:1
HOMO–1	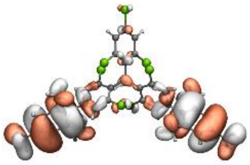 183a = –6.52 eV Occ.:1	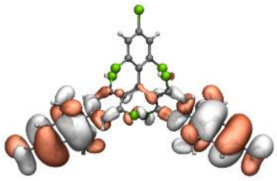 183b = –6.51 eV Occ.:1
HOMO–2	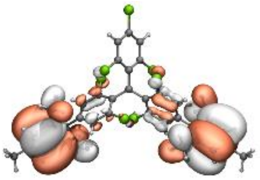 182a = –6.61 eV Occ.:1	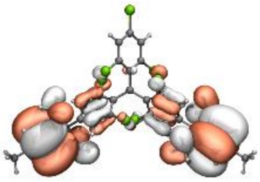 182b = –6.61 eV Occ.:1
HOMO–3	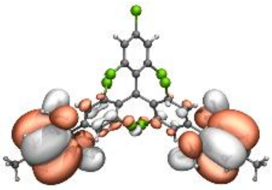 181a = –6.61 eV Occ.:1	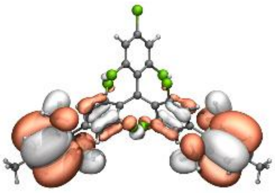 181b = –6.61 eV Occ.:1
HOMO–4	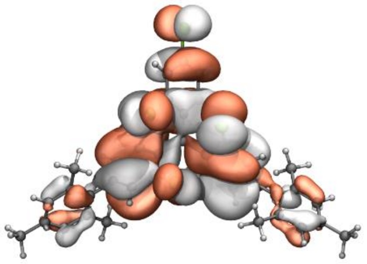 180a = –6.74 eV Occ.:1	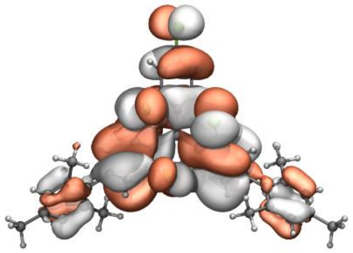 180b = –6.71 eV Occ.:1

M_3TTM

	α	β
LUMO	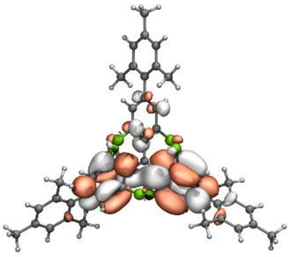<br>210a = –1.05 eV Occ.:0	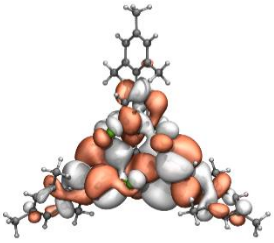<br>210b = –0.94 eV Occ.:0
SOMO	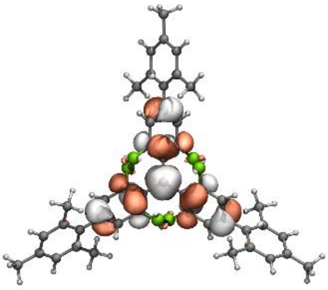<br>209a = –5.29 eV Occ.:1	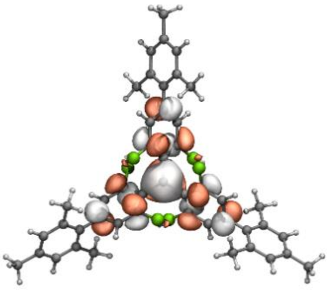<br>209b = –3.01 eV Occ.:0
HOMO	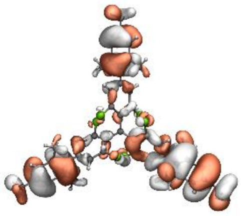<br>208a = –6.46 eV Occ.:1	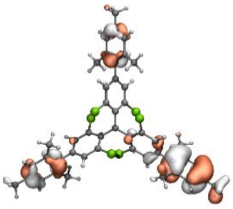<br>208b = –6.46 eV Occ.:1
HOMO–1	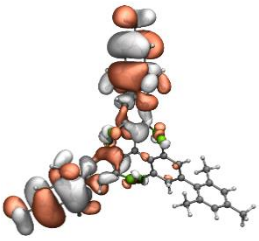<br>207a = –6.46 eV Occ.:1	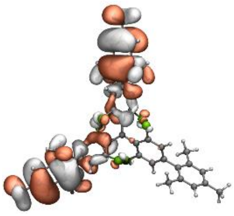<br>207b = –6.46 eV Occ.:1
HOMO–2	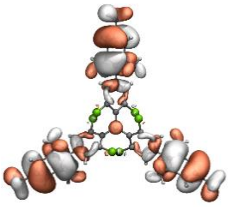<br>206a = –6.48 eV Occ.:1	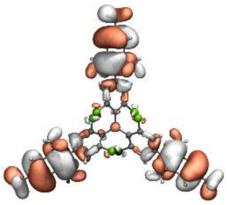<br>206b = –6.47 eV Occ.:1
HOMO–3	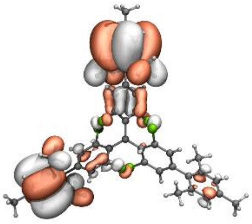<br>205a = –6.56 eV Occ.:1	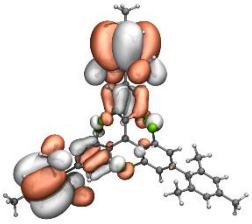<br>205b = –6.56 eV Occ.:1

HOMO–4	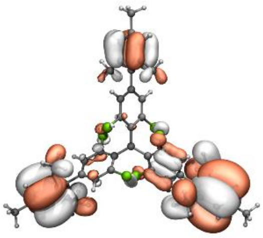<br>204a = –6.57 eV Occ.:1	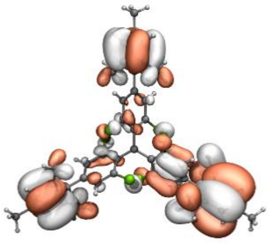<br>204b = –6.56 eV Occ.:1
HOMO–5	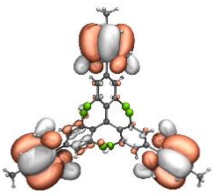<br>203a = –6.57 eV Occ.:1	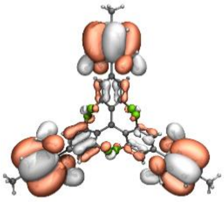<br>203b = –6.56 eV Occ.:1
HOMO–6	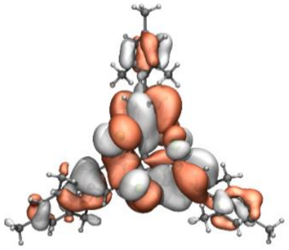<br>202a = –6.67 eV Occ.:1	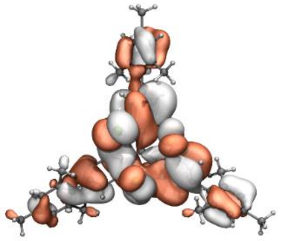<br>202b = –6.64 eV Occ.:1

PTM

	**α**	**β**
LUMO	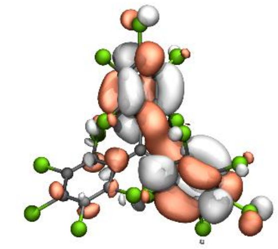<br>186a = –1.91 eV Occ.:0	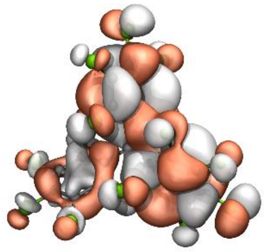<br>186b = –1.83 eV Occ.:0
SOMO	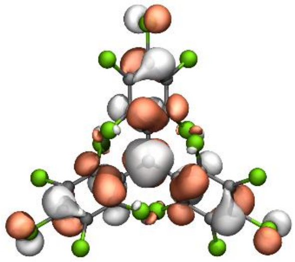<br>185a = –6.11 eV Occ.:1	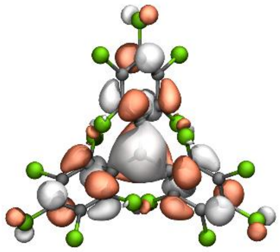<br>185b = –3.79 eV Occ.:0
HOMO	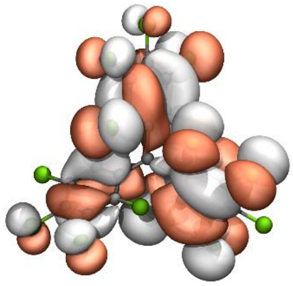<br>184a = –7.10 eV Occ.:1	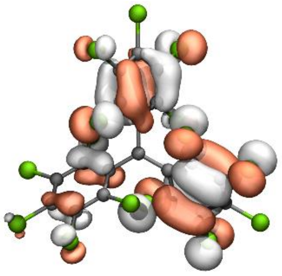<br>184b = –7.06 eV Occ.:1
HOMO–1	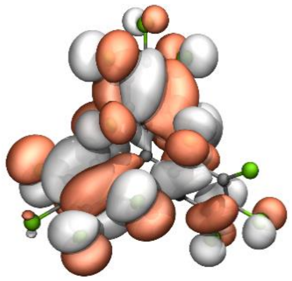<br>183a = –7.10 eV Occ.:1	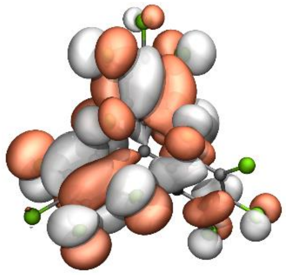<br>183b = –7.06 eV Occ.:1

PyBTM

	α	β
LUMO	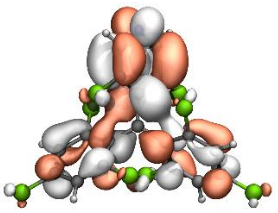 130a = –1.67 eV Occ.:0	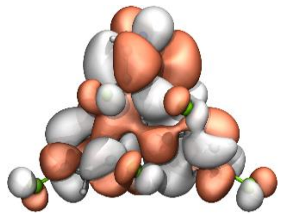 130b = –1.57 eV Occ.:0
SOMO	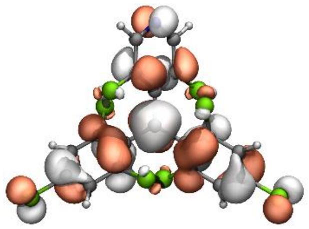 129a = –5.88 eV Occ.:1	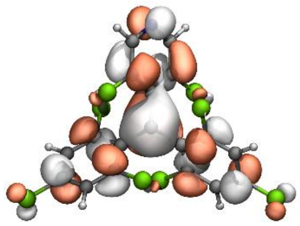 129b = –3.59 eV Occ.:0
HOMO	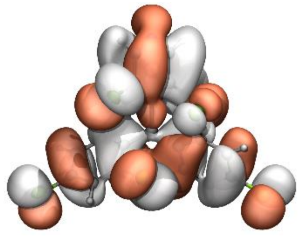 128a = –7.05 eV Occ.:1	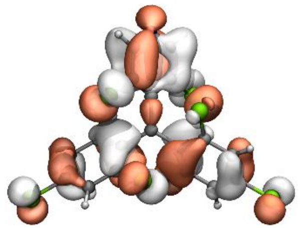 128b = –7.01 eV Occ.:1
HOMO–1	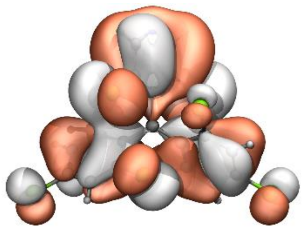 127a = –7.20 eV Occ.:1	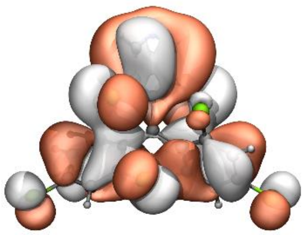 127b = –7.15 eV Occ.:1

bisPyTM

	α	β
LUMO	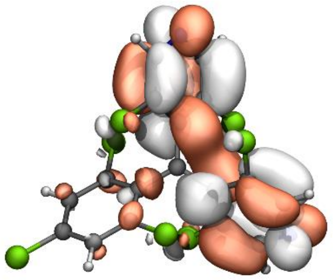 122a = –1.84 eV Occ.:0	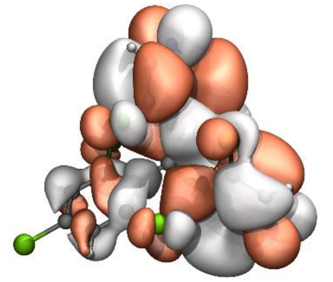 122b = –1.74 eV Occ.:0
SOMO	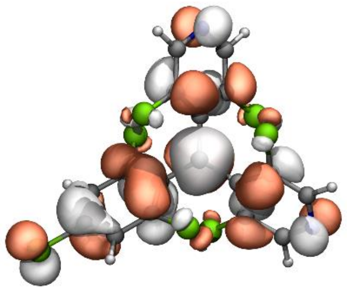 121a = –6.14 eV Occ.:1	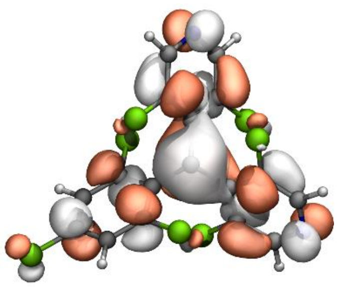 121b = –3.79 eV Occ.:0
HOMO	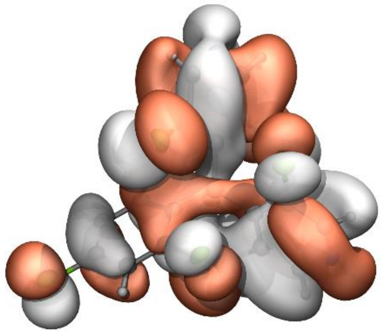 120a = –7.20 eV Occ.:1	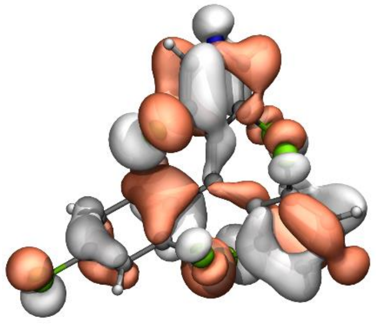 120b = –7.15 eV Occ.:1
HOMO–1	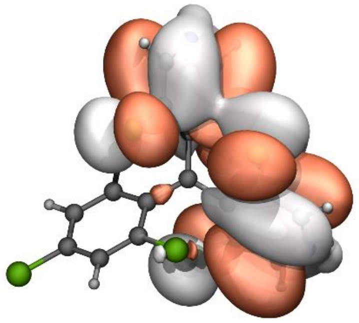 119a = –7.23 eV Occ.:1	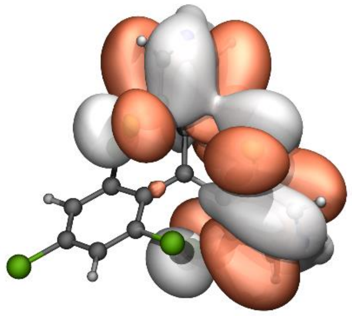 119b = –7.19 eV Occ.:1

trisPyM

	**α**	**β**
LUMO	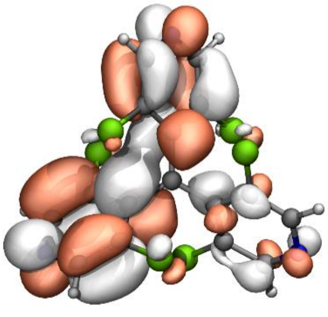<br>114a = –1.94 eV Occ.:0	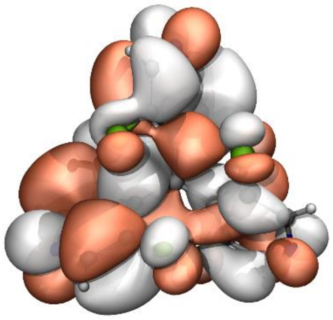<br>114b = –1.85 eV Occ.:0
SOMO	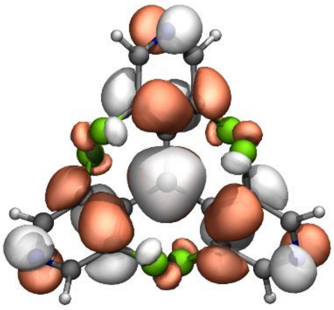<br>113a = –6.44 eV Occ.:1	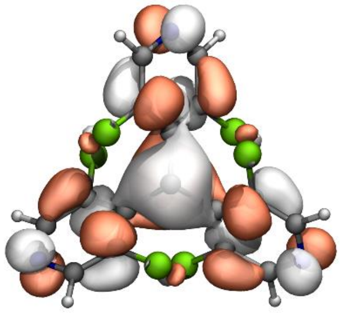<br>113b = –3.99 eV Occ.:0
HOMO	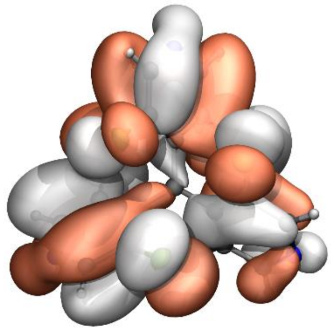<br>112a = –7.34 eV Occ.:1	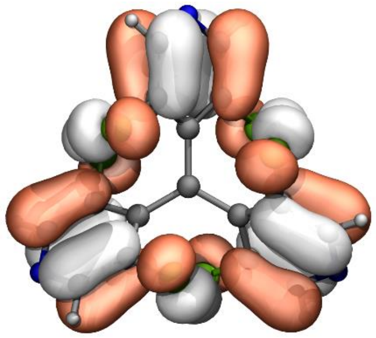<br>112b = –7.30 eV Occ.:1
HOMO–1	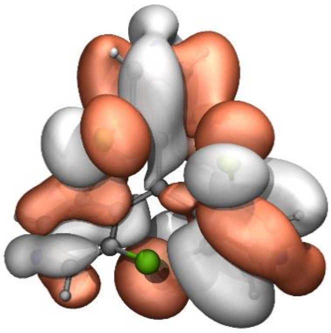<br>111a = –7.34 eV Occ.:1	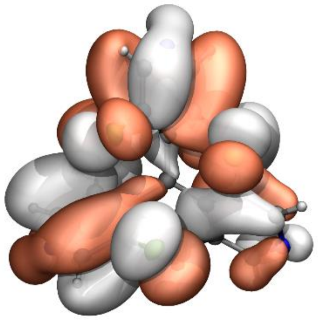<br>111b = –7.30 eV Occ.:1

αHM$_3$TTM

LUMO	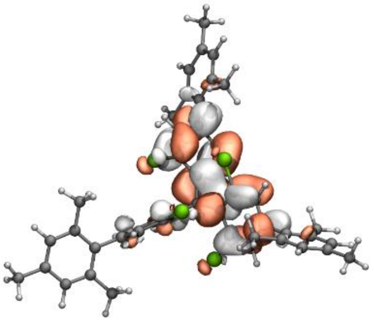 –0.97 eV
HOMO	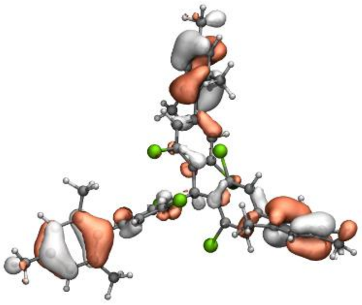 –6.42 eV

CBP

LUMO	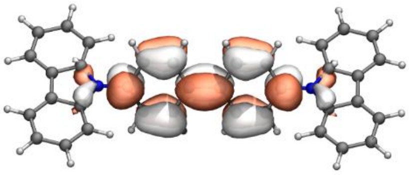 –1.68 eV
HOMO	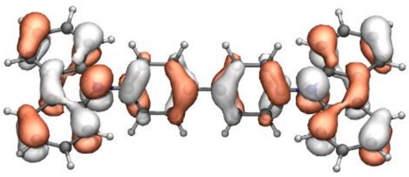 –5.52 eV

Dihedral angles

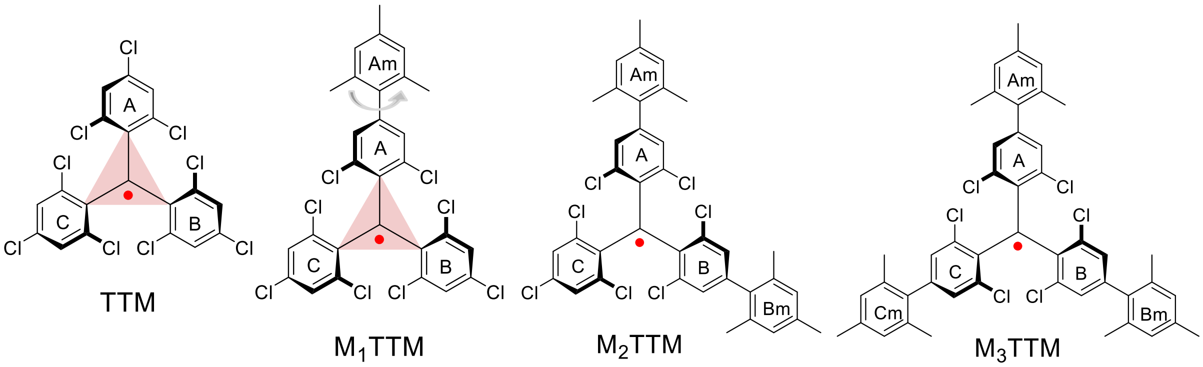

Supplementary Table 4 Dihedral angles of M_xTTM radicals in their single-crystal[a] and DFT-calculated D_0 and D_1 geometries. *A*, *B* and *C* refer to the dihedrals between the ligands and the radical plane (red), *Am*, *Bm* and *Cm* refer to the dihedrals between the mesityl substituents and the ligands.

Radical	Model	*A*	*B*	*C*	*Am*	*Bm*	*Cm*
TTM	SXRD	**47.1**	48.2	48.2	–	–	–
	DFT (D_0/D_1)	47.9/43.8	**47.8/47.6**	47.8/42.7	–	–	–
M_1TTM	SXRD	**45.3**	47.6	47.6	71.0	–	–
	DFT (D_0/D_1)	48.2/44.2	**47.1/47.5**	47.4/43.3	82.3/87.9	–	–
M_2TTM	SXRD	48.5	**50.5**	48.4	78.5	77.1	–
	DFT (D_0/D_1)	47.3/42.8	47.8/42.8	**46.6/47.4**	83.9/87.3	84.1/87.3	–
M_3TTM	SXRD	46.9	46.9	**48.2**	**88.6**	81.5	81.5
	DFT (D_0/D_1)	**46.9/48.2**	47.0/42.9	47.1/42.9	**82.7/56.0**	82.9/87.3	83.2/87.3

[a] See the single-crystal X-ray diffraction data in Supplementary Note 3.

Excited state analysis

Supplementary Table 5 Calculated key parameters for the D_1 state of M_xTTM radicals

Radical	D_1[a]	E (eV)	*f*	D_idx[b]	S_r[c]	t_idx[d]	Orbital config.
TTM	vert.	2.86	0.0047	0.237	0.909	–1.948	Hβ–Sβ: 40.1%; Sα–Lα: 9.7%; Sα–L+1α: 6.7%; H2β–Sβ: 6.1%
	adia.	2.67	0.0105	0.491	0.864	–1.647	Hβ–Sβ: 62.0%; Sα–Lα: 15.4%; Hβ–Lβ: 5.3%
M_1TTM	vert.	2.87	0.0034	0.200	0.913	–1.298	H–4β–Sβ: 26.9%; Sα–Lα: 17.6%; H–2β–Sβ:16.6%
	adia.	2.68	0.0094	0.455	0.867	–1.514	H–1β–Sβ: 57.5%; Sα–Lα: 16.1%
M_2TTM	vert.	2.88	0.0035	0.218	0.913	–2.085	H–7β–Sβ: 28.0%; H–4β–Sβ: 17.7%; Sα–Lα: 17.5%
	adia.	2.69	0.0082	0.419	0.876	–1.734	Hβ–Sβ: 58.0%; Sα–Lα: 17.1%; Hβ–Lβ: 5.2%
M_3TTM	vert.	2.92	0.0052	0.172	0.903	–1.830	H–9β–Sβ: 28.2%; H–6β–Sβ: 15.0%; Sα–Lα: 14.8%
	adia.	2.69	0.0148	0.533	0.861	–1.933	Hβ–Sβ: 45.0%; Sα–Lα: 16.6%; H–8β–Sβ: 8.0%; H–10β–Sβ: 7.3%; Hβ–Lβ: 5.5%

[a] Vertical and adiabatic excited state based on optimized D_0 and D_1 geometry, respectively. [b] Distance between centroid of hole and electron. [c] Overlap index of hole-electron distribution. [d] Separate index of hole-electron distribution.

TTM

	D_1-vert.	D_1-adia.
electron	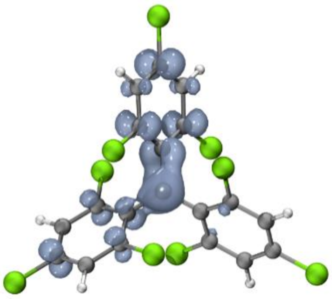<br>**SUMOβ: 48.4%**<br>**LUMOα: 12.2%**	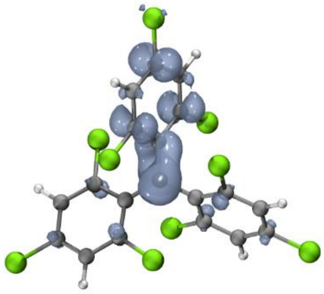<br>**SUMOβ: 63.7%**<br>**LUMOα: 19.4%**
hole	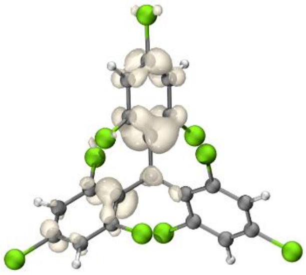<br>**HOMOβ: 42.4%**<br>**SOMOα: 16.9%**	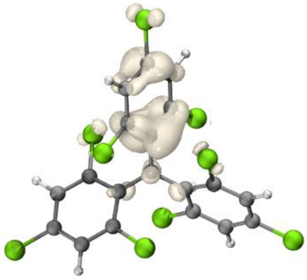<br>**HOMOβ: 62.1%**<br>**SOMOα: 15.7%**

M_1TTM

	D_1-vert.	D_1-adia.
electron	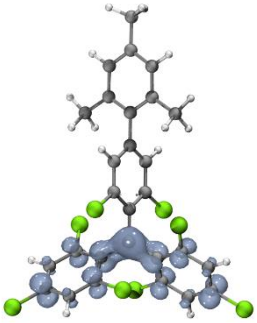<br>**SUMOβ: 46.8%**<br>**LUMOα: 20.2%**	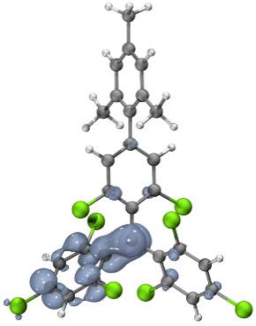<br>**SUMOβ: 62.4%**<br>**LUMOα: 20.1%**
hole	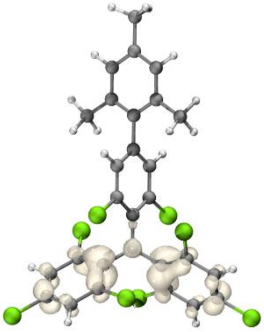<br>**HOMO–2β: 28.8%**<br>**HOMOβ: 17.2%**<br>**SOMOα: 18.1%**	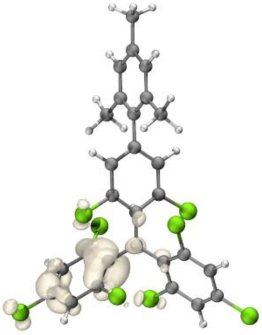<br>**HOMOβ: 62.1%**<br>**SOMOα: 16.2%**

M$_2$TTM

	D_1-vert.	D_1-adia.
electron	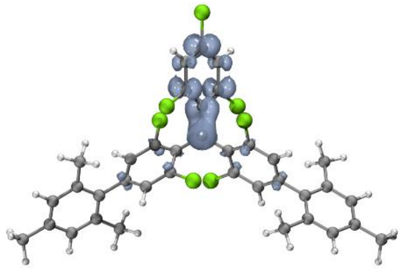 **SUMOβ: 46.9%** **LUMOα: 20.9%**	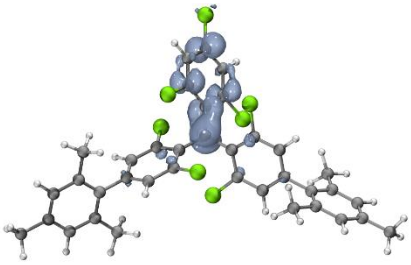 **SUMOβ: 61.3%** **LUMOα: 21.6%**
hole	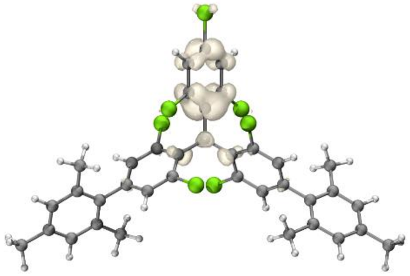 **HOMO–3β: 30.6%** **HOMOβ: 19.2%** **SOMOα : 17.2%**	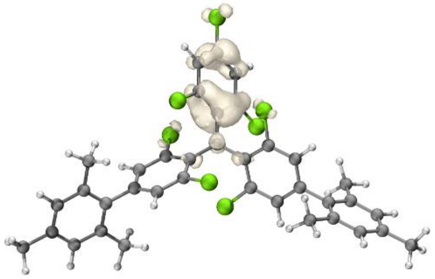 **HOMOβ: 62.6%** **SOMOα: 17.3%**

M$_3$TTM

	D_1-vert.	D_1-adia.
electron	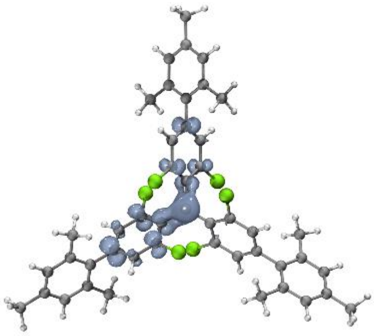 **SUMOβ: 48.1%** **LUMOα: 16.9%**	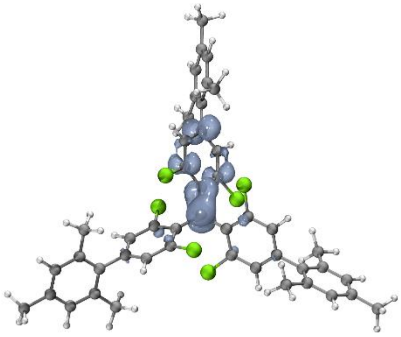 **SUMOβ: 61.5%** **LUMOα: 20.5%**
hole	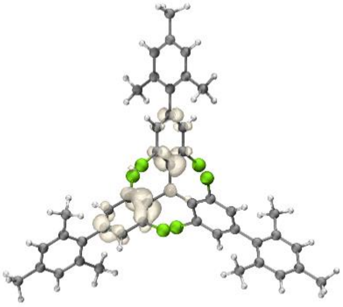 **HOMO–3β: 29.9%** **HOMOβ: 16.7%** **SOMOα: 16.4%**	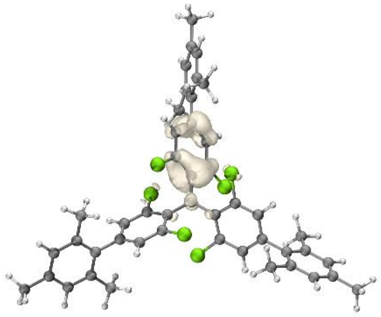 **HOMOβ: 50.2%** **SOMOα: 17.1%**

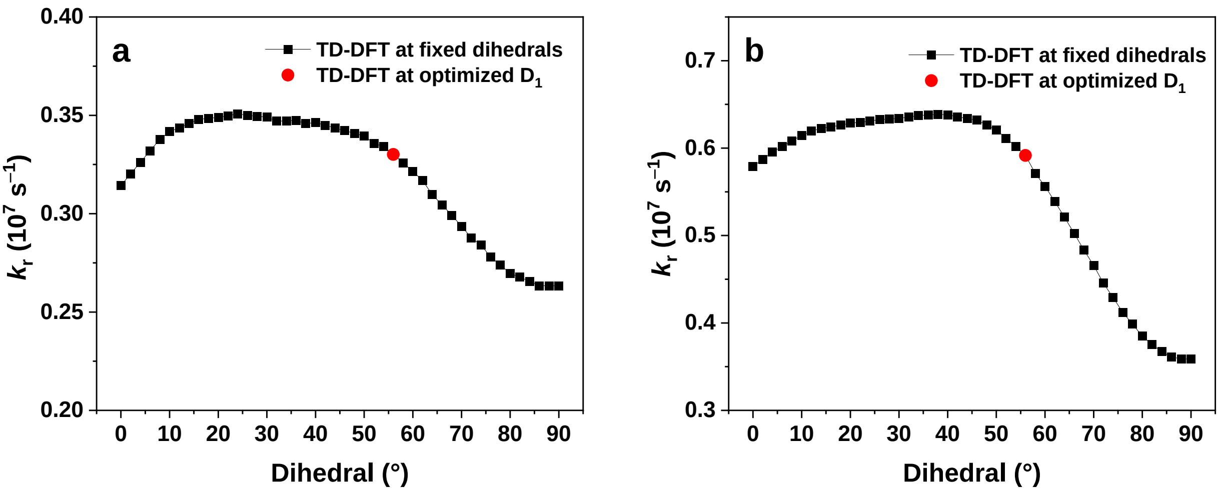

Supplementary Fig. 23 Relationship between radiative decay rate and dihedral *Am* of M_3TTM. Dihedrals *Bm* and *Cm* (see Supplementary Table 4) were manually set at 90° as calculated **a**, at TD-DFT//UCAM-B3LYP/Def2-TZVP level and **b**, at TD-DFT//UPBE0/Def2-TZVP level.

ADCH (atomic dipole moment corrected Hirshfeld) charge analysis

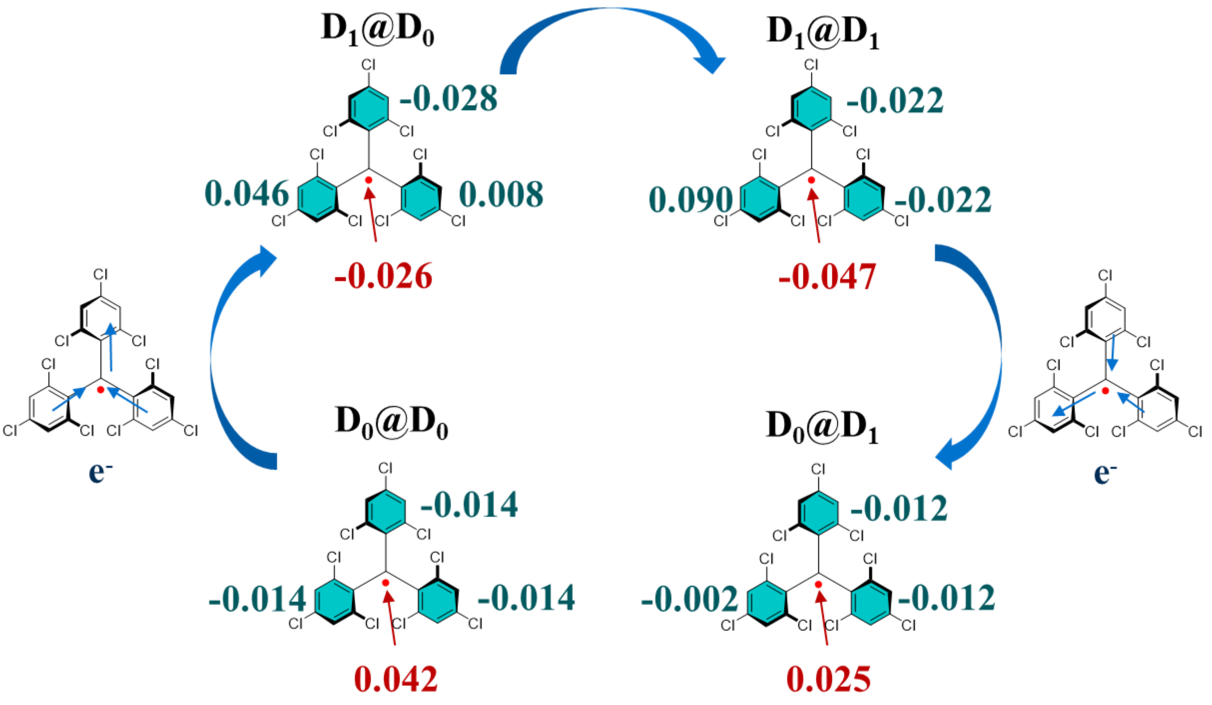

Supplementary Fig. 24 ADCH charge analysis for TTM. Ligand-to-centre CT following $D_0 \rightarrow D_1$ excitation (left), D_1 state relaxation (top) and centre-to-ligand CT following $D_1 \rightarrow D_0$ emission (right).

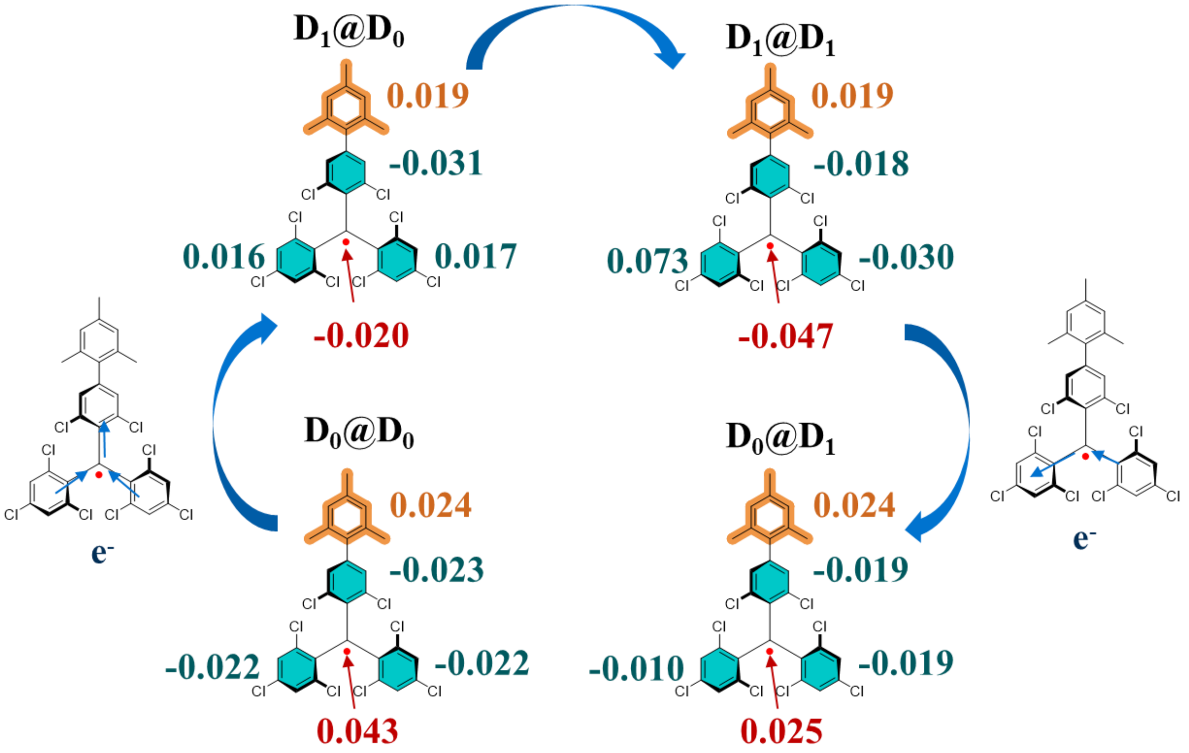

Supplementary Fig. 25 ADCH charge analysis for M_1TTM. Ligand-to-centre CT following $D_0 \rightarrow D_1$ excitation (left), D_1 state relaxation (top) and centre-to-ligand CT following $D_1 \rightarrow D_0$ emission (right).

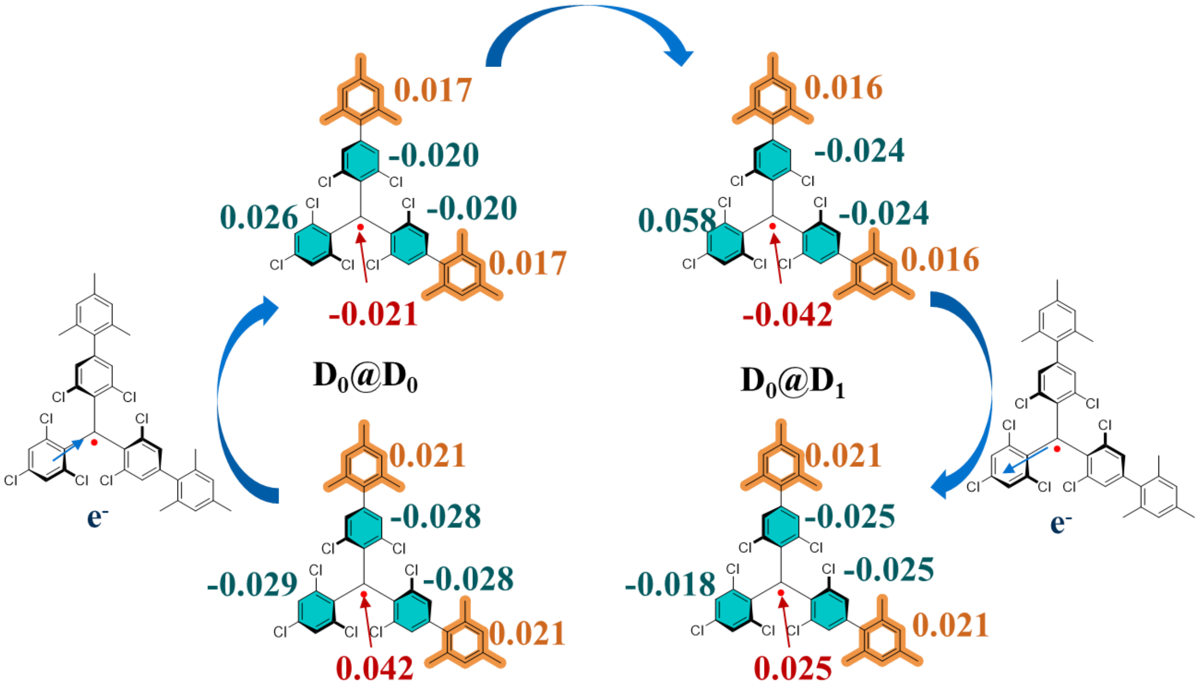

Supplementary Fig. 26 ADCH charge analysis for M_2TTM. Ligand-to-centre CT following $D_0 \rightarrow D_1$ excitation (left), D_1 state relaxation (top) and centre-to-ligand CT following $D_1 \rightarrow D_0$ emission (right).

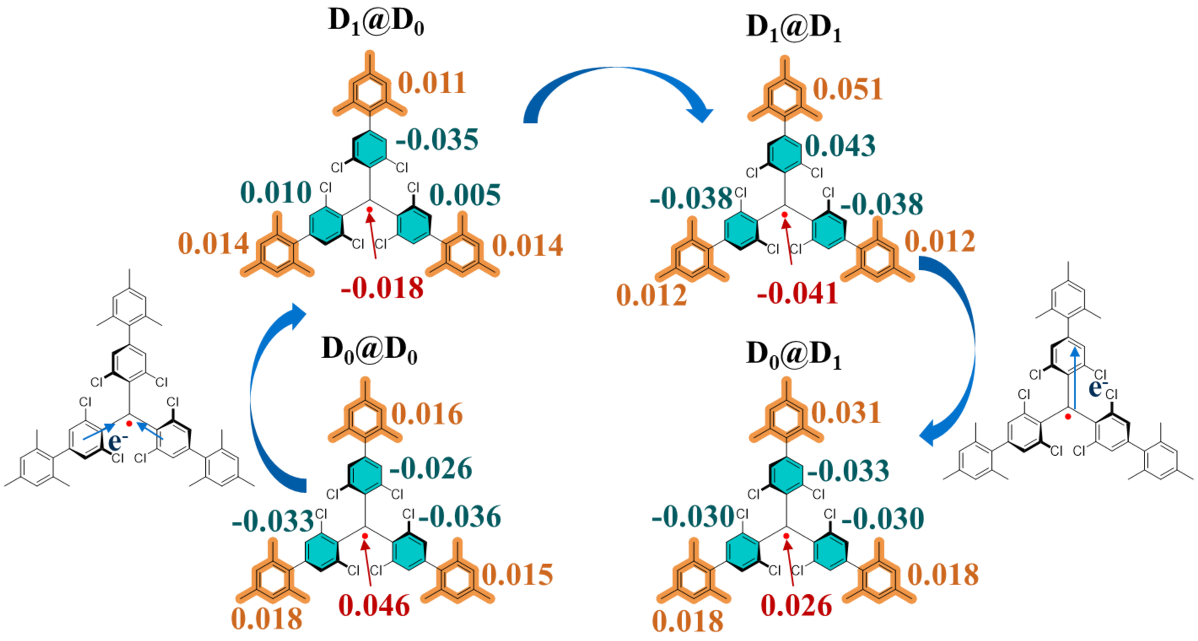

Supplementary Fig. 27 ADCH charge analysis for M_3TTM. Ligand-to-centre CT following $D_0 \rightarrow D_1$ excitation (left), D_1 state relaxation (top) and centre-to-ligand CT following $D_1 \rightarrow D_0$ emission (right).

Supplementary Note 6: Cyclic Voltammetry

Electrochemical redox properties. Cyclic voltammetry on the M_xTTM series indicated a systematic shift of oxidation and reduction potentials (E_{ox} and E_{red}, respectively) toward shallower voltage along with increasing mesityl substitution (see Supplementary Fig. 28). The electrochemical redox reactions are attributed to one-electron oxidation and reduction of the first accessible molecular orbital, *i.e.*, SOMO. Each mesitylation step made the redox reactions more reversible indicating that the bulky substituents not only added to the stability of neutral radicals, but they also stabilized electrochemically oxidized and reduced positively and negatively charged species, respectively. Coupling PCz donor to M_2TTM-3PCz shifted the E_{ox} to shallower potential by another 0.4 V, in line with the electronic push effect of the donor group, while the irreversible oxidation of PCz unit, that is, HOMO, was clearly distinguished from the reversible radical-centred redox reactions (Supplementary Fig. 29). CBP oxidation closely resembled that of the PCz unit in M_2TTM-3PCz (Supplementary Fig. 30), whereas for the M_xTTM series, another oxidation reaction was found beyond the background oxidation of DCM electrolyte (we note that such background oxidation was not observed in solvents like MeCN with a wider electrochemical potential window, but where the samples were not sufficiently soluble). This oxidation reaction is indicative of HOMO energy of M_xTTM being far deeper than that of CBP (Supplementary Fig. 31). Solid-state films of PFMTTM and PCzMTTM featured single reduction and oxidation waves that were stabilized (somewhat shifted to deeper potentials) with respect to the small molecular radicals suggesting uniform redox reactions along the conjugated backbones where injected charges can delocalise (see Supplementary Fig. 32). The polyradicals showed remarkably stable and reversible redox behaviour in both cathodic and anodic potential ranges, and the number of full redox cycles between –1.3 and 1.0 V was limited only by the high solubility of their polyanionic and polycationic forms (*i.e.*, leaching of the reduced and oxidized polymer films, respectively, observed as reduced peak currents after 20 cycles).

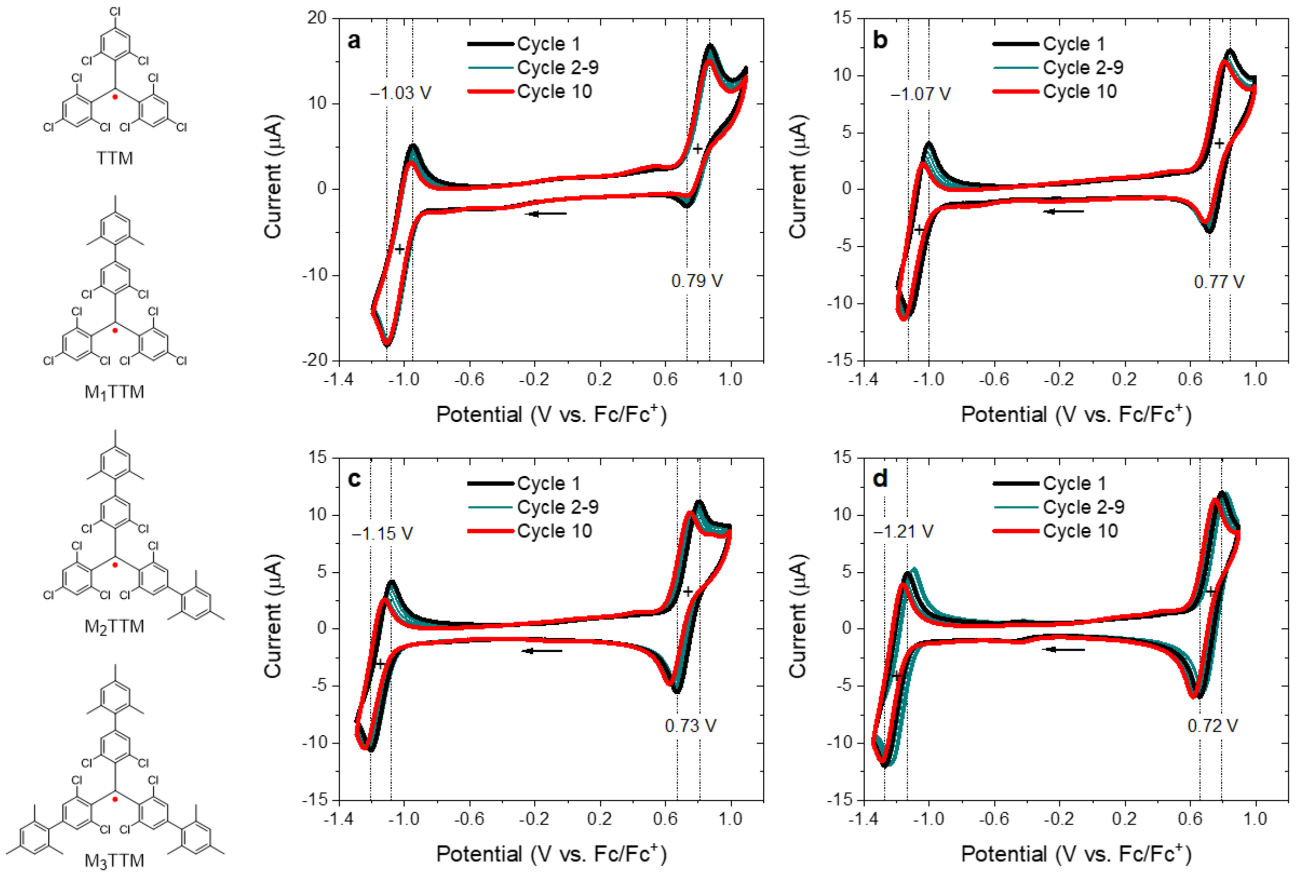

Supplementary Fig. 28 Cyclic voltammetry for solution samples. a, TTM, **b**, M_1TTM, **c**, M_2TTM and **d**, M_3TTM showing 10 full redox cycles. Reduction and oxidation potentials are reported for the first cycle as an average of the forward and reverse peak potentials in the cathodic and anodic ranges, respectively. The redox potentials are indicated by the black plus signs, while the peak potentials are indicated by the black dotted lines. The arrow indicates the scan direction. Supporting electrolyte was 0.1 M Bu_4NPF_6 in THF (anhyd.), scan rate was 100 mV/s on GC WE and sample concentration was 10^{-5} M.

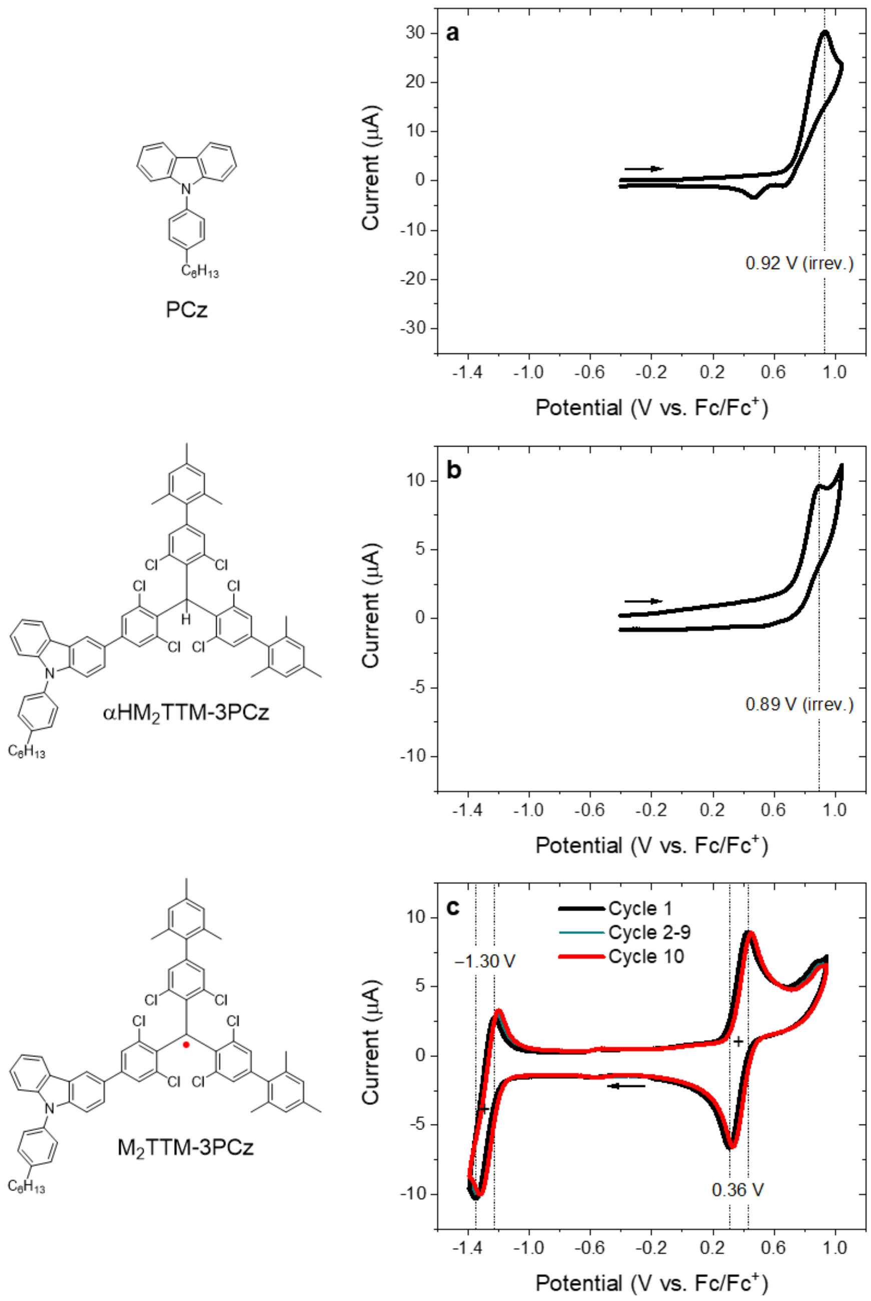

Supplementary Fig. 29 Cyclic voltammetry for solution samples. a, PCz, **b**, αHM_2TTM-3PCz and **c**, M_2TTM-3PCz. For **a**,**b**, oxidation peak potentials are reported and the oxidation reactions are interpreted irreversible. For **c**, reduction and oxidation potentials are reported for the first full redox cycle as an average of the forward and reverse peak potentials in the cathodic and anodic ranges, respectively. The redox potentials are indicated by the black plus signs, while the peak potentials are indicated by the black dotted lines. The arrow indicates the scan direction. Supporting electrolyte was 0.1 M Bu_4NPF_6 in THF (anhyd.), scan rate was 100 mV/s on GC WE and sample concentration was 10^{-5} M.

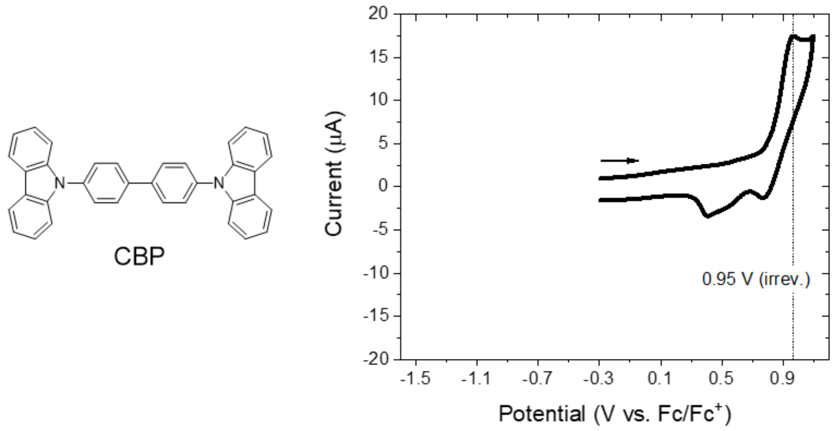

Supplementary Fig. 30 Cyclic voltammetry for a solution sample of CBP. The oxidation peak potential is reported. The oxidation reaction is interpreted irreversible. The peak potential is indicated by the black dotted line. The arrow indicates the scan direction. Supporting electrolyte was 0.1 M Bu_4NPF_6 in THF (anhyd.), scan rate was 100 mV/s on GC WE and sample concentration was 10^{-5} M.

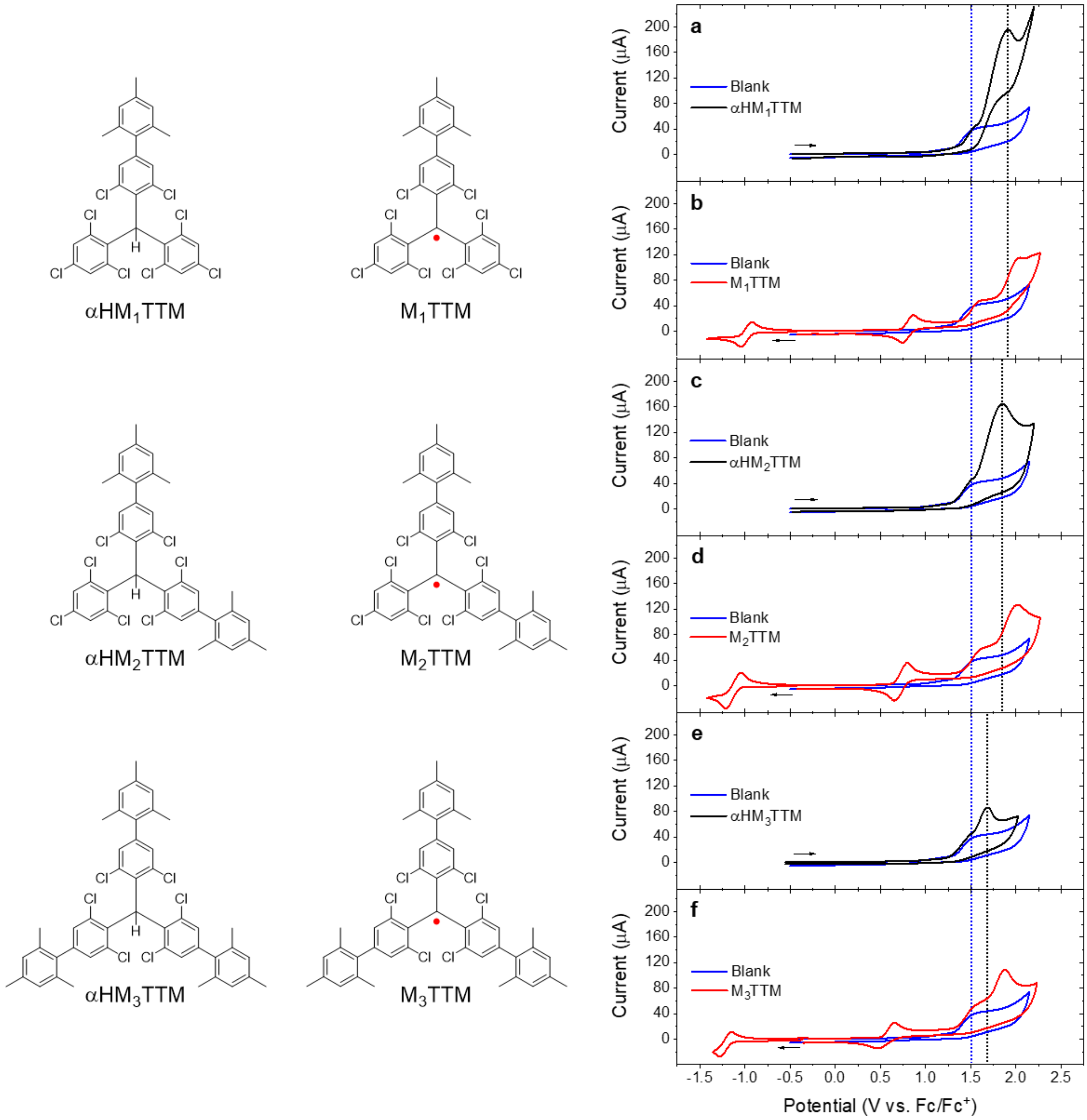

Supplementary Fig. 31 Cyclic voltammetry for solution samples. **a**, αHM_1TTM, **b**, M_1TTM, **c**, αHM_2TTM, **d**, M_2TTM, **e**, αHM_3TTM and **f**, M_3TTM. Oxidation reaction of αH precursors (black traces) and second oxidation reaction of radicals (red traces) are observed at potentials deeper than background oxidation of DCM electrolyte (blue traces) as indicated by the blue vertical line. The black vertical lines indicate the oxidation of αH precursors and a systematic shift of the second oxidation of oxidized (cationic) radicals toward deeper potential relative to the corresponding αH precursors and are guides to the eye only. The arrow indicates the scan direction. Supporting electrolyte was 0.1 M Bu_4NPF_6 in DCM (anhyd.), scan rate was 100 mV/s on Pt WE and sample concentration was 10^{-5} M.

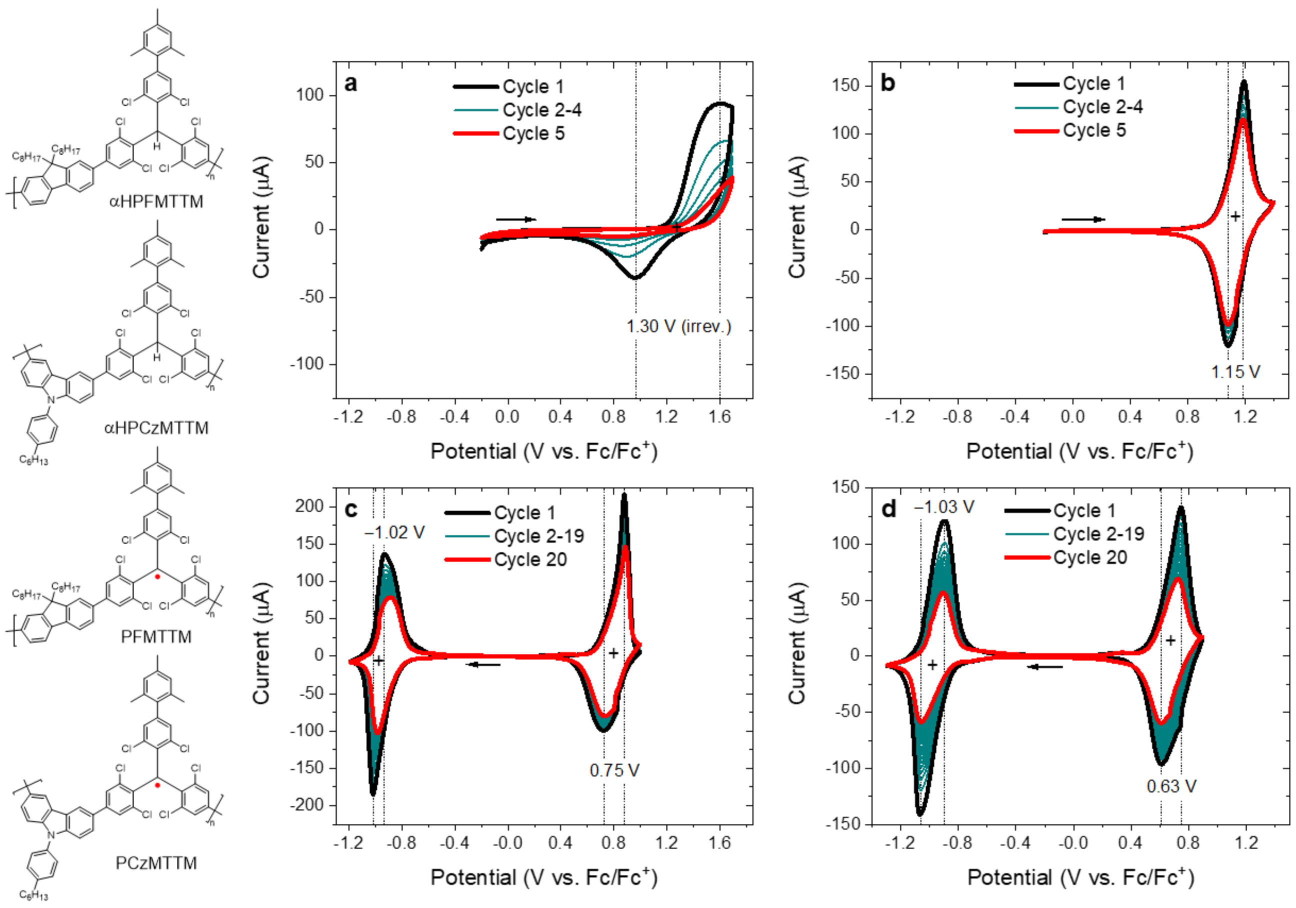

Supplementary Fig. 32 Cyclic voltammetry in solid-state films. a, αHPFMTTM, **b**, αHPCzMTTM, **c**, PFMTTM and **d**, PCzMTTM. For **a**,**b**, oxidation potentials are reported for the first oxidation cycle as an average of the forward and reverse peak potentials in the anodic range. The oxidation reaction of (**a**) is interpreted irreversible. For **c**,**d**, reduction and oxidation potentials are reported for the first full redox cycle as an average of the forward and reverse peak potentials in the cathodic and anodic ranges, respectively. The redox potentials are indicated by the black plus signs, while the peak potentials are indicated by the black dotted lines. The arrow indicates the scan direction. Supporting electrolyte was 0.1 M Bu_4NPF_6 in MeCN (anhyd.) and scan rate was 100 mV/s on Pt wire WE.

Supplementary Note 7: Optical Spectroscopy

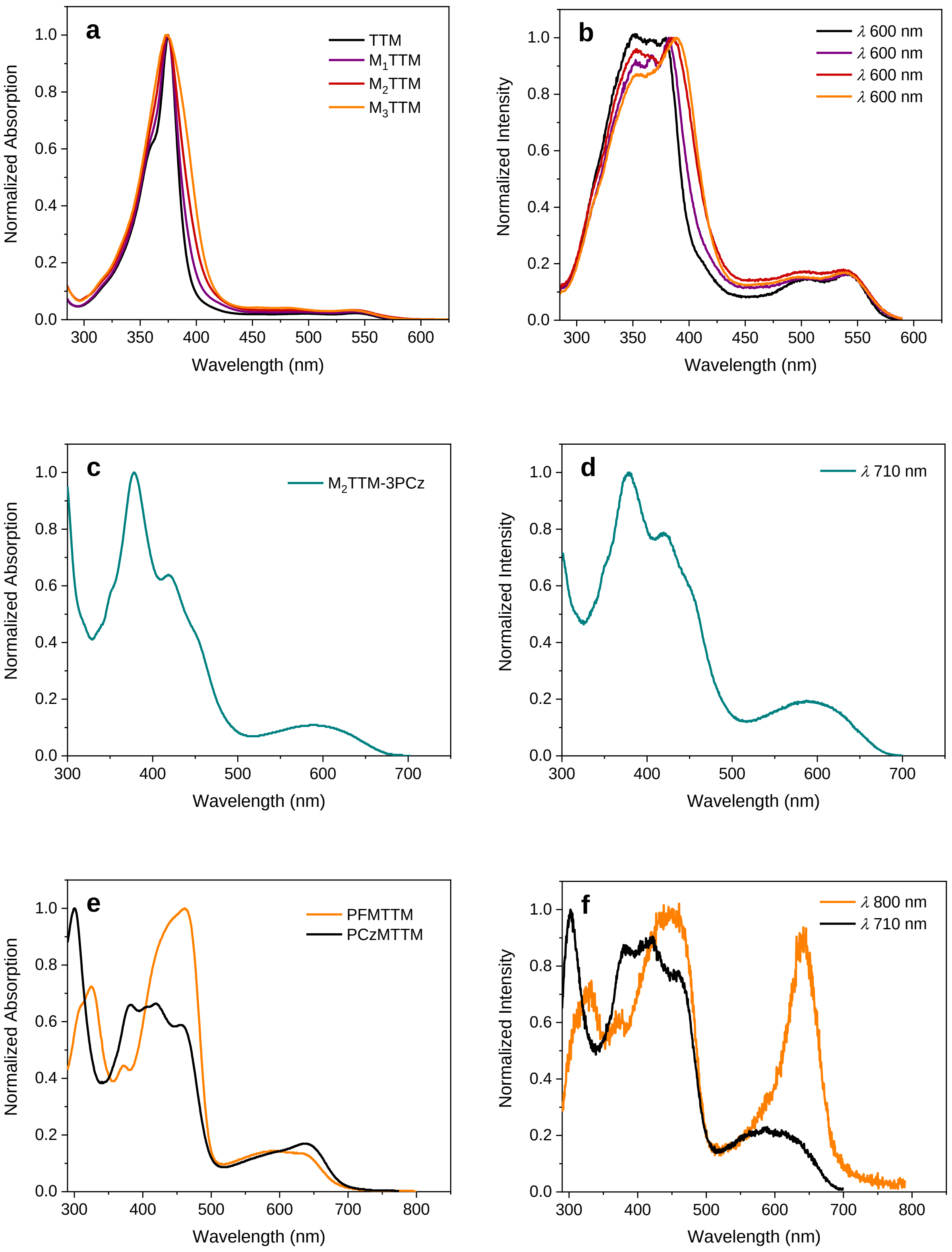

Supplementary Fig. 33 Optical excitation spectroscopy. **a**, **c**, **e**, Absorption and **b**, **d**, **f**, excitation spectra of M_xTTM series, M_2TTM-3PCz, PFMTTM and PCzMTTM in 0.1 mM toluene solution. For **b**, **d**, **f**, the measured emission wavelength for each sample is given in the legend.

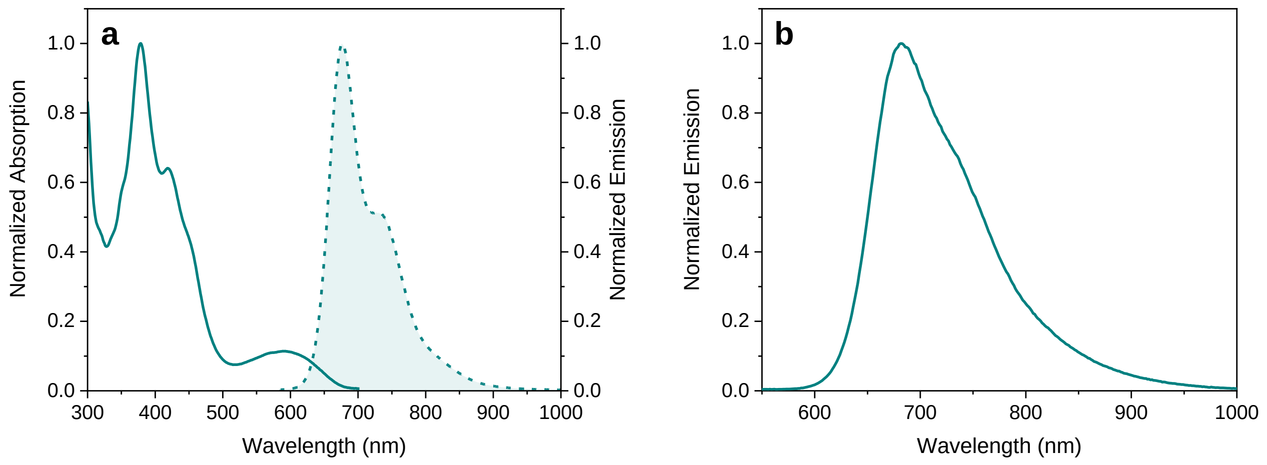

Supplementary Fig. 34 Optical spectroscopy of M_2TTM-3PCz. a, Absorption (solid line) and PL (broken line) spectra in 0.1 mM toluene solution. **b**, Steady-state emission in 10 wt% drop casted film in inert PMMA host.

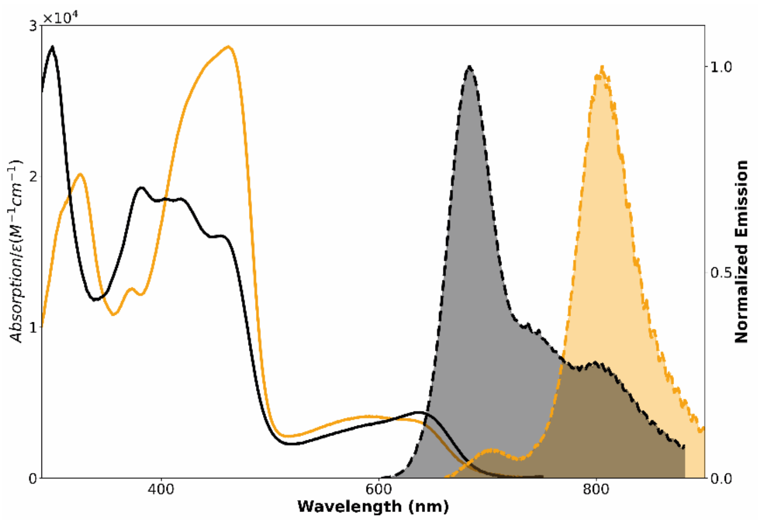

Supplementary Fig. 35 Optical spectroscopy of the polyradicals. Absorption (solid lines) and PL (broken lines) spectra of PFMTTM (yellow) and PCzMTTM (black) in 0.1 mM toluene solution.

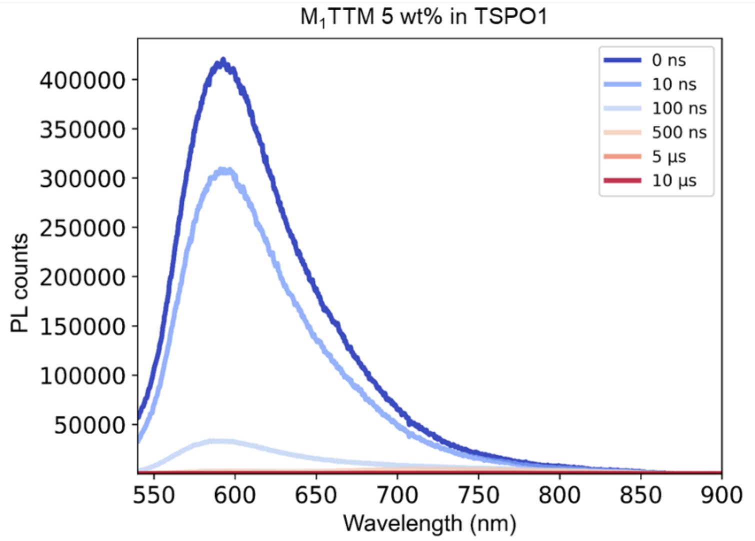

Supplementary Fig. 36 Transient photoluminescence spectra of 5 wt% M_1TTM in TSPO1. Measured at room temperature following 520 nm excitation.

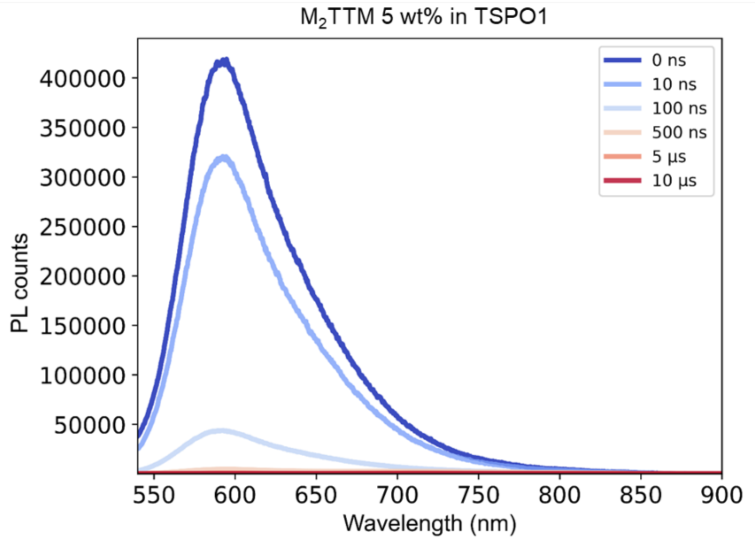

Supplementary Fig. 37 Transient photoluminescence spectra of 5 wt% M_2TTM in TSPO1. Measured at room temperature following 520 nm excitation.

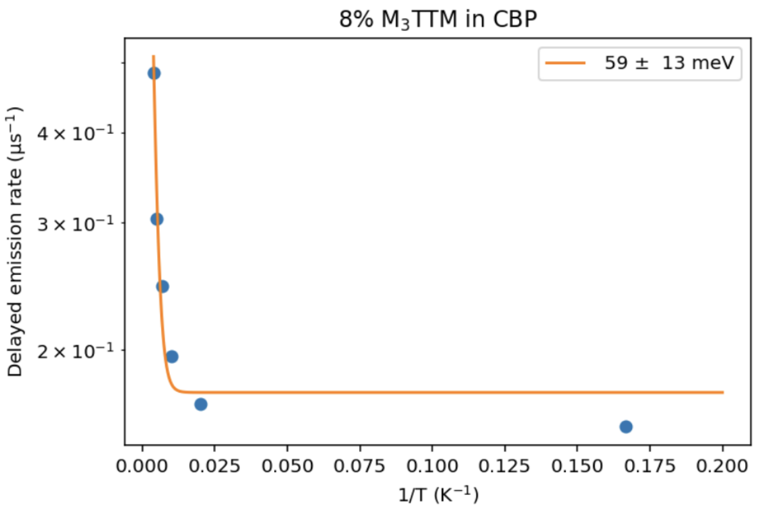

Supplementary Fig. 38 Temperature dependent photoluminescence rate of 8 wt% M_3TTM in CBP. Measured for monomolecular emission in the 570–590 nm region.

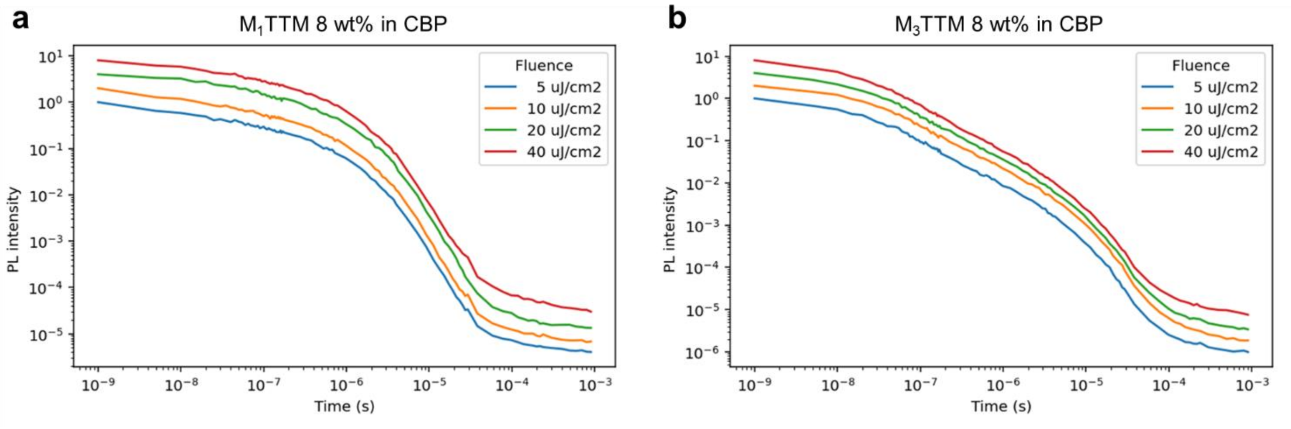

Supplementary Fig. 39 Fluence dependence of luminescence dynamics. a, 8 wt% M_1TTM in CBP and **b**, 8 wt% M_3TTM in CBP at room temperature following 520 nm excitation.

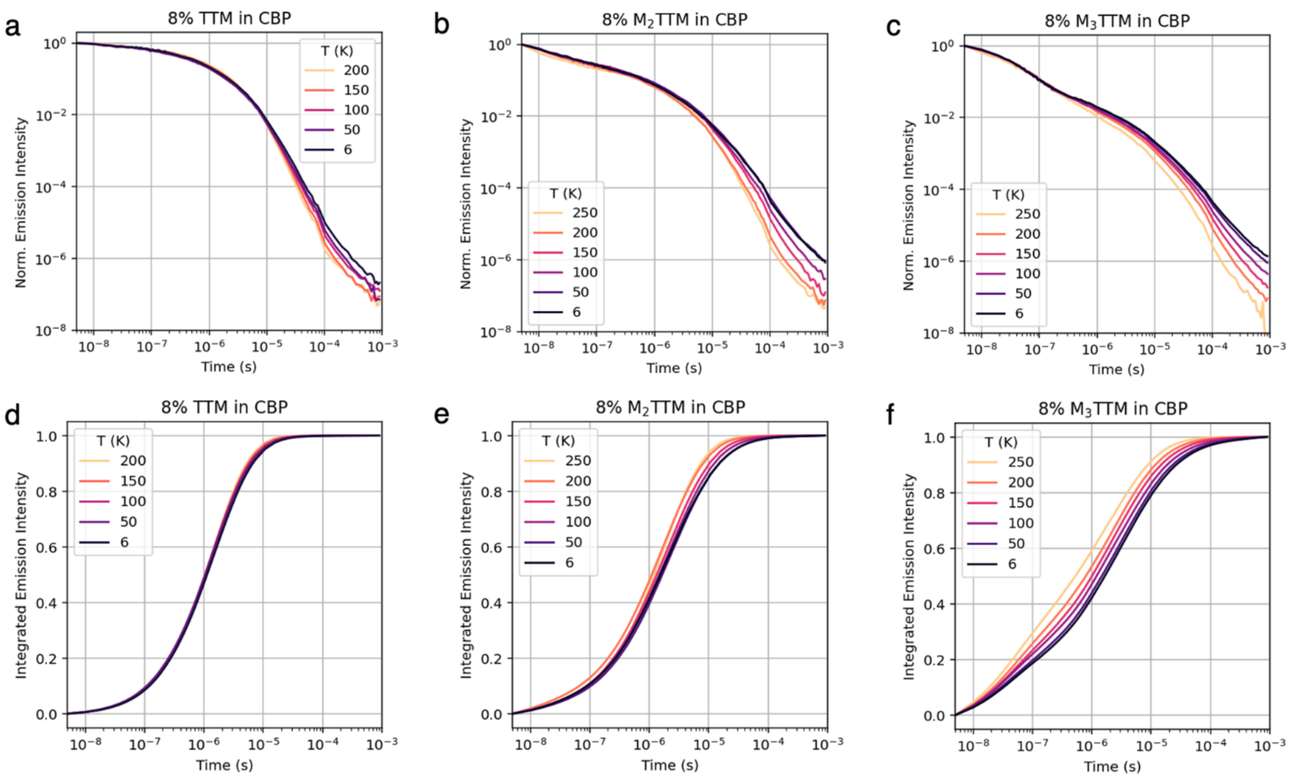

Supplementary Fig. 40 Temperature dependent transient photoluminescence kinetics. a–**c**, Normalised kinetic profiles in the 600–850 nm region following 520 nm excitation. **d**–**f**, Normalised integrated emission intensity dynamics showing the fraction of all emitted photons as a function of time after excitation at 520 nm.

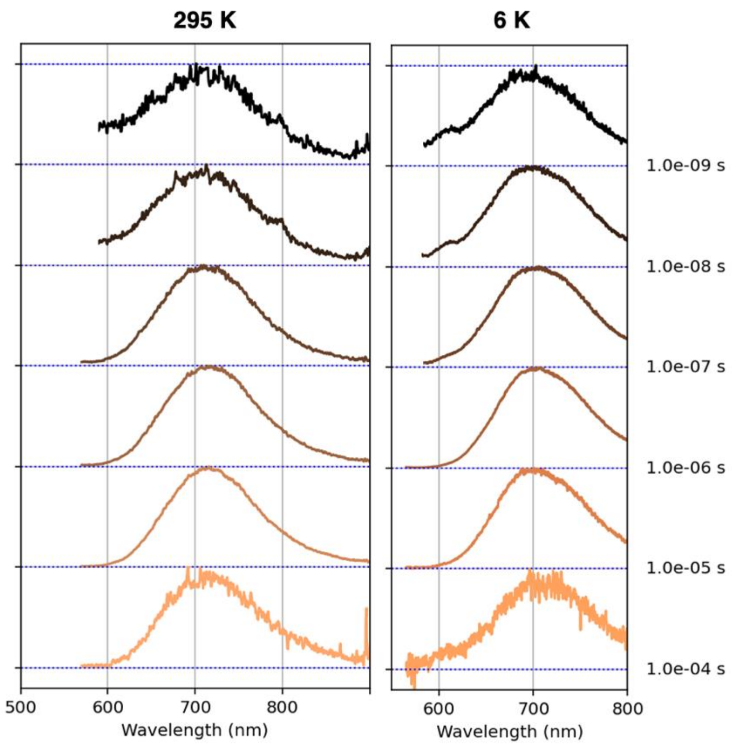

Supplementary Fig. 41 Transient photoluminescence spectra of 8 wt% TTM in CBP. Measured at room and low temperature following 520 nm excitation.

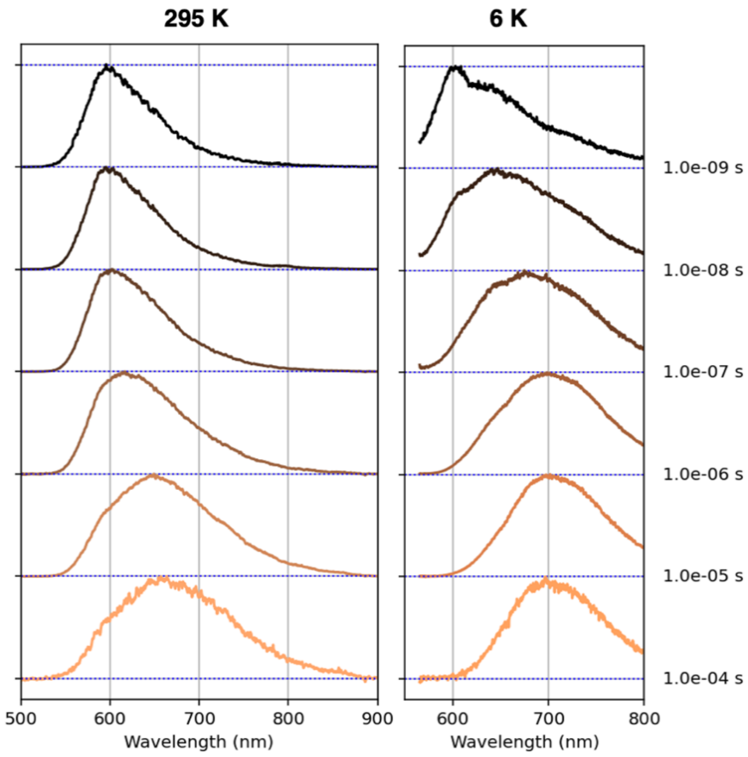

Supplementary Fig. 42 Transient photoluminescence spectra of 8 wt% M_2TTM in CBP. Measured at room and low temperature following 520 nm excitation.

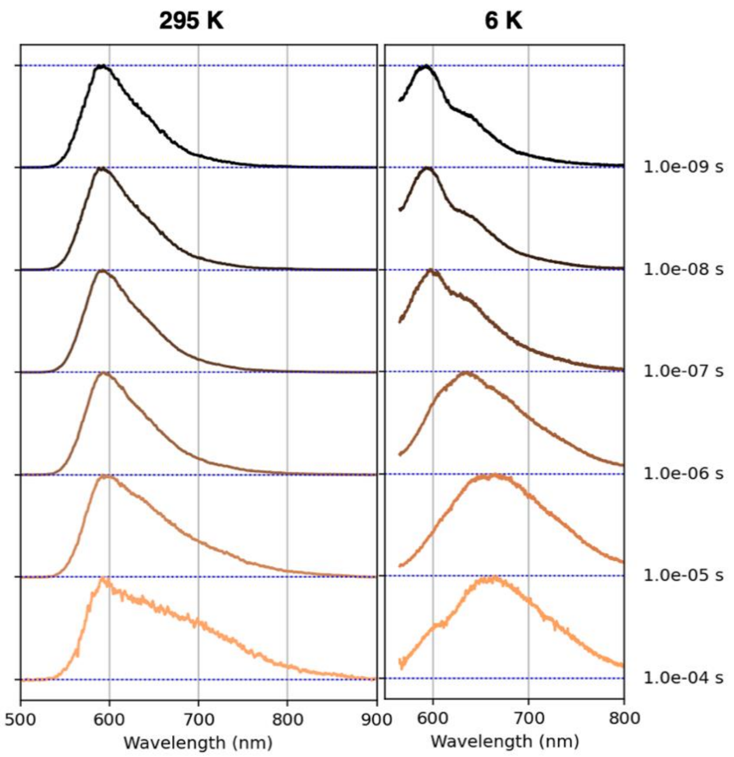

Supplementary Fig. 43 Transient photoluminescence spectra of 8 wt% M_3TTM in CBP. Measured at room and low temperature following 520 nm excitation.

Supplementary Note 8: Thermal Stability

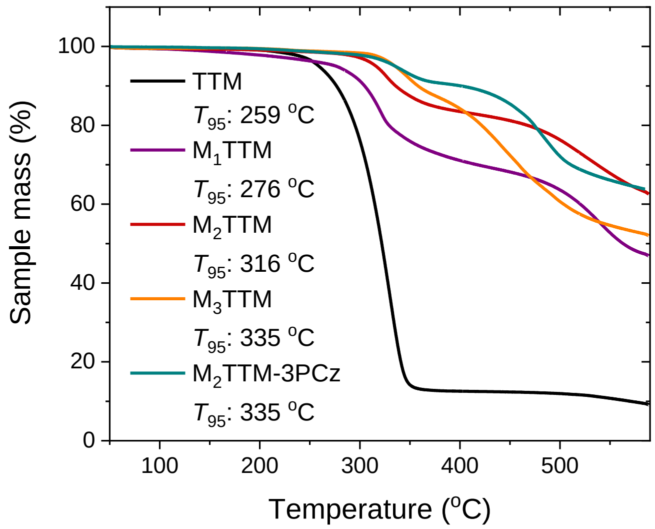

Supplementary Fig. 44 TGA of M_xTTM and M_2TTM-3PCz. Temperature at 5% mass loss (T_{95}) is reported in the legend. The heating rate was 10 °C/min.

Supplementary References

1 Hattori, Y., Kusamoto, T. & Nishihara, H. Luminescence, Stability, and Proton Response of an Open-Shell (3,5-Dichloro-4-pyridyl)bis(2,4,6-trichlorophenyl)methyl Radical. *Angew. Chem. Int. Ed.* **53**, 11845–11848 (2014).

2 Kimura, S. et al. A luminescent organic radical with two pyridyl groups: high photostability and dual stimuli-responsive properties, with theoretical analyses of photophysical processes. *Chem. Sci.* **9**, 1996–2007 (2018).

3 Kimura, S. et al. An Open-shell, Luminescent, Two-Dimensional Coordination Polymer with a Honeycomb Lattice and Triangular Organic Radical. *J. Am. Chem. Soc.* **143**, 4329–4338 (2021).

4 Guo, H. et al. High stability and luminescence efficiency in donor–acceptor neutral radicals not following the Aufbau principle. *Nat. Mater.* **18**, 977–984 (2019).

5 Trasatti, S. The absolute electrode potential: an explanatory note. *Pure Appl. Chem.* **58**, 955–966 (1986).

6 Cardona, C. M., Li, W., Kaifer, A. E., Stockdale, D. & Bazan, G. C. Electrochemical Considerations for Determining Absolute Frontier Orbital Energy Levels of Conjugated Polymers for Solar Cell Applications. *Adv. Mater.* **23**, 2367–2371 (2011).

7 Jeon, S. O., Jang, S. E., Son, H. S. & Lee, J. Y. External Quantum Efficiency Above 20% in Deep Blue Phosphorescent Organic Light-Emitting Diodes. *Adv. Mater.* **23**, 1436–1441 (2011).

8 Peng, Q., Obolda, A., Zhang, M. & Li, F. Organic Light-Emitting Diodes Using a Neutral π Radical as Emitter: The Emission from a Doublet. *Angew. Chem. Int. Ed.* **54**, 7091–7095 (2015).

9 Roques, N. et al. Three-Dimensional Six-Connecting Organic Building Blocks Based on Polychlorotriphenylmethyl Units—Synthesis, Self-Assembly, and Magnetic Properties. *Chem. Eur. J.* **12**, 9238–9253 (2006).

10 Maddala, S. et al. Forming a Metal-Free Oxidatively Coupled Agent, Bicarbazole, as a Defect Passivation for HTM and an Interfacial Layer in a p–i–n Perovskite Solar Cell Exhibits Nearly 20% Efficiency. *Chem. Mater.* **32**, 127–138 (2020).

11 Armet, O. et al. Inert carbon free radicals. 8. Polychlorotriphenylmethyl radicals: synthesis, structure, and spin-density distribution. *J. Phys. Chem.* **91**, 5608–5616 (1987).

12 Spek, A. PLATON SQUEEZE: a tool for the calculation of the disordered solvent contribution to the calculated structure factors. *Acta Cryst.* **C71**, 9–18 (2015).

13 Tesio, A. Y. et al. Organic radicals for the enhancement of oxygen reduction reaction in Li–O2 batteries. *Chem. Commun.* **51**, 17623–17626 (2015).

14 Hilborn, R. C. Einstein coefficients, cross sections, f values, dipole moments, and all that. *Am. J. Phys.* **50**, 982–986 (1982).

15 Abroshan, H., Coropceanu, V. & Brédas, J.-L. Radiative and Nonradiative Recombinations in Organic Radical Emitters: The Effect of Guest–Host Interactions. *Adv. Funct. Mater.* **30**, 2002916 (2020).

16 Uejima, M., Sato, T., Yokoyama, D., Tanaka, K. & Park, J.-W. Quantum yield in blue-emitting anthracene derivatives: vibronic coupling density and transition dipole moment density. *Phys. Chem. Chem. Phys.* **16**, 14244–14256 (2014).

17 Zeng, W., Gong, S., Zhong, C. & Yang, C. Prediction of Oscillator Strength and Transition Dipole Moments with the Nuclear Ensemble Approach for Thermally Activated Delayed Fluorescence Emitters. *J. Phys. Chem. C* **123**, 10081–10086 (2019).

18 Abroshan, H., Winget, P., Kwak, H. S., Brown, C. T. & Halls, M. D. Organic radical emitters: nature of doublet excitons in emissive layers. *Phys. Chem. Chem. Phys.* **24**, 16891–16899 (2022).

19 Abdurahman, A. et al. Understanding the luminescent nature of organic radicals for efficient doublet emitters and pure-red light-emitting diodes. *Nat. Mater.* **19**, 1224–1229 (2020).
