# Supplementary material for: Mesitylated trityl radicals, a platform for doublet emission: symmetry breaking, charge-transfer states and conjugated polymers
Source: Nat Commun. 2023 Jul 12;14:4147. doi: 10.1038/s41467-023-39834-2 (PMC10338495; doi:10.1038/s41467-023-39834-2)

checkCIF/PLATON report

Structure factors have been supplied for datablock(s) M2_TTM

THIS REPORT IS FOR GUIDANCE ONLY. IF USED AS PART OF A REVIEW PROCEDURE FOR PUBLICATION, IT SHOULD NOT REPLACE THE EXPERTISE OF AN EXPERIENCED CRYSTALLOGRAPHIC REFEREE.

No syntax errors found. CIF dictionary Interpreting this report

Datablock: M2_TTM

```
Bond precision:   C-C = 0.0045 A          Wavelength=1.54178

Cell:              a=15.9187(4)      b=20.6752(5)      c=11.6563(3)
                   alpha=90          beta=90           gamma=90
Temperature:       180 K

                Calculated                    Reported
Volume          3836.35(17)                   3836.35(17)
Space group     P n a 21                      P n a 21
Hall group      P 2c -2n                      P 2c -2n
Moiety formula  C37 H28 Cl7, C H Cl3          C37 H28 Cl7, C H Cl3
Sum formula     C38 H29 Cl10                  C38 H29 Cl10
Mr              840.11                        840.11
Dx,g cm-3       1.455                         1.455
Z               4                             4
Mu (mm-1)       6.864                         6.864
F000            1708.0                        1708.0
F000'           1725.45
h,k,lmax        18,24,13                      18,24,13
Nref            6765[ 3565]                   6498
Tmin,Tmax       0.225,0.709                   0.480,0.753
Tmin'           0.144

Correction method= # Reported T Limits: Tmin=0.480 Tmax=0.753
AbsCorr = MULTI-SCAN

Data completeness= 1.82/0.96       Theta(max)= 66.581

                                                     wR2(reflections)=
R(reflections)= 0.0263( 6342)                        0.0682( 6498)

S = 1.052                    Npar= 439
```

The following ALERTS were generated. Each ALERT has the format
test-name_ALERT_alert-type_alert-level.
Click on the hyperlinks for more details of the test.

Alert level C

```
PLAT244_ALERT_4_C Low    'Solvent' Ueq as Compared to Neighbors of        C1S Check
PLAT340_ALERT_3_C Low Bond Precision on  C-C Bonds ...............    0.00449 Ang.
PLAT911_ALERT_3_C Missing FCF Refl Between Thmin & STh/L=    0.595         15 Report
PLAT987_ALERT_1_C The Flack x is >> 0 -  Do a BASF/TWIN Refinement     Please Check
```

Alert level G

```
PLAT380_ALERT_4_G Incorrectly? Oriented X(sp2)-Methyl Moiety .....        C35 Check
PLAT434_ALERT_2_G Short Inter HL..HL Contact Cl6      ..Cl7      .       3.34 Ang.
                                              1/2+x,3/2-y,z  =      3_565 Check
PLAT909_ALERT_3_G Percentage of I>2sig(I) Data at Theta(Max) Still        97% Note
PLAT978_ALERT_2_G Number C-C Bonds with Positive Residual Density.          6 Info
```

0 **ALERT level A** = Most likely a serious problem - resolve or explain
0 **ALERT level B** = A potentially serious problem, consider carefully
4 **ALERT level C** = Check. Ensure it is not caused by an omission or oversight
4 **ALERT level G** = General information/check it is not something unexpected

1 ALERT type 1 CIF construction/syntax error, inconsistent or missing data
2 ALERT type 2 Indicator that the structure model may be wrong or deficient
3 ALERT type 3 Indicator that the structure quality may be low
2 ALERT type 4 Improvement, methodology, query or suggestion
0 ALERT type 5 Informative message, check

It is advisable to attempt to resolve as many as possible of the alerts in all categories. Often the minor alerts point to easily fixed oversights, errors and omissions in your CIF or refinement strategy, so attention to these fine details can be worthwhile. In order to resolve some of the more serious problems it may be necessary to carry out additional measurements or structure refinements. However, the purpose of your study may justify the reported deviations and the more serious of these should normally be commented upon in the discussion or experimental section of a paper or in the "special_details" fields of the CIF. checkCIF was carefully designed to identify outliers and unusual parameters, but every test has its limitations and alerts that are not important in a particular case may appear. Conversely, the absence of alerts does not guarantee there are no aspects of the results needing attention. It is up to the individual to critically assess their own results and, if necessary, seek expert advice.

Publication of your CIF in IUCr journals

A basic structural check has been run on your CIF. These basic checks will be run on all CIFs submitted for publication in IUCr journals (*Acta Crystallographica*, *Journal of Applied Crystallography*, *Journal of Synchrotron Radiation*); however, if you intend to submit to *Acta Crystallographica Section C* or *E* or *IUCrData*, you should make sure that full publication checks are run on the final version of your CIF prior to submission.

Publication of your CIF in other journals

Please refer to the *Notes for Authors* of the relevant journal for any special instructions relating to CIF submission.

PLATON version of 18/05/2022; check.def file version of 17/05/2022

Datablock M2_TTM - ellipsoid plot

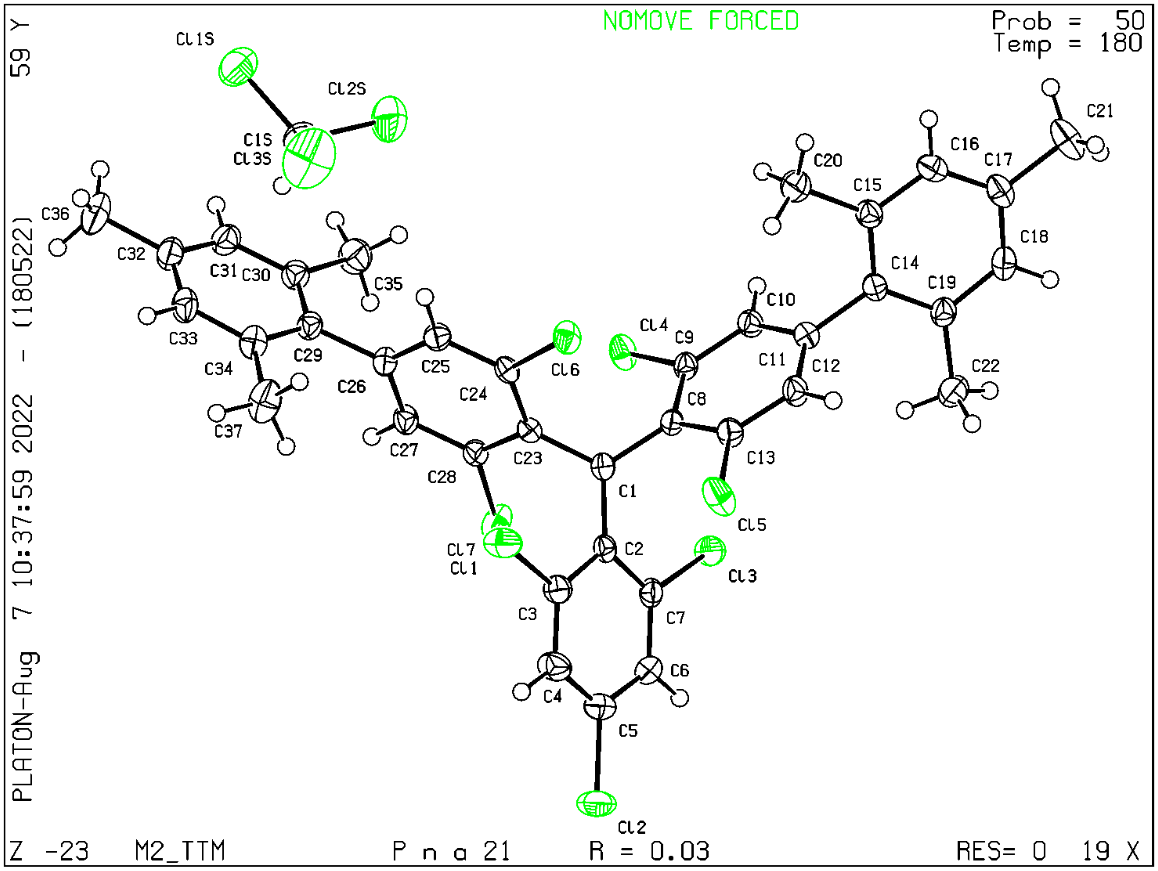

Supplement: Supplementary file 3 — Source Data [file 41467_2023_39834_MOESM3_ESM.zip › CIFs/checkcif_M2TTM.pdf]
