# Supplementary material for: Mesitylated trityl radicals, a platform for doublet emission: symmetry breaking, charge-transfer states and conjugated polymers
Source: Nat Commun. 2023 Jul 12;14:4147. doi: 10.1038/s41467-023-39834-2 (PMC10338495; doi:10.1038/s41467-023-39834-2)

checkCIF/PLATON report

Structure factors have been supplied for datablock(s) M3_TTM

THIS REPORT IS FOR GUIDANCE ONLY. IF USED AS PART OF A REVIEW PROCEDURE FOR PUBLICATION, IT SHOULD NOT REPLACE THE EXPERTISE OF AN EXPERIENCED CRYSTALLOGRAPHIC REFEREE.

No syntax errors found. CIF dictionary Interpreting this report

Datablock: M3_TTM

Bond precision: C-C = 0.0045 A Wavelength=1.54178

Cell:	a=47.638(3)	b=47.638(3)	c=12.9562(12)
	alpha=90	beta=90	gamma=120
Temperature:	180 K		

	Calculated	Reported
Volume	25463(4)	25463(4)
Space group	R -3 c	R -3 c :H
Hall group	-R 3 2"c	-R 3 2"c
Moiety formula	C46 H39 Cl6 [+ solvent]	C46 H39 Cl6 [+ solvent]
Sum formula	C46 H39 Cl6 [+ solvent]	C46 H39 Cl6 [+ solvent]
Mr	804.47	804.47
Dx,g cm-3	0.944	0.944
Z	18	18
Mu (mm-1)	2.941	2.941
F000	7506.0	7506.0
F000'	7560.11	
h,k,lmax	56,56,15	56,56,15
Nref	5027	5020
Tmin,Tmax	0.738,0.790	0.547,0.753
Tmin'	0.561	

Correction method= # Reported T Limits: Tmin=0.547 Tmax=0.753
AbsCorr = MULTI-SCAN

Data completeness= 0.999 Theta(max)= 66.655

R(reflections)= 0.0442(3674) wR2(reflections)= 0.1352(5020)

S = 1.037 Npar= 242

The following ALERTS were generated. Each ALERT has the format
test-name_ALERT_alert-type_alert-level.
Click on the hyperlinks for more details of the test.

Alert level C

```
PLAT213_ALERT_2_C Atom C10              has ADP max/min Ratio .....        3.2 prolat
PLAT220_ALERT_2_C NonSolvent   Resd 1  C   Ueq(max)/Ueq(min) Range         4.1 Ratio
PLAT222_ALERT_3_C NonSolvent Resd 1  H   Uiso(max)/Uiso(min) Range         4.9 Ratio
PLAT242_ALERT_2_C Low    'MainMol' Ueq as Compared to Neighbors of          C9 Check
PLAT340_ALERT_3_C Low Bond Precision on  C-C Bonds ..............      0.00447 Ang.
PLAT905_ALERT_3_C Negative K value in the Analysis of Variance ...      -2.989 Report
PLAT911_ALERT_3_C Missing FCF Refl Between Thmin & STh/L=    0.596           6 Report
```

Alert level G

```
CELLZ01_ALERT_1_G Difference between formula and atom_site contents detected.
CELLZ01_ALERT_1_G ALERT: Large difference may be due to a
            symmetry error - see SYMMG tests
           From the CIF: _cell_formula_units_Z   18
           From the CIF: _chemical_formula_sum  C46 H39 Cl6 [+ solvent]
           TEST: Compare cell contents of formula and atom_site data

           atom    Z*formula  cif sites diff
           C        828.00    828.00    0.00
           H        702.00    702.00    0.00
           Cl       108.00    108.00    0.00
           [+        18.00      0.00   18.00
           solve     18.00      0.00   18.00
PLAT083_ALERT_2_G SHELXL Second Parameter in WGHT  Unusually Large      27.47 Why ?
PLAT300_ALERT_4_G Atom Site Occupancy of H10A       Constrained at        0.5 Check
PLAT300_ALERT_4_G Atom Site Occupancy of H10B       Constrained at        0.5 Check
PLAT300_ALERT_4_G Atom Site Occupancy of H10C       Constrained at        0.5 Check
PLAT367_ALERT_2_G Long?  C(sp?)-C(sp?) Bond  C9      - C10      .        1.52 Ang.
PLAT380_ALERT_4_G Incorrectly? Oriented X(sp2)-Methyl Moiety .....        C26 Check
PLAT606_ALERT_4_G Solvent Accessible VOID(S) in Structure ........          ! Info
PLAT869_ALERT_4_G ALERTS Related to the Use of SQUEEZE  Suppressed          ! Info
PLAT909_ALERT_3_G Percentage of I>2sig(I) Data at Theta(Max) Still        55% Note
PLAT910_ALERT_3_G Missing # of FCF Reflection(s) Below Theta(Min).          1 Note
PLAT913_ALERT_3_G Missing # of Very Strong Reflections in FCF ....          1 Note
PLAT933_ALERT_2_G Number of HKL-OMIT Records in Embedded .res File          2 Note
PLAT965_ALERT_2_G The SHELXL WEIGHT Optimisation has not Converged     Please Check
PLAT978_ALERT_2_G Number C-C Bonds with Positive Residual Density.          3 Info
```

```
   0 ALERT level A = Most likely a serious problem - resolve or explain
   0 ALERT level B = A potentially serious problem, consider carefully
   7 ALERT level C = Check. Ensure it is not caused by an omission or oversight
  16 ALERT level G = General information/check it is not something unexpected

   2 ALERT type 1 CIF construction/syntax error, inconsistent or missing data
   8 ALERT type 2 Indicator that the structure model may be wrong or deficient
   7 ALERT type 3 Indicator that the structure quality may be low
   6 ALERT type 4 Improvement, methodology, query or suggestion
   0 ALERT type 5 Informative message, check
```

It is advisable to attempt to resolve as many as possible of the alerts in all categories. Often the minor alerts point to easily fixed oversights, errors and omissions in your CIF or refinement strategy, so attention to these fine details can be worthwhile. In order to resolve some of the more serious problems it may be necessary to carry out additional measurements or structure refinements. However, the purpose of your study may justify the reported deviations and the more serious of these should normally be commented upon in the discussion or experimental section of a paper or in the "special_details" fields of the CIF. checkCIF was carefully designed to identify outliers and unusual parameters, but every test has its limitations and alerts that are not important in a particular case may appear. Conversely, the absence of alerts does not guarantee there are no aspects of the results needing attention. It is up to the individual to critically assess their own results and, if necessary, seek expert advice.

Publication of your CIF in IUCr journals

A basic structural check has been run on your CIF. These basic checks will be run on all CIFs submitted for publication in IUCr journals (*Acta Crystallographica*, *Journal of Applied Crystallography*, *Journal of Synchrotron Radiation*); however, if you intend to submit to *Acta Crystallographica Section C* or *E* or *IUCrData*, you should make sure that full publication checks are run on the final version of your CIF prior to submission.

Publication of your CIF in other journals

Please refer to the *Notes for Authors* of the relevant journal for any special instructions relating to CIF submission.

PLATON version of 18/05/2022; check.def file version of 17/05/2022

Datablock M3_TTM - ellipsoid plot

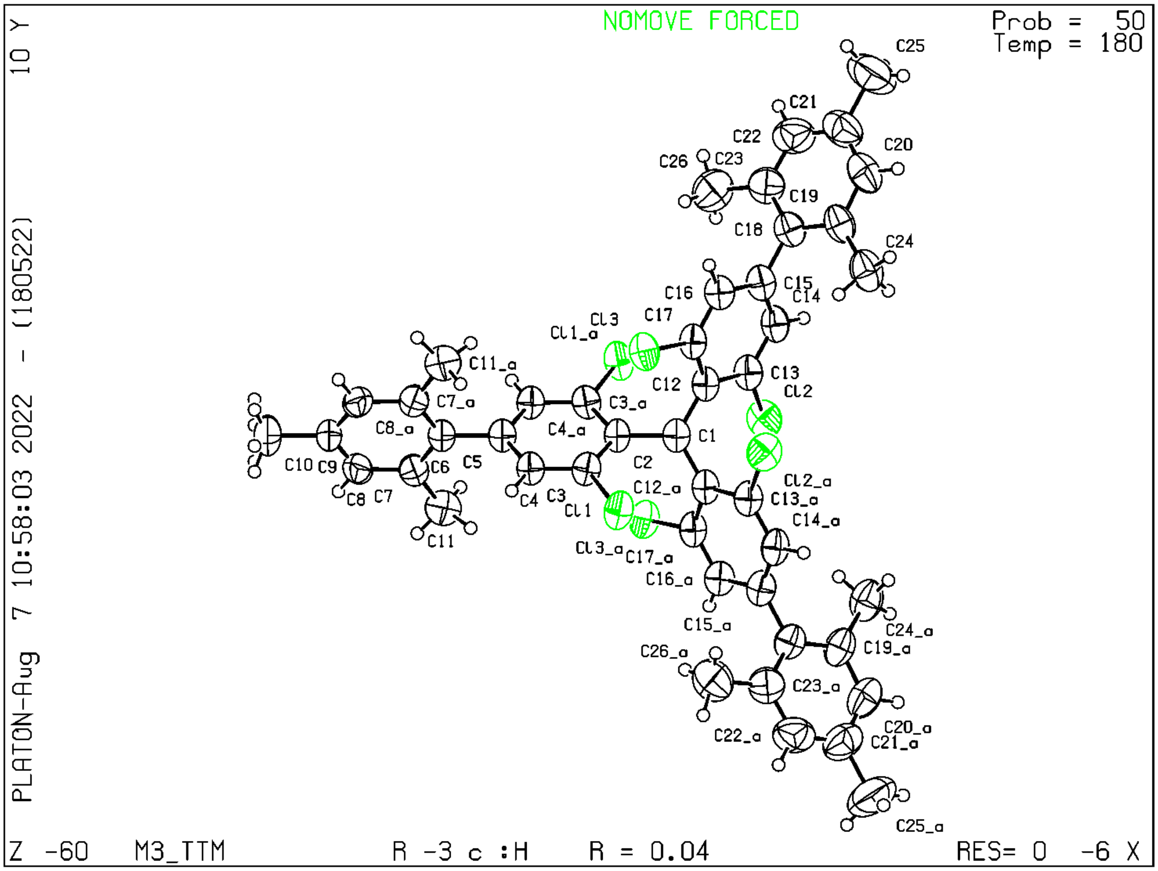

Supplement: Supplementary file 3 — Source Data [file 41467_2023_39834_MOESM3_ESM.zip › CIFs/checkcif_M3TTM.pdf]
